# Exploiting opportunistic observations to estimate changes in seasonal site use: An example with wetland birds

**DOI:** 10.1002/ece3.3100

**Published:** 2017-06-15

**Authors:** Alejandro Ruete, Tomas Pärt, Åke Berg, Jonas Knape

**Affiliations:** ^1^ Department of Ecology Swedish University of Agricultural Sciences Uppsala Sweden; ^2^ Swedish Biodiversity Centre Swedish University of Agricultural Sciences Uppsala Sweden

**Keywords:** citizen‐science data, GBIF, migratory birds, nonsystematic observations, occupancy model, site use, species lists, Sweden, Swedish Species Gateway

## Abstract

Nonsystematically collected, a.k.a. opportunistic, species observations are accumulating at a high rate in biodiversity databases. Occupancy models have arisen as the main tool to reduce effects of limited knowledge about effort in analyses of opportunistic data. These models are generally using long closure periods (e.g., breeding season) for the estimation of probability of detection and occurrence. Here, we use the fact that multiple opportunistic observations in biodiversity databases may be available even within days (e.g., at popular birding localities) to reduce the closure period to 1 day in order to estimate daily occupancies within the breeding season. We use a hierarchical dynamic occupancy model for daily visits to analyze opportunistic observations of 71 species from nine wetlands during 10 years. Our model derives measures of seasonal site use within seasons from estimates of daily occupancy. Comparing results from our “seasonal site use model” to results from a traditional annual occupancy model (using a closure criterion of 2 months or more) showed that our model provides more detailed biologically relevant information. For example, when the aim is to analyze occurrences of breeding species, an annual occupancy model will over‐estimate site use of species with temporary occurrences (e.g., migrants passing by, single itinerary prospecting individuals) as even a single observation during the closure period will be viewed as an occupancy. Alternatively, our model produces estimates of the extent to which sites are actually used. Model validation based on simulated data confirmed that our model is robust to changes and variability in sampling effort and species detectability. We conclude that more information can be gained from opportunistic data with multiple replicates (e.g., several reports per day almost every day) by reducing the time window of the closure criterion to acquire estimates of occupancies within seasons.

## 1 INTRODUCTION

The occupancy of sites by species is a fundamental entity in macroecology, landscape ecology, and metapopulation ecology (Hanski, 1999; Royle & Dorazio, 2008). From a practical perspective, the probability of occurrence of a species is a commonly used measure of habitat suitability (Boyce & McDonald, 1999) and knowing the distribution of a species is basic knowledge needed to make management decisions. Knowledge about the occurrence of species can be gained from systematic surveys where detection/nondetection data of species is recorded (MacKenzie et al., 2006), but also from nonsystematically collected (a.k.a. opportunistic) species observations that are accumulating at a high rate in biodiversity databases (especially for birds; Graham, Ferrier, Huettman, Moritz, & Peterson, 2004). Opportunistic data offer benefits in the form of a wide coverage at spatial and temporal scales (Suarez & Tsutsui, 2004) and often a large number of repeated observations. However, opportunistic data are not collected in a standardized way and there are several potential sources of bias (Lukyanenko, Parsons, & Wiersma, 2016); absences of species are often not available as nondetections are frequently not reported, and corrections for variation in sampling effort are needed (Szabo, Vesk, Baxter, & Possingham, 2010). Other issues include spatial biases (e.g., more reports close to where people live: Fernández & Nakamura, 2015; Mair & Ruete, 2016), trends in recording intensity (Jeppsson, Lindhe, Gärdenfors, & Forslund, 2010; Snäll, Forslund, Jeppsson, Lindhe, & O'Hara, 2014), and differential recording rates among species (Jeppsson et al., 2010; Snäll, Kindvall, Nilsson, & Pärt, 2011) that makes it difficult to compare distribution, occupancy, or abundance patterns among species. These biases have to be considered when analyzing opportunistic data in order to reduce the risk of inferring spurious patterns (Isaac, van Strien, August, de Zeeuw, & Roy, 2014; van Strien, van Swaay, & Termaat, 2013).

Occupancy models are popular in ecology because they enable disentangling the occurrence status from the probability of detection (Kéry, 2010; MacKenzie et al., 2006; Royle & Dorazio, 2008). These models require replicated data on the detection or nondetection of species at multiple sites within a period for which the sites can be assumed closed to colonization and extinction in order to estimate both probability of occupancy and probability of detection (MacKenzie et al., 2006).

Occupancy models were quickly adapted to deal with variation in recording effort in opportunistic citizen‐science data (van Strien et al., 2013). Recently, Isaac et al. (2014) highlighted the usefulness of applying occupancy models to opportunistic data, including measures of effort to partly overcome the problems with several sources of sampling bias. A common approach is to construct absences of species by compiling species lists for individual observers visiting sites. The length of species lists corresponding to observer visits to specific sites is then used as covariates for detection probability, as a proxy for sampling effort and tendency to report species (Isaac et al., 2014; van Strien et al., 2013; Szabo et al., 2010).

So far, in order to gather sufficient replicate visits per sample unit (space and time units) to get robust estimates of occupancy probabilities, ecologist has defined appropriate grid square sizes (e.g., 1 km^2^; van Strien et al., 2013; or 100 km2; Kamp, Oppel, Heldbjerg, Nyegaard, & Donald, 2016) or selected habitat patches (Cruickshank, Ozgul, Zumbach, & Schmidt, 2016) and closure periods, often a breeding season of 2 months or more (Cruickshank et al., 2016; Kamp et al., 2016; Kendall, Hines, Nichols, & Grant, 2013; van Strien et al., 2013). In such an annual occupancy model, occupancy is then defined as the proportion of occupied sites or grid squares at a landscape or regional scale during each season. Some previous studies relaxed the closure assumption by defining the period over which the species is available for detection (Kendall et al., 2013; Roth, Strebel, & Amrhein, 2014), but still assume that the species is always present during a consecutive period within the season and is still restricted to few (e.g., 1‐4) sampling periods within the season. In this way, short‐term dynamics in site use (e.g., as stop‐over for migratory individuals; vagrants) will be oversimplified.

For some taxonomic groups, such as birds, there are often multiple opportunistic observations reported within very short time windows at certain sites. For example, at especially popular birding localities, many different observers visit and report birds within the same day. Using frequent reports to narrow down the length of closure periods in occupancy models of opportunistic observations may enable us to address more detailed questions about within‐season population dynamics, as well as investigating how such dynamics change over time within biologically relevant spatial units holding subpopulations. For example, using a daily closure period, we could estimate the number of days during the season for which a site is being used, which may be more informative than a binary annual occupancy only providing information about whether or not the species was present in a given year. Here, we define “site use” sensu lato, that is, including stop‐overs, daily feeding, etc. in order to keep the term occupancy to be defined for the season or year as has been performed so far in the literature. Then, occupancy can be summarized per season including criteria based on each species biology, and a seasonal site use model could potentially help to disentangle whether the species is using a site as a stop‐over or as a breeding site, and between‐year variation and trends can be estimated for individual sites.

Here, we introduce a seasonal site use model that exploits data‐rich opportunistic citizen‐science databases (e.g., GBIF www.gbif.org; Swedish Species Gateway www.artportalen.se) to narrow down the within‐season closure assumption to within‐day closure. The model is based on a dynamic, daily colonization‐extinction occupancy submodel within each season that copes with known sources of bias in opportunistic data. We use the model to analyze opportunistic reports from citizen‐science data of 71 wetland bird species from nine wetlands collected during 2005‐2014 to estimate species‐specific patterns in site use within and between seasons. Then, we compare patterns of dynamics produced by our seasonal site use model based on daily occupancy estimates to the patterns of dynamics produced by the annual occupancy model with a 3 months closure period. To validate and further test to what extent our model is able to correct for variation in effort and reporting, we simulate data under nine scenarios displaying different patterns in expected levels of occupancy and temporal trends in persistence/colonization rates, number of visits per day and in detection probabilities (see Table 1). With this, we investigate whether model predictions were sensitive to systematic biases in the data.

**Table 1:** Description of the nine simulated datasets (scenarios), each featuring a known combination of patterns in occupancy levels and sampling effort

Scenario	Occupancy Level	Trend in Occupancy	No. of visits	Detection probability
1	High	None	Constant	Observed
2	Medium	None	Constant	Observed
3	Low	None	Constant	Observed
4	Medium	Positive	Constant	Observed
5	Medium	Negative	Constant	Observed
6	Medium	None	Positive trend	Observed
7	Medium	None	Negative trend	Observed
8	Medium	None	Constant	Positive
9	Medium	None	Constant	Negative

## 2 MATERIALS AND METHODS

### 2.1 Observational data for wetland bird species

We obtained a total of 39,384 observations of 71 wetland bird species (Table S1) from nine wetland sites (Table S2 and Fig. S1) in Uppland Province, Sweden, recorded between April 1 and June 30 over the years 2005–2014. Data were obtained via the Swedish Species Gateway (www.artportalen.se), a national gateway for storage of mainly voluntarily reported (opportunistic) biodiversity data. The selected wetland bird species are mainly migratory species that are nesting or foraging in the wetlands (including open waters, reeds, meadows, and areas adjacent to the wetlands) during the investigated time period. The species include swans, ducks, geese, waders, gulls, terns, and passerine birds associated with wetlands and surrounding wet grasslands. Nomenclature follows the dynamic taxonomic database of the organisms of Sweden (http://www.slu.se/dyntaxa). Subspecies were not analyzed, and observations with uncertain species determination were excluded from the analyses.

#### 2.1.1 Nondetection Records

Each observation consisted of a report of a single species, but there was no information about species that were not seen. In order to construct artificial data on nondetections, we first considered each unique observer reporting at least one species at a site on a specific day to constitute a replicate visit within that day, following Kéry, Gardner, and Monnerat (2010) and van Strien, Termaat, Groenendijk, Mensing, and Kéry (2010). Then, for each visit *j*, in day *d*, year *t,* and site *i,* any observation of the focal species was considered as a detection if the species was reported during the visit (*y*_*j,d,t,i*_ = 1) and as a nondetection if it was not reported (*y*_*j,d,t,i*_ = 0). A nondetection then corresponds to the focal species not being reported by an observer reporting at least one other species at the wetland in that day. This procedure was repeated for all study species. Observations were recorded as “missing value” for days and sites without visits (i.e., when no observations were reported from the site in that day).

#### 2.1.2 List length as a proxy for effort

**Figure 1:**
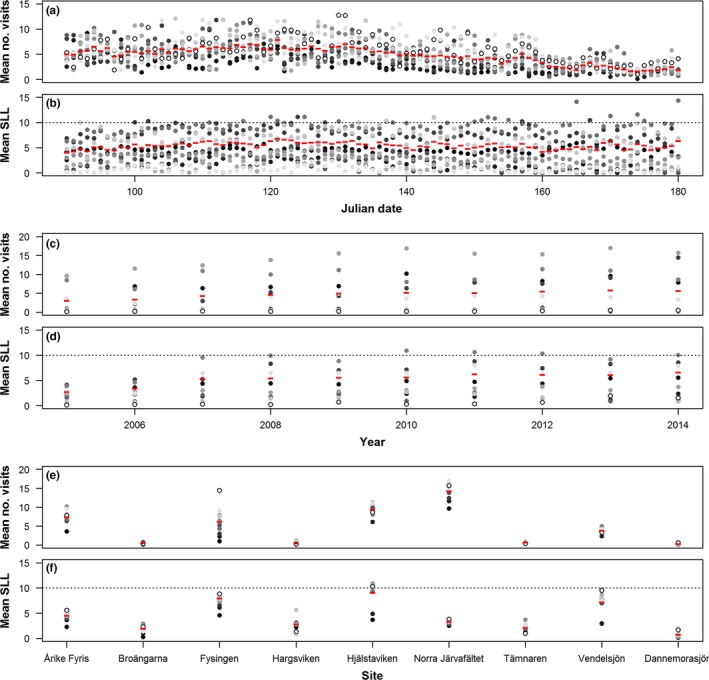
Mean number of visits and mean species list length (SLL) per day (a,b), year (c,d), and site (e,f). Dots show means through years (or sites when years are in the x axis), and the shades of gray differentiate dots by years (or sites; e.g., white dots are year 2005, or site Dannemorasjön). Red dashes show overall means of the data. Dotted lines show the SLL threshold used for long species lists

We calculated the length of the list of observed species for each visit (Species List Length; SLL hereafter), later to be used as a measure of effort (Szabo et al., 2010). For computational reasons, we restricted the maximum number of visits to 40 per day and site, prioritizing visits with the longest species lists. SLLs ranged from 1 to 45 species. Around 60% of all visits consisted of single observations (SLL = 1), although this proportion decreased over time (Fig. 1). In Fig. S3, we compare the results of the model using the full dataset and only visits with long species lists (SLL ≥ 10).

### 2.2 Seasonal site use model: Daily site occupancies using daily‐based replicated observations

For each species, we use a dynamic state‐space occupancy model (MacKenzie et al., 2006; van Strien et al., 2013) to estimate daily occurrence status, adjusted for detection and reporting probability (hereafter simply called detection probability). The occupancy model consists of two submodels coupled hierarchically: a process model (for the daily occurrence status) and an observation model (for the stochasticity of species detections); the latter being conditional on the process submodel. In this way, each observation y_*j,d,t,i*_ is modeled as.

(1) $$y_{j,d,t,i}∼\text{Bernoulli}\left(u_{d,t,i}\times p_{j,d,t,i}\right)$$

where *u*_*d,t,i*_ is the (binary) occurrence status of the species in day *d*, year *t,* and site *i*, and *p*_*j,d,t,i*_ is the detection probability of the species in each visit *j*, given that the species is present. The occurrence status *u* depends on the occurrence probability ψ per day *d*, year *t,* and site *i* recursively through:

(2) $$u_{d,t,i}∼\text{Bernoulli}\left(\psi_{d,t,i}\right),$$

(3) $$\psi_{d,t,i}=u_{d-1,t,i}\times \varphi_{d-1,t,i}+\left(1-u_{d-1,t,i}\right)\times \gamma_{d-1,t,i},$$

Thus, whether site *i* that is occupied in day *d*−1 is still occupied in day *d* is determined by the persistence probability (φ), whereas whether site *i* that is unoccupied in day *d*−1 is occupied in day *d* depends on the colonization probability (γ). Because we expect persistence and colonization probabilities to vary along the season, we further modeled these parameters as

(4) $$\begin{array}{cc} \text{probit}\left(\varphi_{d-1,t,i}\right)= & \text{pCoef1}+\text{pCoef2}\times {\text{JDay}}_{d-1}+\text{pCoef3}\times {\text{JDay}}_{d-1}^{2}\\ & +\epsilon {pI}_{i}+\epsilon pT_{t}, \end{array}$$

(5) $$\begin{array}{cc} \text{probit}\left(\gamma_{d-1,t,i}\right)= & \text{gCoef1}+\text{gCoef2}\times {\text{JDay}}_{d-1}+\text{gCoef3}\times {\text{JDay}}_{d-1}^{2}\\ & +\epsilon {gI}_{i}+\epsilon gT_{t}, \end{array}$$

where JDay is the Julian date. We modeled the effect of the Julian date as a quadratic function to allow the colonization and persistence parameters to increase, decrease, or both within the season. In this way, the model may be suitable for a wider range of species with different phenology. We also added random effects for site (ε*pI* and εg*I)* and year (ε*pT* and εg*T*) (see Appendix S1 for commented scripts).

The annual average use of site *i* by the focal species can be defined from the derived quantity $z_{t,i}=\left(\sum_{d=1}^{n}u_{d,t,i}\right)/n$ where *n* is the number of days during the season. In the same way, a regional annual site use (*Z*_*t*_) can be defined as the average number of occurrence across all days and sites.

The observation submodel contains a detection probability *p* per visit *j*. Because we expected detection to vary between visits, we modeled it as a saturation function of each visit's SLL,

(6) $$p_{j,d,t,i}=1-\delta_{t,i}/\left({\text{SLL}}_{j,d,t,i}+\delta_{t,i}\right),$$

where δ_*t,i*_ is a real positive number defining the SLL required to obtain a detection probability equal to 0.5 for a visit. Consequently, the shorter the list, the lower the assumed observation effort or the likelihood to report an observed species (van Strien et al., 2013; Szabo et al., 2010). With this function, *p*_*j,d,t,i*_ converges asymptotically to 1 as SLL_*j,d,t,i*_ gets closer to ∞; however, note that *p*_*j,d,t,i*_ will be lower than 1 even when SLL is equal to the local species richness. We further modeled δ_*t,i*_ as

(7) $$\text{log}\left(\delta_{t,i}\right)={\text{dCoef1}}_{i}+\text{dCoef2}\times {\text{PLL}}_{t},$$

where dCoef1 is a site‐specific parameter accounting for detectability varying among sites. The variable PLL_*t*_ is the proportion of long species lists (≥10 of the study species) over the total number of lists each year among the nine sites (Fig. S2) and serves as a proxy to account for potential changes in reporting behavior among observers over time. Preliminary results showed that this model cannot estimate variability in probability of detection as a function of Julian date because it interfered with the estimation of the persistence and colonization parameters in the occurrence submodel. Therefore, detectability is assumed to be constant within the season (see the 4 section for pros and cons of this model feature).

### 2.3 Annual site‐occupancy model using within‐season replication of observations

We also fitted a dynamic occupancy model to estimate annual occupancy probability (i.e., using a closure period of 90 days; see, e.g., van Strien et al., 2013), in order to directly compare our results to previous methods adopted for opportunistic data. Given the abundance of replicated visits, we only used visits with SLL ≥ 10.

All models were fitted within the Bayesian framework using JAGS (Appendix S1; Plummer, 2012). We chose conventional vague priors for all parameters, using Normal distributions centered at zero and with standard deviation (*SD*) 1,000 for effect parameters. We assumed random effects to follow a normal distribution centered at zero with independent standard variation defined as σ = (1/τ)^1/2^, where τ is a precision parameter following a Gamma distribution with shape and scale parameters equal 0.001. We used sufficient MCMC iterations to achieve convergence of the models (burn‐in = 5,000, update = 15,000). We used 95% quantiles as credible intervals to describe the precision of parameter estimates (Kéry, 2010).

### 2.4 Goodness of fit through prediction

To investigate goodness of fit, we checked if the model was able to reconstruct the original data given the estimated parameter values (Chambert, Rotella, & Higgs, 2014; Gelman & Hill, 2007; Kéry, 2010). To do so, we replicated observation events of a species given its estimated daily occupancy status, and the effort spent in each visit (i.e., data replicated from the posterior distributions). We summarized daily observations (both observed and replicated data) into mean observed annual site use by keeping the maximum detection status among the daily visits (1 if detected at least once during the day, 0 otherwise) and averaging these values across the seasons (90 days) at each site. We then graphically compared observed and replicated data of mean annual site use on a 1:1 discrepancy plot for all sites together.

We also evaluated goodness of fit of the models using site‐specific Bayesian *p*‐values, a.k.a. “posterior predictive checks” (Chambert et al., 2014; Kéry, 2010). Bayesian *p*‐values quantify the probability that the lack of fit of data replicated under the fitted model is smaller than the lack of fit of the observed data. *p‐*values close to .5 indicate the model fits the data adequately and values close to 0 or to 1 indicate under‐ or overfitting (Kéry, 2010). The measure of discrepancy chosen in this case is the sums of squares of Pearson's residuals (Kéry et al., 2010; SSQ; eqn 8) between observed mean annual site use ($w_{t,i}=\left(\sum_{d=1}^{n}\max_{j}y_{j,d,t,i}\right)/n$; and *w.new*_*t,i*_ for replicated data) and the model prediction of observed mean annual site use (i.e., the average of the daily probabilities of detecting the species at least once if present; ${\bar{w}}_{t,i}=\left(\sum_{d=1}^{n}u_{d,t,i}\times \left({1-\prod }_{j}\left(1-p_{j,d,t,i}\right)\right)\right)/n$, as follows:

$${\text{SSQ}}_{i}^{\mathrm{obs}}=\sum_{t}\left(w_{t,i}-{\bar{w}}_{t,i}\right)/\sqrt{{\bar{w}}_{t,i}*{\left(1-p_{t,i}\right)}^{2}};$$

(8) $${\text{SSQ}}_{i}^{\mathrm{new}}=\sum_{t}\left(w\;.\;{\text{new}}_{t,i}-{\bar{w}}_{t,i}\right)/\sqrt{{\bar{w}}_{t,i}*{\left(1-p_{t,i}\right)}^{2}}.$$

### 2.5 Validation through simulations

We tested the assumptions and performance of our model under different scenarios by fitting it to simulated data with known occurrence and sampling patterns. We simulated data using the same sampling structure as for the real data, that is, daily replicates of visits during ten 90‐day seasons at five sites, and using the observed increasing proportion of long lists through time (PLL_*t*_, Fig. S2). The number of visits per day was drawn from a Poisson distribution constrained to [1, 50] and with a mean varying over time and with additional among site variability (see Appendix S2). The length of each visits` species lists was randomly drawn according to the observed proportion of single, short, and long species lists (see Appendix S2 for more details). That is, there is at least one visit and no more than 50 visits at each day and site. We fitted the model to nine simulated datasets, each representing a different scenario with patterns in occupancy level and effort that are likely present in opportunistic data and may influence model performance, but which are not explicitly accounted for in the model (Table 1):

1. high, medium, or low overall occupancy levels with variability among lakes in all other parameters but stable occupancy through time;
2. positive or negative trends over time on the persistence and colonization rates
3. increasing or decreasing number of visits over time (maintaining the variability in effort among sites)
4. positive or negative trends in detection (and reporting) probabilities, on top of the observed trend in PLL_*t*_ that is common to all scenarios.

For more details about the simulation procedure and parameters settings, read Appendix S2. We evaluated the goodness of fit of the models in the same way as described above, and the ability of the models to estimate the known occurrence data.

## 3 RESULTS

### 3.1 Analyses with real data on wetland birds

**Figure 2:**
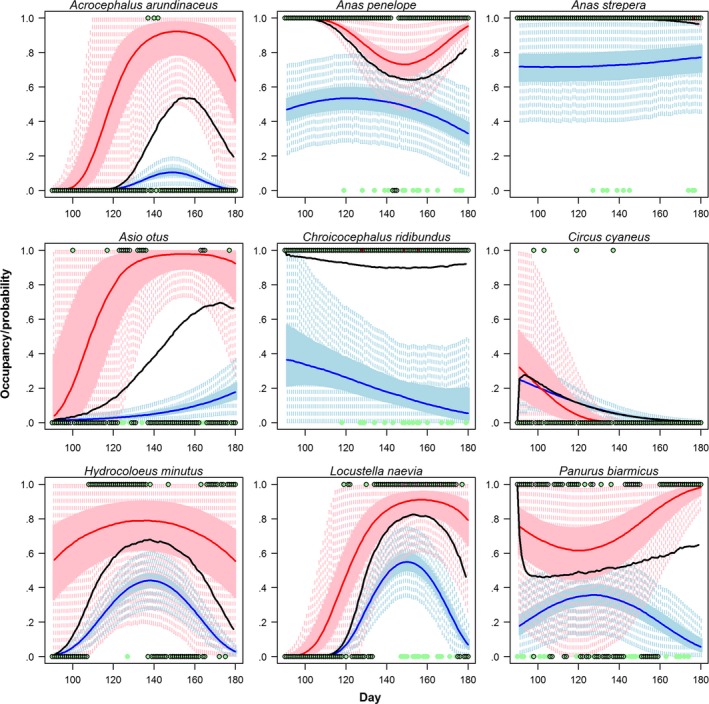
Observed and estimated daily occupancy of nine species (filled green and empty circles, respectively). The black lines show the daily mean occupancy probability. Red and blue lines show the mean daily persistence and colonization probabilities, respectively (shades show the 50% and 95% CI). Example data from site Hjälstaviken in 2014

**Figure 3:**
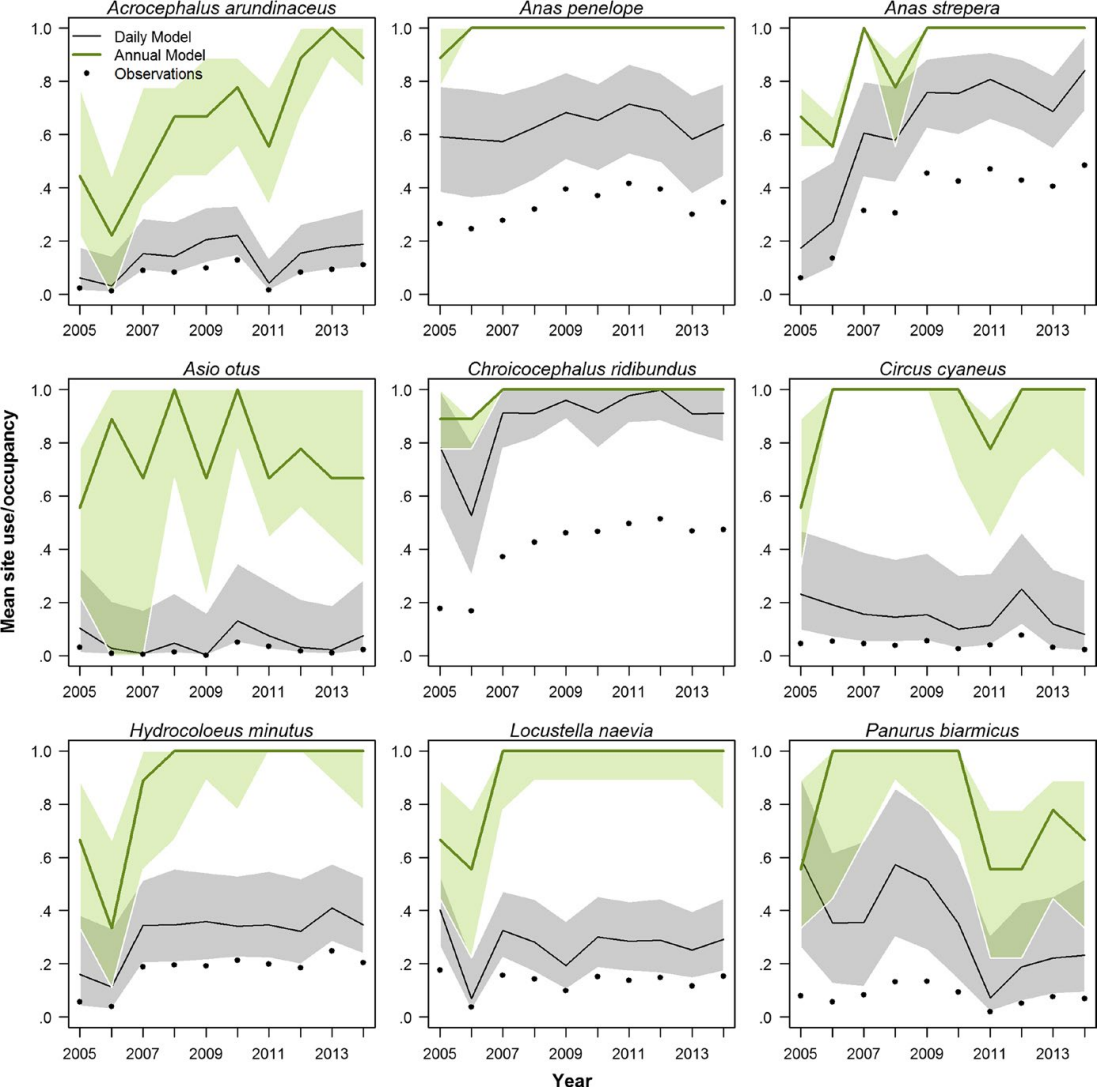
Estimated annual occupancy (green) and seasonal site use (black) over the study region (nine sites) for nine selected wetland bird species. Solid lines and shaded areas show the median and 95% CI around the estimated occupancy and mean site use, respectively. Black dots indicate observed mean site use

**Figure 4:**
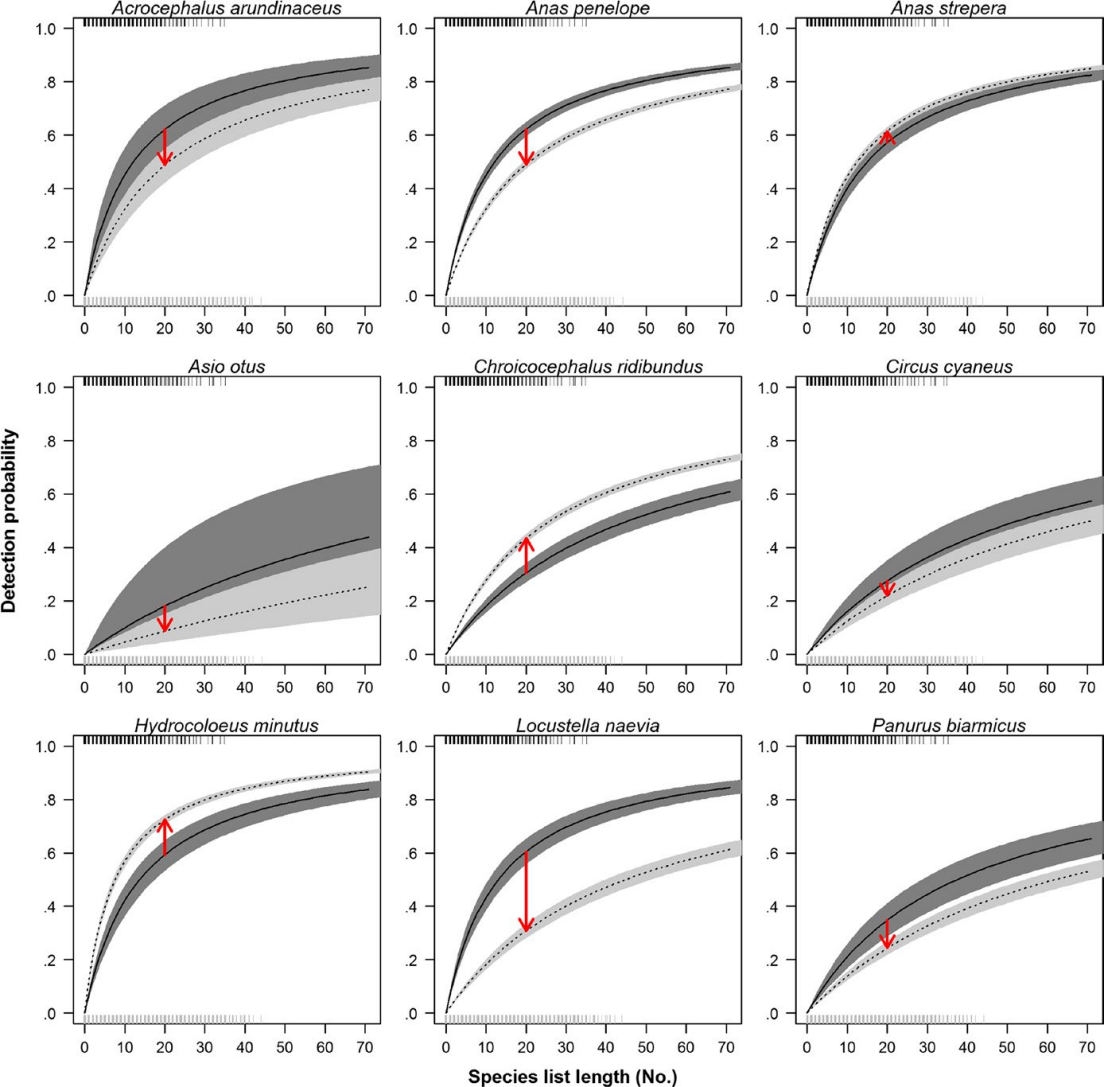
Detection probability for nine selected species, as a function of species list length (SLL), for 2005 (solid lines, dark shades) and 2014 (dashed lines, light shades). The arrows indicate the direction of the change in detection through time (i.e., the effect of PLL_*t*_). On each plot, short lines on the top and bottom axis indicate the visits’ SLL for 2005 (black lines on the top) and 2014 (gray lines on the bottom)

The model estimates daily occupancy by correcting for false absences based on each day's effort (both number of visits and each visit's SLL) and on the assumed species colonization/extinction dynamics at a site and year (Fig. 2). Estimated mean annual site use (summarized from estimated daily occupancy) varies from year to year, displaying large between‐year changes for some species (Fig. 3, exemplified with nine selected bird species). Estimates of occupancy probability were in general precise (i.e., small credible intervals) even for rare species, as long as some of the sites were well sampled (i.e., enough to confidently separate occupancy and detection probabilities) and if the species occurred regularly at those sites (i.e., consistently during the same periods across all years it was present; e.g., Asio otus). The probability of detection depended on the visits’ SLL and on the proportion of long lists, PLL_*t*_. Estimates of the probability of detection were less precise for species with lower site use (Fig. 4). We observed low discrepancy between observations and predicted observations of mean annual site use, and no systematic bias was observed for 64 of 71 investigated species (Appendix S3). However, deviations from the 1:1 line between observations and expectations (to either side) were noted for seven species with anecdotic occurrences in some sites. Bayesian *p*‐values (posterior predictive checks) were useful to corroborate if the observed local daily dynamics adjust to the overall daily dynamics estimated from all sites. Bad fit was then only observed on individual sites with little data were the local dynamics did not match the dynamics observed in other sites (Appendix S3).

### 3.2 Comparing patterns of dynamics from the seasonal site use vs. the annual occupancy model

Using the visits with long species list, we calculated the corresponding annual occupancy over the nine wetlands between 2005 and 2014. Although annual occupancy levels are generally higher than the mean site use, they frequently display a similar broad pattern of temporal dynamics (Fig. 3). However, for some more common or widespread species, the annual occupancy model often displayed no temporal variation in occupancy, as all sites were determined occupied in all years (e.g., *A. penelope* and *C. cyaneus*, and *C. cygnus* and *H. minutus* after 2008, Fig. 3). By contrast, for some of these species, the site use model suggested a positive trend (*C. cygnus*) or a possible negative trend (*C. cyaneus*) in site use. Similarly, large differences between annual occupancy and mean site use as estimated by the annual vs. daily occupancy models, respectively, show that the annual model fails to handle the effects of temporary visits by overestimating the species annual occupancy during the breeding season (e.g., see 4).

### 3.3 Validation of the seasonal site use model by simulated data

**Figure 5:**
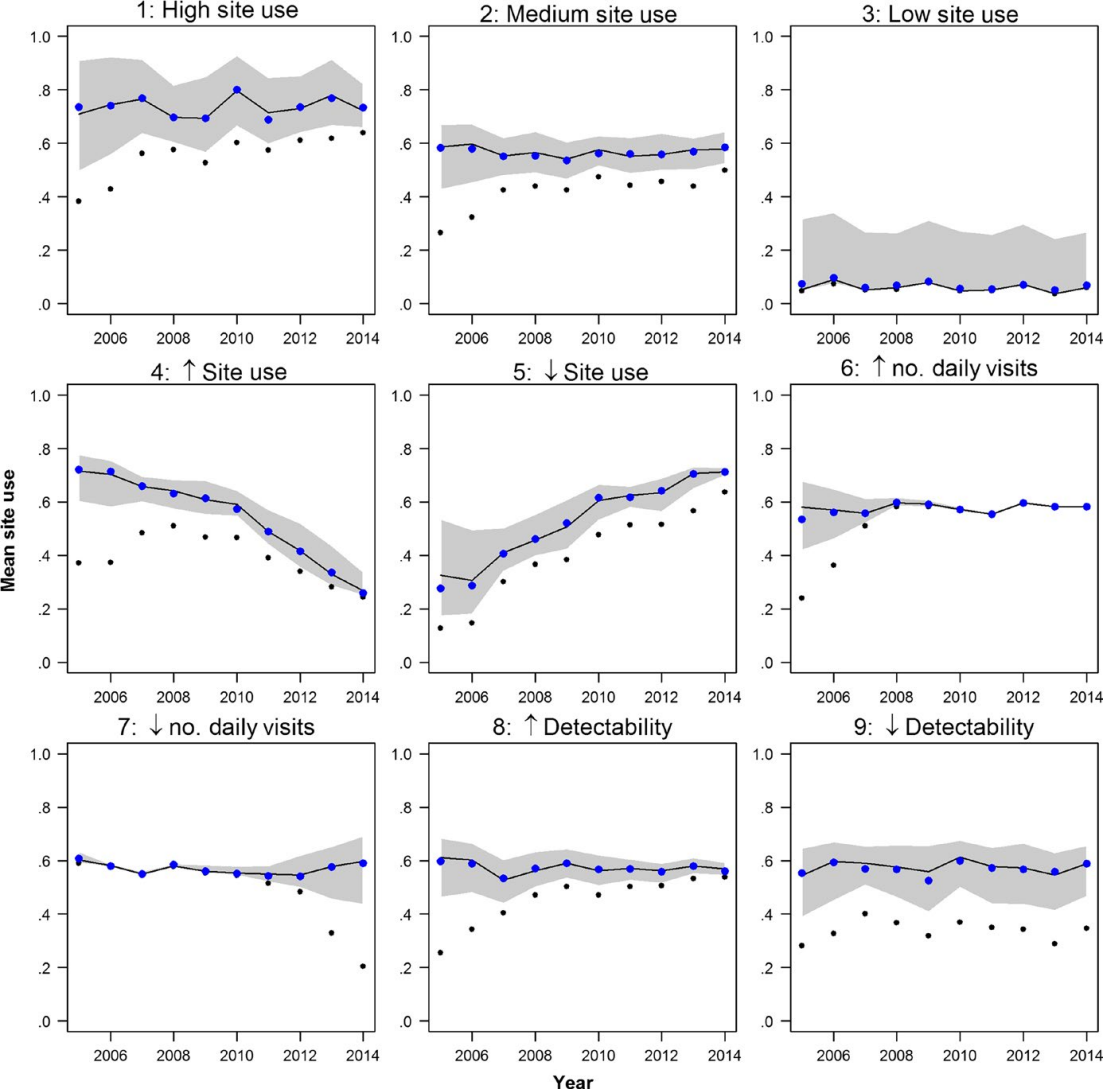
Estimated (black line) and simulated (blue dots) seasonal site use in the study region (nine sites) over time for nine scenarios of simulated datasets (Table 1), each featuring a known combination of patterns in occupancy levels and sampling effort. Black lines and shaded areas show the median and 95% CI around the estimated mean site use. Black dots indicate observed mean site use

The daily occupancy model gave accurate and robust estimates of annual site use for the simulated data regardless of the mean site use level (i.e., number of days present in any site in the region; Scenarios 1, 2s and 3), and trends in occupancy (Scenarios 4 and 5), number of visits (Scenarios 6 and 7) or in detectability (Scenarios 8 and 9). Most of the simulated yearly site use data points were overlapping with the site use values estimated from the model (i.e., all simulated points were within the 95% CI, but mostly close to the median of the estimates) across all scenarios (Fig. 5).

The model uncertainty (95% CI), however, depends on the combined effect of number of daily visits and each visit's SLL, but also on the mean site use level (i.e., number of days present at the site).When mean site use level is very low (Scenario 3), there are too few detections to inform the model, which becomes less accurate and less precise at estimating the probabilities of detection and the colonization/extinction probabilities (Fig. 5, Scenario 3). This results in high uncertainties unless the sampling effort is high enough to detect every presence of the species.

The model estimated temporal trends in site use regardless of trends in number of observations per day (Scenarios 4 and 5). As expected, model uncertainty is higher the lower the number of visits per day (Scenarios 6 and 7) and the lower the species detectability (Scenarios 8 and 9). Regardless of the probability of detection, the higher the number of visits per day, the more likely the species is detected if present. Therefore, the higher the number of visits per day, the smaller the discrepancy between observations and the occupancy status of the species (Scenario 6). Alternatively, even accounting for an increase in PLL_*t*_ in all visits, detections are not guaranteed if the number of visits is too few (Scenario 7). Despite an increase in model uncertainty, the model correctly estimated the occurrence status of the species under both changing number of visits and changing species detectability.

**Figure 6:**
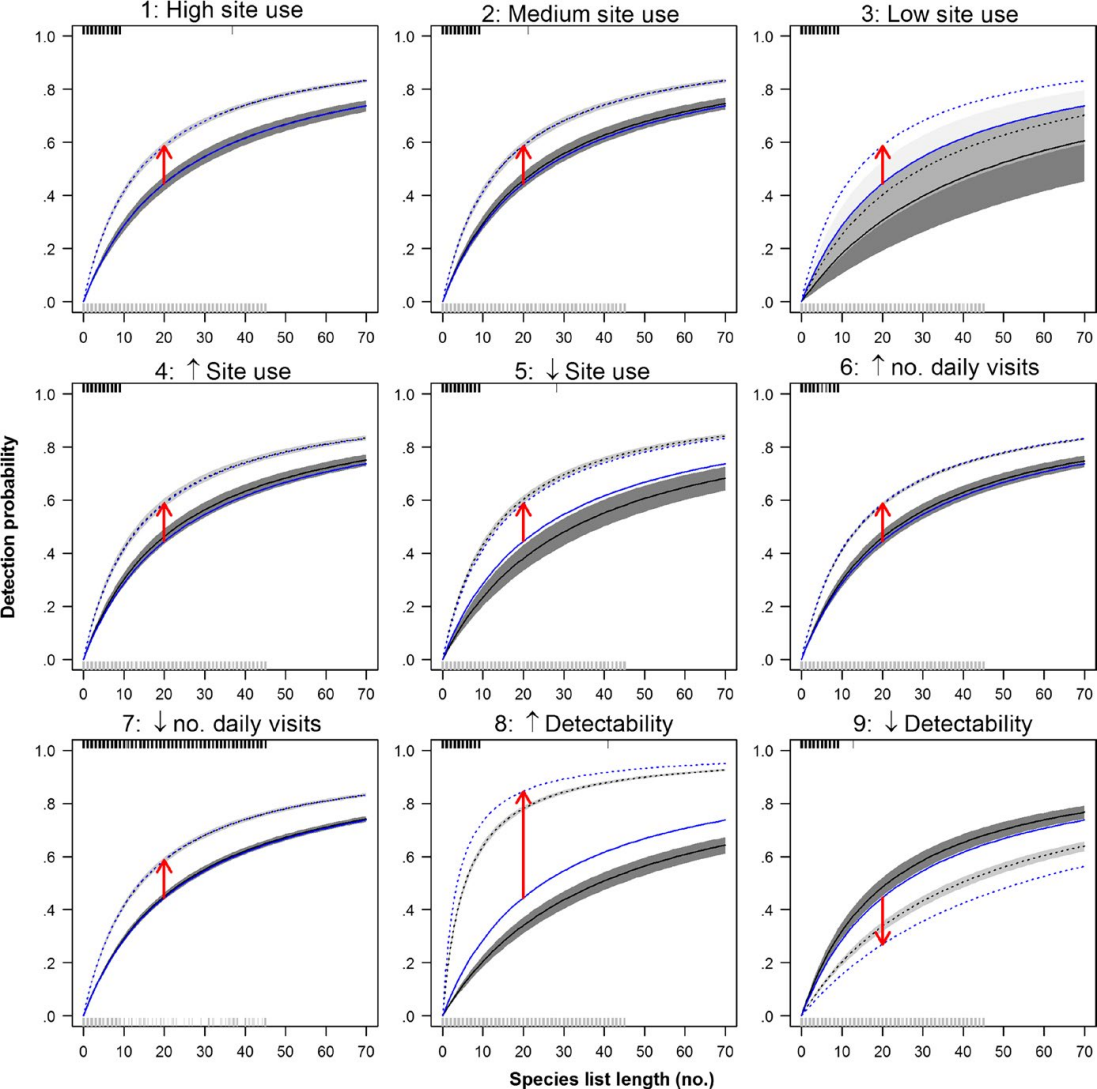
Detection probability as a function of species list length. Known (blue line) and estimated (black lines and shades) functions are shown for year 1 (solid lines) and year 10 (dashed line). The arrows indicate the direction of the change in detection through time (i.e., the effect of PLL_*t*_). Short vertical lines (ticks) on the top and bottom axis indicate the distribution of SLL for 2005 (black lines on the top) and 2014 (gray lines on the bottom)

The model identified changes in detectability independently of the trends in number of visits and PLL_t_. Despite that the observed increase in proportion of long lists (PLL_*t*_, Fig. 1 and Fig. S2) is included in the model as a time‐dependent variable affecting the probability of detection, the model also adjusts the effect parameter for PLL_*t*_ to nonobserved changes in detectability (Scenarios 8 and 9, Figs. 5 and red arrows in Fig. 6). That is, even when the proportion of long lists among visits is high (high PLL_*t*_), detectability can naturally decrease due to, for example, change in habitat conditions. However, for the simulated data, the model is able to correct for this trend and estimates of occupancy are not affected.

## 4 DISCUSSION

### 4.1 Seasonal site use vs. annual occupancy models

Previous annual occupancy models have typically used the breeding season (e.g., 2–3 months) as the time window to estimate annual occupancy at sites (e.g., 1 km^2^ grid squares) (Isaac et al., 2014; Royle & Dorazio, 2008; Royle & Kéry, 2007; van Strien et al., 2013). However, using such a long time window may likely violate the assumption of closure for mobile species with within‐season dynamics, thus potentially reducing estimates of probability of detection and increase the uncertainty of occupancy estimates (MacKenzie, Nichols, Hines, Knutson, & Franklin, 2003). For example, when the aim is to analyze occurrences of breeding species, an annual occupancy model will overestimate site use of species with temporary occurrences (e.g., migrants passing by, single itinerary prospecting individuals) as even a single observation during the closure period will be viewed as an occupancy. On the other hand, an occupancy model with within‐season dynamics, such as our seasonal site use model, will produce estimates of the extent to which sites are actually used. Two illustrative examples are the little gull (*H. minutus*) and the hen harrier (*Circus cyaneus*), which we know from careful observations made by the local ornithological society, attempted to breed in only three and none of the nine wetlands, respectively, during 2005 to 2014 (Annual birds reports from the Ornithological Society of Uppland 2005–2014). These species regularly stop‐over at these wetlands on their way to their breeding areas in northern Sweden and Finland, being frequently observed for several days during spring and early summer. The annual model therefore suggests an occupancy probability close to one for most years for this species (Fig. 3). The seasonal site use model, on the other hand, suggests a relatively low site use. In this way, the site use model may be used to detect these passages of migrants thus enabling a separation between potential breeders and migrants or vagrants (Fig. 3 and Appendix S3). Furthermore, as individuals may move in and out of the sites during the study period, daily occupancy of a site may indicate how site use is changing during the season. In this way, such a seasonal site use model may also be able to estimate the relative importance of different sublocalities as foraging or stop‐over sites in a network of, for example, wetland sites.

Opportunistic data at frequently visited sites offer good opportunities to narrow the time window of the closure period because of the large amount of data at specific sites. Several of the localities in our study, which include popular birding wetlands with observation towers, were visited two or more times per day by different observers during the spring 2005–2014. In general, the span of the within‐season closure period of our model may be optimized to the data at hand. If, for instance, multiple visits to sites are common on a weekly but not on a daily basis, a closure period of 1 week may be used instead.

### 4.2 Opportunistic data and the robustness of the seasonal site use model

The probability of at least one reported observation of a species at a site on a particular day is the result of both the probability of detection of each visit and the number of visits made. The probability of detection during each visit depends on effort allocated to observing species and the willingness to report them if seen. SLL is an established surrogate for the effort of a visit in opportunistic data (Barnes, Szabo, Morris, & Possingham, 2015; van Strien et al., 2013; Szabo et al., 2010). Even though detection probability and willingness to report an observation differ largely among species, it is expected that the longer the SLL, the lower the chance of deliberately leaving species out of the report (van Strien et al., 2013). However, even “low‐quality” observations (e.g., SLL = 1) may be informative for the occupancy status of the few species that are on such a list. If there are sufficient visits reporting only one or a few species, they can be useful for estimating occupancy (e.g., beginning of Scenario 7, where plenty of visits each with very short species lists are enough to precisely estimate the mean site use). Therefore, as an alternative to the seminal species list comparison approach proposed by Szabo et al. (2010) where short species list were omitted, we also make use of even single (incidental) observations that have often been regarded as containing little information (Isaac & Pocock, 2015; Szabo et al., 2010). This addition does not add noise but rather improves precision in estimates of daily occupancy and mean site use of rare species (Fig. S3).

In our site use model, detectability is assumed to be site and year specific but constant within the season. This is because trying to estimate daily variations in detectability interfered with the estimation of the daily persistence and colonization parameters in the occurrence submodel. However, because the probability of detection is determined by each visit's SLL that varies among visits and may decrease along the season (Strebel, Kéry, Schaub, & Schmid, 2014), the model implicitly allows for some variation in the probability of detection within the season. Alternatively, in case there are good reasons to believe that detectability changes during the breeding season (e.g., due to increased cryptic behavior), a change in detectability between intermediate time windows (e.g., months) could be parameterized and tested with this model by adding a time covariate to Equation (7) (see 2).

The seasonal site use model presented here accounts for effects of changes in the behavior of observers over time on species detectability, using the overall proportion of species lists longer than 10 (PLL_*t*_) as a proxy. Specifically, PLL_*t*_ captures a nonlinear increase in the proportion of visits with long lists during the first few years in the data analyzed here, suggesting that the overall quality of reports may have increased. The effect of PLL_*t*_ was, however, negative for some species (red arrows in Fig. 4) indicating that observers are decreasingly reporting certain species. This may suggest a negative trend in the species abundance that is not reflected in the species occupancy. Alternative proxies, such as temporal trends, could also be used to adjust for changes in reporting behavior over time, although when tested in this study, the MCMC sampling algorithms did not find a solution for the model (i.e., the MCMC sampling chains did not converge into a high probability area of the parameter space).

In addition to the assumption that species list length serves as a reasonable proxy for sampling effort, site‐occupancy models of opportunistic data rely on additional assumptions. For example, a general assumption of site‐occupancy models is that reports from different visits are independent, which may not be the case if observers share their sightings. Despite estimates of site use being robust to the deviations explored in the simulated scenarios, there is thus no guarantee that the model correctly adjusts for variation in effort, observer behavior, and observer willingness to report a species. Unfortunately, no further conclusion can be drawn without validation against systematically collected data. Currently, little is known about variations in observer behaviors and the decisions underlying whether observations are reported or not. Some studies comparing analyses of opportunistic data against survey data do suggest that occupancy models may handle the most serious causes of bias (Isaac et al., 2014; van Strien et al., 2013), while other studies suggest a poor fit between opportunistic and survey data (Kamp et al., 2016).

In conclusion, by making use of dense opportunistic data at popular localities, we markedly reduce the time interval for the closure criterion (here to 1 day periods) and get repeated estimates of occupancy within a predefined time period (here the breeding season of 3 months) to estimate: (1) daily site occupancy and (2) site use during the breeding season (here mean number of days a species is present at a site) in contrast to a binary variable produced by an annual occupancy model, and hence (3) the possibility to redefine the criteria for counting a species as present at a site based on its activity within the season. Model validation based on simulated data suggests that the performance of the seasonal site use model in terms of capturing the species mean site use over time is robust to underlying variability and trends in effort and species detectability. Furthermore, the seasonal site use model has the potential to estimate the relative importance of each site in a wetland network in terms of site use.

## CONFLICT OF INTEREST

None declared.

## DATA ACCESSIBILITY

All data, the workflow to download and collate the data, and all the R and Jags scripts to conduct the complete workflow are hosted in the GitHub repository https://github.com/aleruete/Daily-Occupancy-Site-Use-Model (https://doi.org/10.5281/zenodo.571063). The raw data can also be obtained from www.analysisportal.se at any time.
