# Supplementary material for: Exploiting opportunistic observations to estimate changes in seasonal site use: An example with wetland birds
Source: Ecol Evol. 2017 Jun 15;7(15):5632–44. doi: 10.1002/ece3.3100 (PMC5551100; doi:10.1002/ece3.3100)
Supplement: Supplementary file 1 [file ECE3-7-5632-s001.docx]

*Supporting Information for*

**Exploiting daily opportunistic observations to estimate changes in seasonal site use – an example with wetland birds**

Alejandro Ruete, Tomas Pärt, Åke Berg, Jonas Knape

**Table S1.** Lists of the study species

| Scientific Name |
| --- |
| *Acrocephalus arundinaceus* |
| *Acrocephalus dumetorum* |
| *Acrocephalus palustris* |
| *Acrocephalus schoenobaenus* |
| *Acrocephalus scirpaceus* |
| *Anas acuta* |
| *Anas clypeata* |
| *Anas crecca* |
| *Anas penelope* |
| *Anas platyrhynchos* |
| *Anas querquedula* |
| *Anas strepera* |
| *Anser anser* |
| *Anthus pratensis* |
| *Ardea cinerea* |
| *Asio flammeus* |
| *Asio otus* |
| *Aythya ferina* |
| *Aythya fuligula* |
| *Botaurus stellaris* |
| *Branta canadensis* |
| *Branta leucopsis* |
| *Bucephala clangula* |
| *Calidris alpina* |
| *Calidris pugnax* |
| *Carpodacus erythrinus* |
| *Charadrius dubius* |
| *Charadrius hiaticula* |
| *Chlidonias niger* |
| *Chroicocephalus ridibundus* |
| *Circus aeruginosus* |
| *Circus cyaneus* |
| *Circus pygargus* |
| *Crex crex* |
| *Cygnus cygnus* |
| *Cygnus olor* |
| *Emberiza schoeniclus* |
| *Fulica atra* |
| *Gallinago gallinago* |
| *Gallinula chloropus* |
| *Grus grus* |
| *Haematopus ostralegus* |
| *Haliaeetus albicilla* |
| *Hydrocoloeus minutus* |
| *Larus argentatus* |
| *Larus canus* |
| *Larus fuscus* |
| *Larus marinus* |
| *Limosa limosa* |
| *Locustella fluviatilis* |
| *Locustella luscinioides* |
| *Locustella naevia* |
| *Luscinia luscinia* |
| *Mergus merganser* |
| *Motacilla flava* |
| *Numenius arquata* |
| *Pandion haliaetus* |
| *Panurus biarmicus* |
| *Phalacrocorax carbo* |
| *Podiceps auritus* |
| *Podiceps cristatus* |
| *Podiceps grisegena* |
| *Porzana porzana* |
| *Rallus aquaticus* |
| *Saxicola rubetra* |
| *Sterna hirundo* |
| *Sylvia communis* |
| *Tachybaptus ruficollis* |
| *Tadorna tadorna* |
| *Tringa totanus* |
| *Vanellus vanellus* |

**Table S2**. Wetland sites

| Site | Area (ha) | Perimeter (Km) |
| --- | --- | --- |
| Årike Fyris | 593.2 | 12.4 |
| Broängarna | 184.1 | 6.1 |
| Dannemorasjön | 1609.1 | 18.0 |
| Fysingen | 1412.6 | 15.7 |
| Hargsviken | 77.4 | 4.4 |
| Hjälstaviken | 440.5 | 8.1 |
| Norra Järvafältet | 3088.4 | 21.8 |
| Tämnaren | 9181.3 | 36.9 |
| Vendelsjön | 1957.5 | 24.6 |


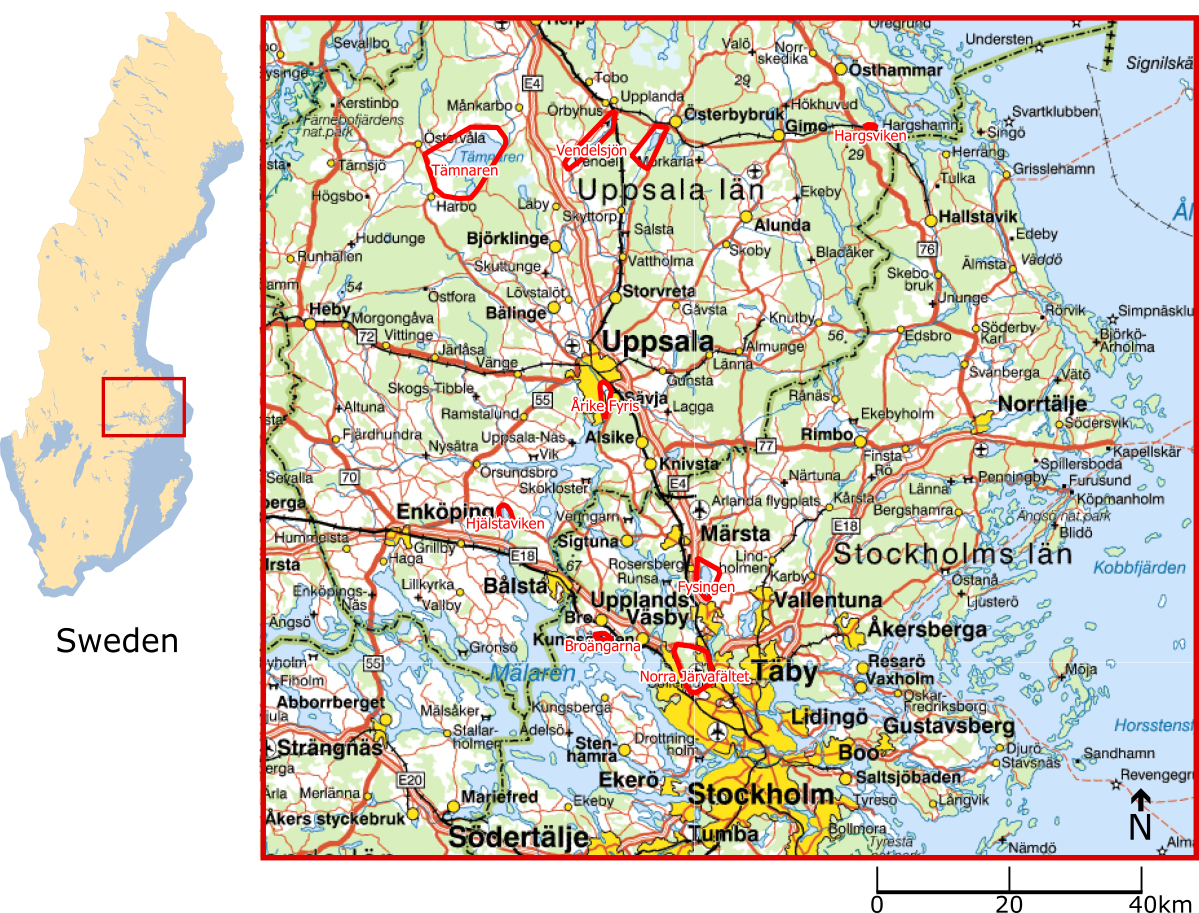


**Figure S1**. Location and extent of study sites in Sweden (marked in red).


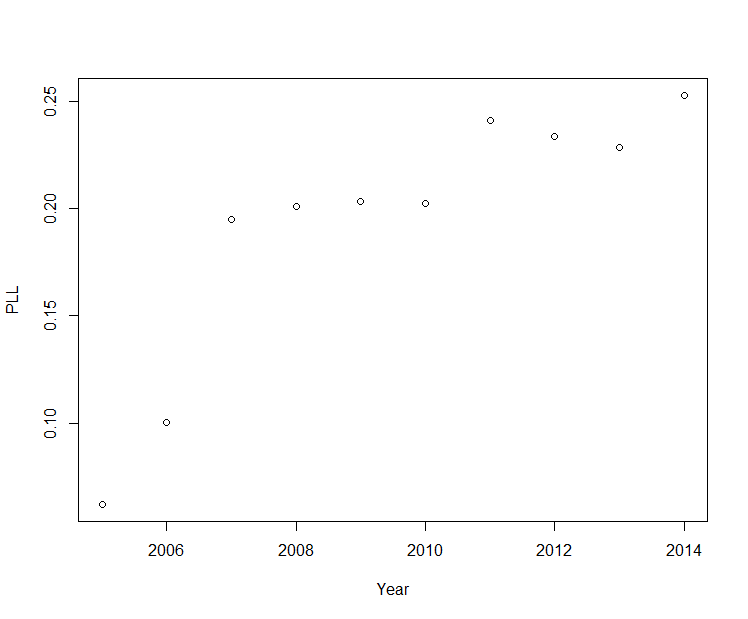


**Figure S2.** PLL, i.e. the proportion of long species lists (SLL ≥ 10) over the total number of species lists (i.e. visits) over the sampled years.


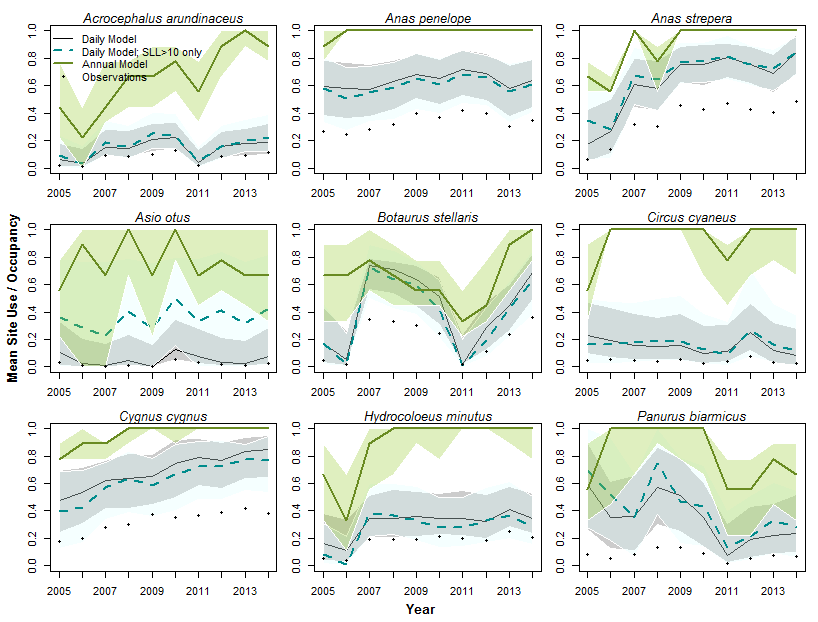


**Figure S3**. Estimated annual occupancy (green) and mean site use (dashed cyan) using the only long species lists (SLL ≥ 10; cyan) and mean site use using the full dataset (black), over the study region (nine sites) for nine selected wetland bird species. Lines and shaded areas show the median and 95% CI around the modelled occupancy and mean site use, respectively. Black dots indicate observed mean site use.

**Appendix S1**. JAGS Script

## Observation Model

for(i in 1:nsite){

for(t in 1:nyear){

for(d in 1:nday){

for(j in 1:nrep[d,t,i]){ # nrep = number of visits

y[j,d,t,i] ~ dbern(Py[j,d,t,i])

Py[j,d,t,i] <- u[d,t,i] * p[j,d,t,i]

p[j,d,t,i] <- 1- delta[t,i] / (SLL[j,d,t,i] + delta[t,i])

# SLL = Species List Length, delta = Half-saturation parameter

} # end nrep

} # end nday

## Detectability coefficients

log(delta[t,i]) <- dCoef1[i] + dCoef2 * PLL[t]

} # end nyear

} # end nsite

## Process Model

for(i in 1:nsite){

for(t in 1:nyear){

u[1,t,i] ~ dbern(psiD[i])

for(d in 2:nday){

u[d,t,i] ~ dbern(muU[d,t,i])

muU[d,t,i] <- u[d-1,t,i] * phi[d-1,t,i]

+ (1 - u[d-1,t,i]) * gamma[d-1,t,i]

## Persistence Probability

probit(phi[d-1,t,i]) <- pCoef[1] + pCoef[2] * days[d-1]

+ pCoef[3] * days[d-1] * days[d-1]

+ eta.p[i] + eta.pT[t]

## Colonization Probability

probit(gamma[d-1,t,i]) <- gCoef[1] + gCoef[2] * days[d-1]

+ gCoef[3] * days[d-1] * days[d-1]

+ eta.g[i] + eta.gT[t]

} # end nday

} # end nyear

} # end nsite

#########

## Priors

for(i in 1:nsite){

psiD[i] ~ dunif(0,1)

dCoef1[i] ~ dnorm(0, 0.001)

eta.p[i] ~ dnorm(0, p.tau)

eta.g[i] ~ dnorm(0, g.tau)

}

for(t in 1:nyear){

eta.pT[t] ~ dnorm(0, pt.tau)

eta.gT[t] ~ dnorm(0, gt.tau)

}

dCoef2~dnorm(0, 0.001)

pCoef[1:3] ~ dmnorm(pC0[], PC0[,])

## Data in R: pC0 <- rep(0,3); PC0 <- diag(0.001,3,3)

gCoef[1:3] ~ dmnorm(gC0[], GC0[,])

## Data in R: gC0 <- rep(0,3); GC0 <- diag(0.001,3,3)

p.tau ~ dgamma(0.001, 0.001)

g.tau ~ dgamma(0.001, 0.001)

pt.tau ~ dgamma(0.001, 0.001)

gt.tau ~ dgamma(0.001, 0.001)

**Appendix S2**. Simulations procedure

We tested the assumptions and performance of our model under different scenarios by fitting it to simulated data with known occurrence and sampling patterns. We simulated data using the same sampling structure as for the real data, that is, daily replicates of visits during ten 90-day seasons at five sites, and using the observed increasing proportion of long lists through time (*PLL_t_*). We fitted the model to nine simulated datasets (scenarios hereafter; Table 1), each featuring a known combination of patterns in occupancy levels and sampling effort that may influence model performance but that are not explicitly accounted for into the model:

1. high, medium or low overall occupancy levels with variability among lakes in all other parameters but stable occupancy through time;
2. positive or negative trends on the persistence and colonization rates, resulting in 25% increase and decrease respectively.
3. increasing or decreasing number of visits over time (maintaining the variability in sampling effort among sites)
4. positive or negative trends (15%) in detection (and reporting) probabilities, besides those given by the observed *PLL_t_*.

**Table S3**: Colonization (g)-Persistence (p) Parameters (Eqn 4 and 5)

| **Scenario** | **1** | **2** | **3** | **4** | **5** | **6** | **7** | **8** | **9** |
| --- | --- | --- | --- | --- | --- | --- | --- | --- | --- |
| pCoef1 | 2.13 | 2.3 | -0.4 | 2.3 | | | | | |
| pCoef2 | 0 | | | -1 | 1 | 0 | | | |
| pCoef3 | -0.34 | 1.3 | 0.67 | 1.3 | | | | | |
| pCoef4 | -0.3 | -1.26 | -0.64 | -1.26 | | | | | |
| etaP[i] | Normal(0, 0.7) | | | | | | | | |
| gCoef1 | -0.21 | 1.4 | -2.1 | -0.21 | | | | | |
| gCoef2 | 0 | | | -1 | 1 | 0 | | | |
| gCoef3 | -0.03 | 2.1 | 0.4 | 2.1 | | | | | |
| gCoef4 | 0.01 | -2.55 | 0.04 | 2.55 | | | | | |
| etag[i] | Normal(0, 0.7) | | | | | | | | |
| Obs | High SU | Med SU | Low SU | Incr. SU | Decr. SU | Incr. NV | Decr. NV | Incr. Detect | Decr. Detect |

Note: no interannual variation was simulated. SU: Site Use; NV: Number of visits

Simulated dataset was randomly produced for each of the nine scenarios using known probability distributions for the occupancy, detection and colonization/extinction parameters. The colonization extinction parameters (Table S3, Fig. S2) were the multi-annual average of the estimated parameters for *Anas streptera* (Scn 1), *Locustella naevia* (Scn 2, 4-9) and *Asio otus* (Scn 3). For randomizations we used the Random seed = 1234. Simulations were all initiated with Initial Occupancy Status (First day of each year) = 0.


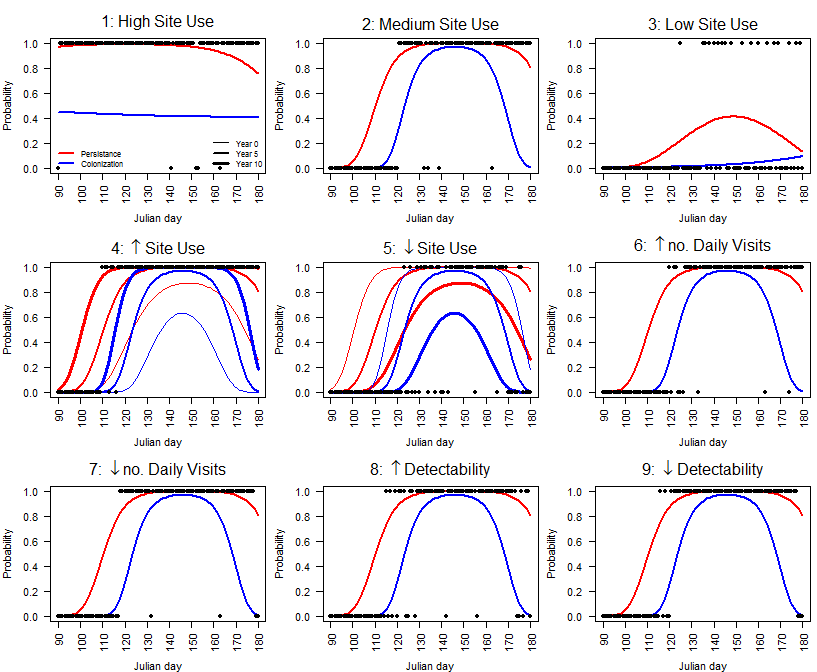


**Figure S4.** Simulated occupancy data (dots) given daily persistence (red lines) and colonization rates (blue lines). Examples shown here are simulated data from Year 1, Site 1.

Once daily occupancy data was simulated, the observation process was simulated for each visit. For each day, a random number of visits was drawn from a Poisson distribution constrained to the range [1,50] with mean λ = Int + Slp × t + ε_i_, where Int is the intercept and Slp is the slope of a linear function of time and ε_i_ is random variability for site i. For scenarios 1 to 5, 8 and 9 the mean number of daily visits (Int) was constant at 5. For scenarios 6 and 7 the parameters were Int = 0 and 30, and Slp = 3 and -3, respectively. Random variability ε was defined once for each site as Poisson (λ=2), and used consistently across the simulations. An *SLL* category was assigned to each visit defined from the observed proportion of Long species list (*SLL* ≥ 10), Short species list (5 ≥ *SLL* < 10), and Single observations (*SLL* < 5), in such way that if the number of visits per day was lower than three, only single observations were produced. Then the actual number of observed species was randomly draw from the *SLL* category range (e.g. Single observations = Discrete Uniform {1, 4}; Short Species Lists = Discrete Uniform {5, 9}; Long Species Lists = Discrete Uniform {10, 45}). Similar to field observations, the maximum species list allowed was 45.

Each visit’s probability of detection of the assumed focal species was calculated with the same saturation function assumed in the model used in the main article. The assumed mean half-saturation parameter, δ, were drawn from a continuous uniform distribution, Uniform{3, 4}, once for each site used consistently across the simulations. The only difference between Scenarios 1 to 7 and Scenarios 8 and 9 is that δ changed not only as a function of *PLL* (effect parameter = -3, for an increase in detection probability), but also as a function of time (effect parameter = -0.15 and 0.15). Note that time (years) and *PLL* were not standardized therefore effect parameters are not comparable. Negative effect parameters decrease δ thus increase the species detectability. Finally, detections for each visit were calculated as the simulated occupancy status times a draw from a Bernoulli trial with *p* equal to each visit’s detection probability.

We fitted the same model described in the main article to the data under the nine scenarios and evaluated the goodness-of-fit of the models and the ability of the models to estimate the known occurrence data in the same way as described in the main article.

**Appendix S3**. Separated PDF file with local annual estimates for 71 wetland bird species. **Common Legend**. Estimated annual occupancy (green) using only long species lists (SLL ≥ 10; cyan) and mean site use using the full dataset (black), over the study region (nine sites). Solid lines and shaded areas show the median and 95% CI around the modelled occupancy and mean site use, respectively. Black dots indicate observed mean site use, and red whiskers are replicated observation data. First panel (upper left) shows the summary over the region, next nine panels show local estimates. Second to last panel shows discrepancy between observed and predicted site use. Last panel (bottom right) shows the “posterior predictive check” for all sites.
