# Supplementary material for: Exploiting opportunistic observations to estimate changes in seasonal site use: An example with wetland birds
Source: Ecol Evol. 2017 Jun 15;7(15):5632–44. doi: 10.1002/ece3.3100 (PMC5551100; doi:10.1002/ece3.3100)
Supplement: Supplementary file 2 [file ECE3-7-5632-s002.pdf]

Acrocephalus arundinaceus

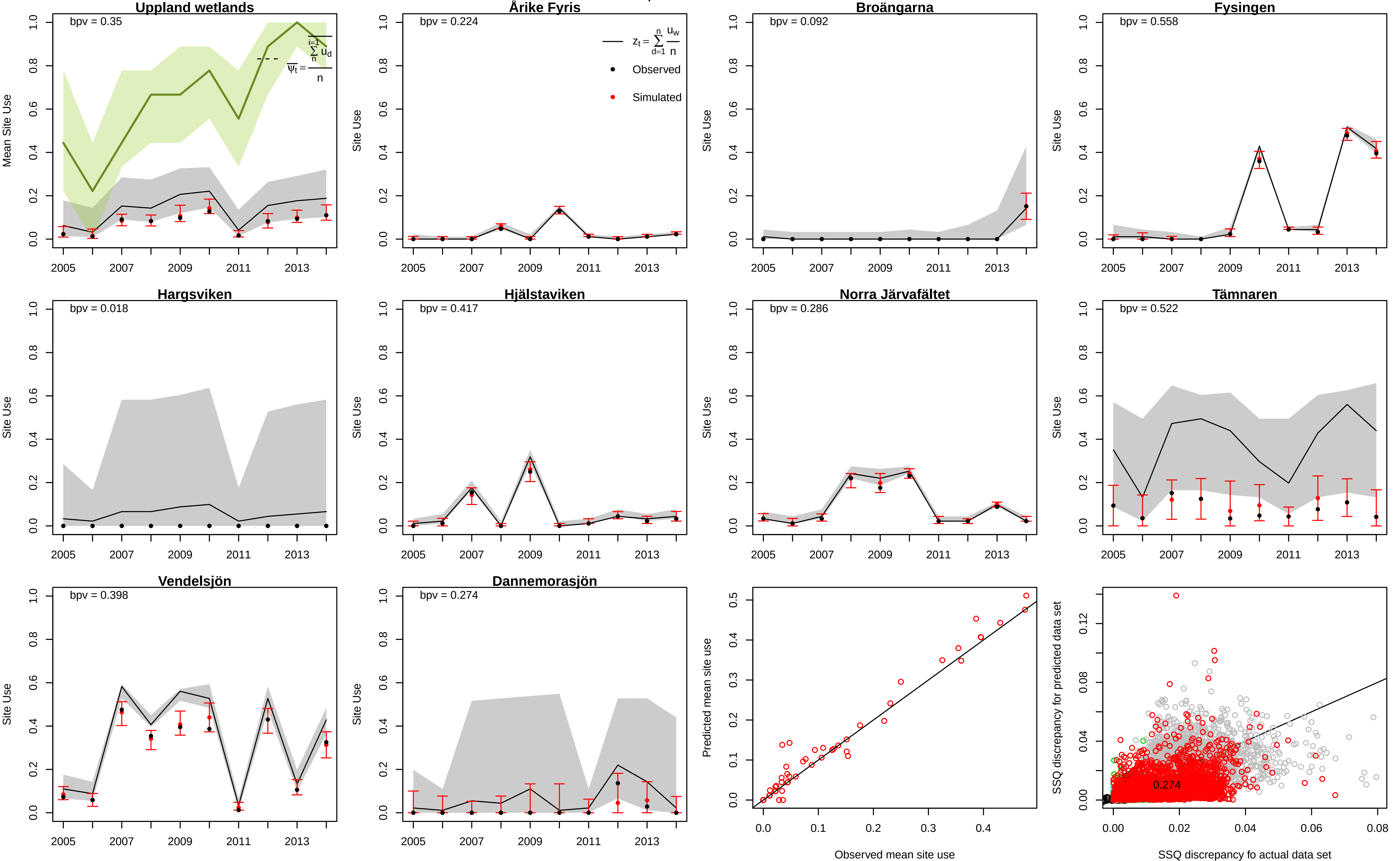

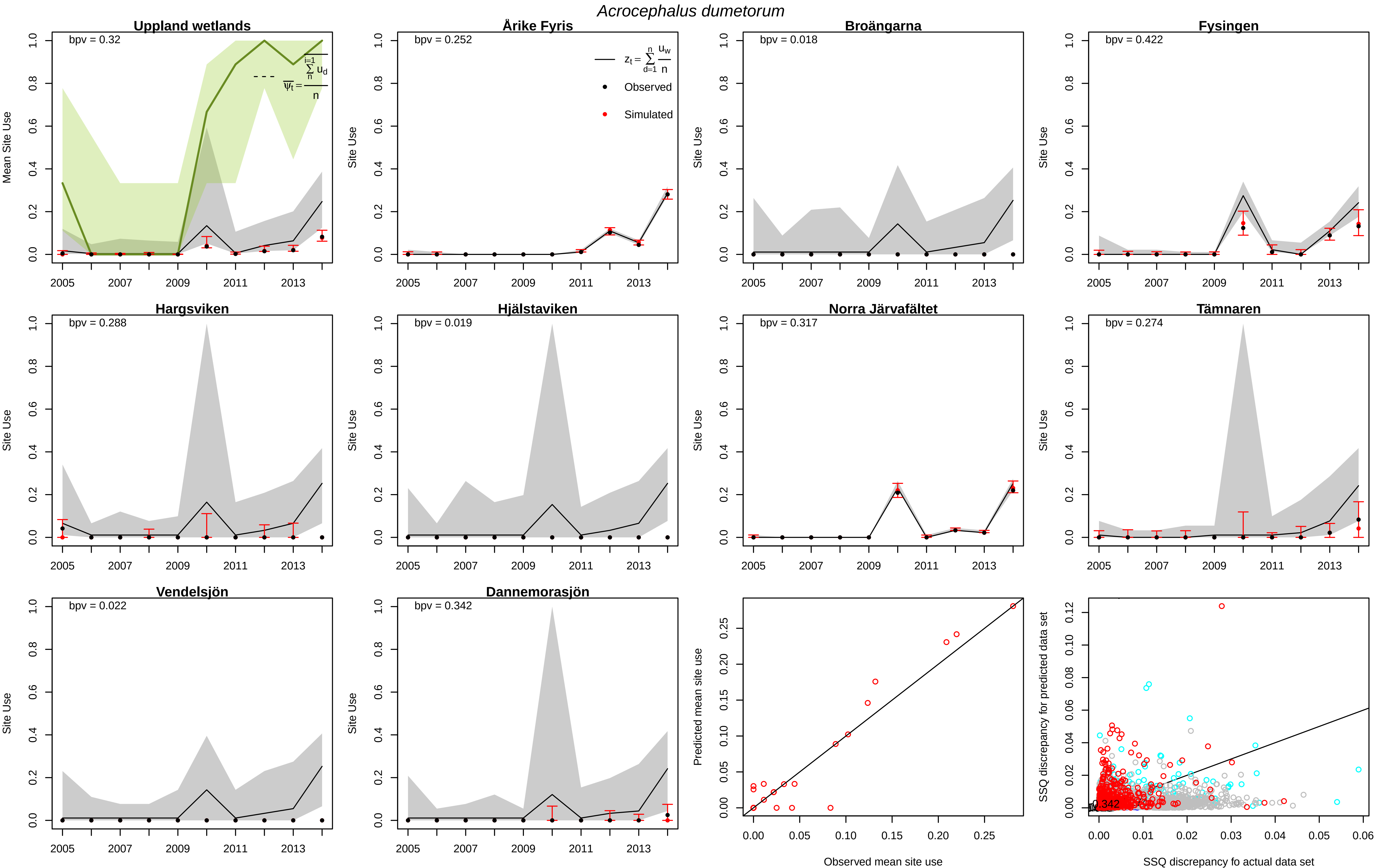
Acrocephalus dumetorum
Uppland wetlands
bpv = 0.32
Mean Site Use
Årike Fyris
bpv = 0.252
Observed
Simulated
Broängarna
bpv = 0.018
Fysingen
bpv = 0.422
Hargsviken
bpv = 0.288
Hjälstaviken
bpv = 0.019
Norra Järvafältet
bpv = 0.317
Tämnaren
bpv = 0.274
Vendelsjön
bpv = 0.022
Dannemorasjön
bpv = 0.342
Site Use
Predicted mean site use
Observed mean site use
SSQ discrepancy for predicted data set
SSQ discrepancy fo actual data set

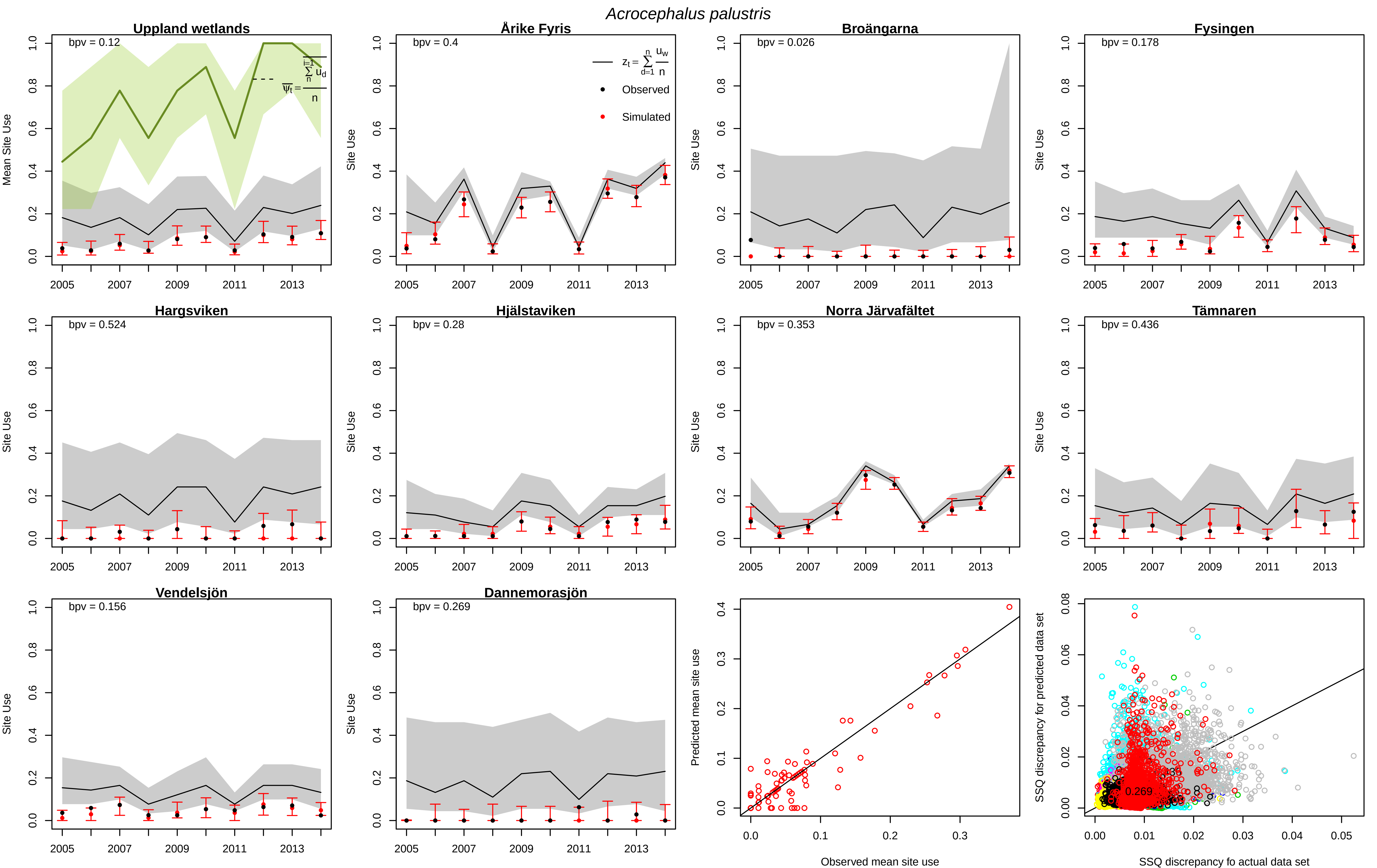

Acrocephalus palustris
Uppland wetlands
bpv = 0.12
Mean Site Use
Årike Fyris
bpv = 0.4
Observed
Simulated
Broängarna
bpv = 0.026
Fysingen
bpv = 0.178
Hargsviken
bpv = 0.524
Hjälstaviken
bpv = 0.28
Norra Järvafältet
bpv = 0.353
Tämnaren
bpv = 0.436
Vendelsjön
bpv = 0.156
Dannemorasjön
bpv = 0.269
Site Use
Predicted mean site use
Observed mean site use
SSQ discrepancy for predicted data set
SSQ discrepancy fo actual data set
0.269

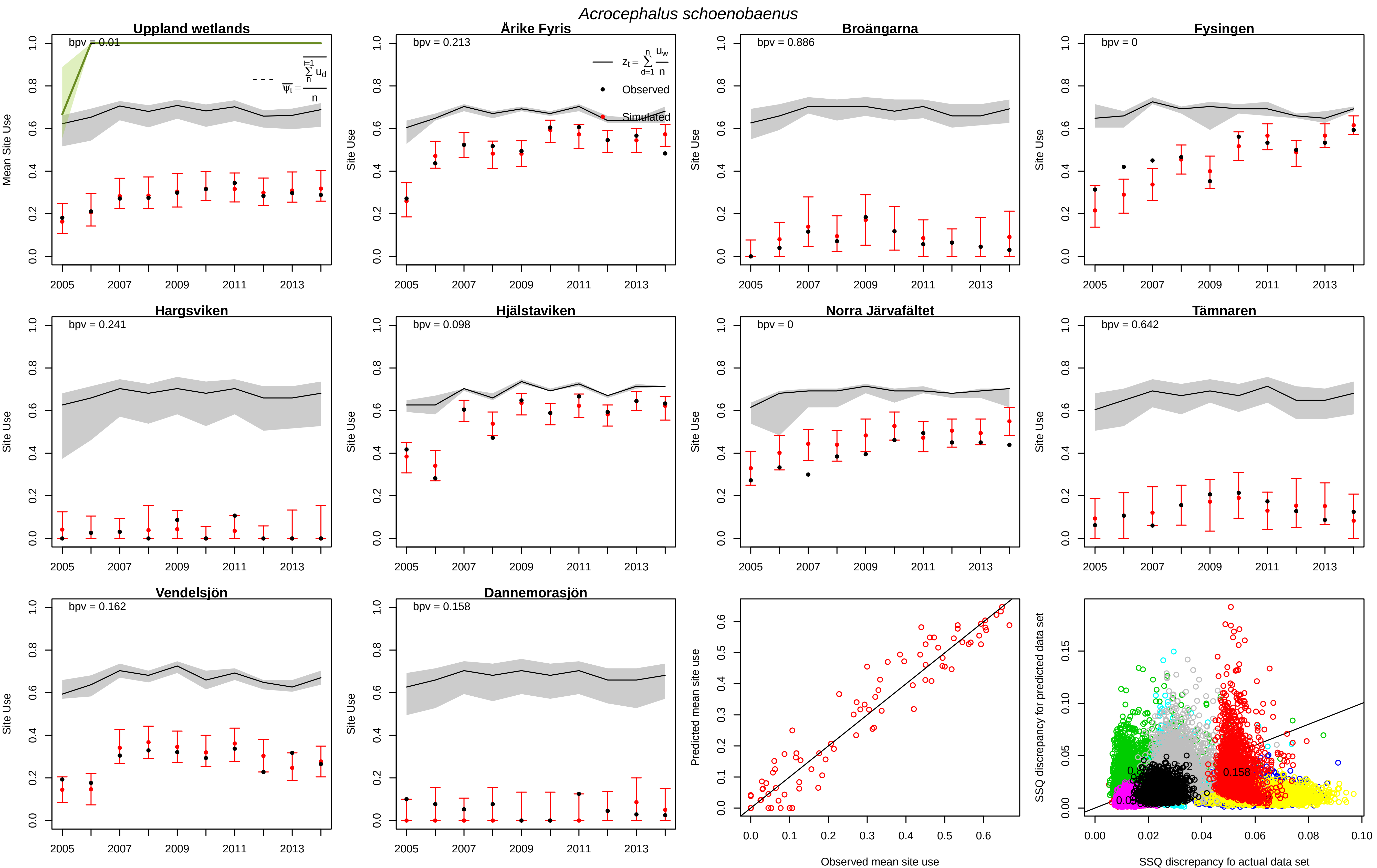

Acrocephalus schoenobaenus
Uppland wetlands
Årike Fyris
Broängarna
Fysingen
Hargsviken
Hjälstaviken
Norra Järvafältet
Tämnaren
Vendelsjön
Dannemorasjön
bpv = 0.01
bpv = 0.213
bpv = 0.886
bpv = 0
bpv = 0.241
bpv = 0.098
bpv = 0
bpv = 0.642
bpv = 0.162
bpv = 0.158
Observed
Simulated
Mean Site Use
Site Use
Predicted mean site use
Observed mean site use
SSQ discrepancy for predicted data set
SSQ discrepancy fo actual data set

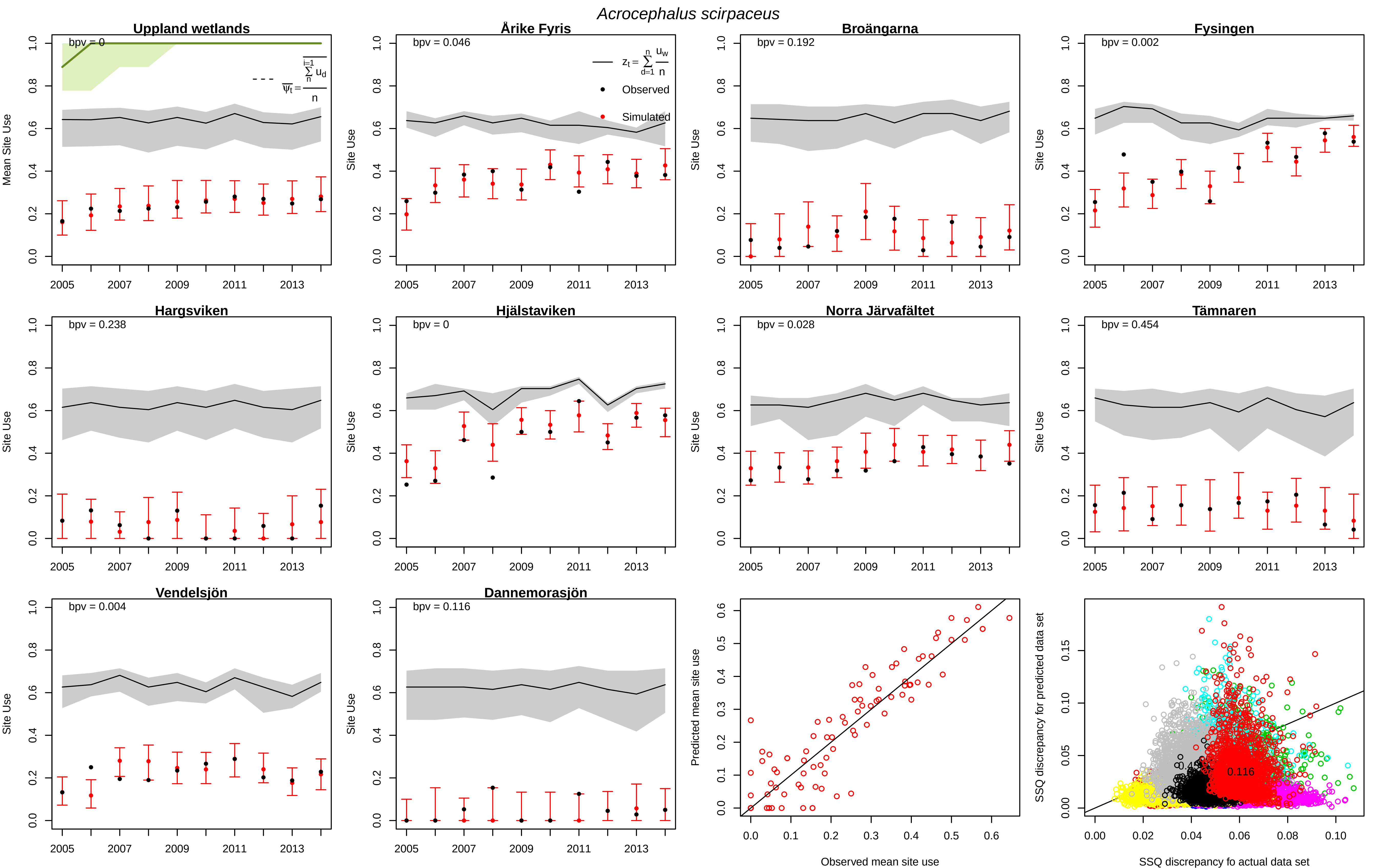
Acrocephalus scirpaceus
Uppland wetlands
bpv = 0
Mean Site Use
Årike Fyris
bpv = 0.046
Observed
Simulated
Site Use
Broängarna
bpv = 0.192
Fysingen
bpv = 0.002
Hargsviken
bpv = 0.238
Hjälstaviken
bpv = 0
Norra Järvafältet
bpv = 0.028
Tämnaren
bpv = 0.454
Vendelsjön
bpv = 0.004
Dannemorasjön
bpv = 0.116
2005
2007
2009
2011
2013
Predicted mean site use
Observed mean site use
SSQ discrepancy for predicted data set
SSQ discrepancy fo actual data set
0.116

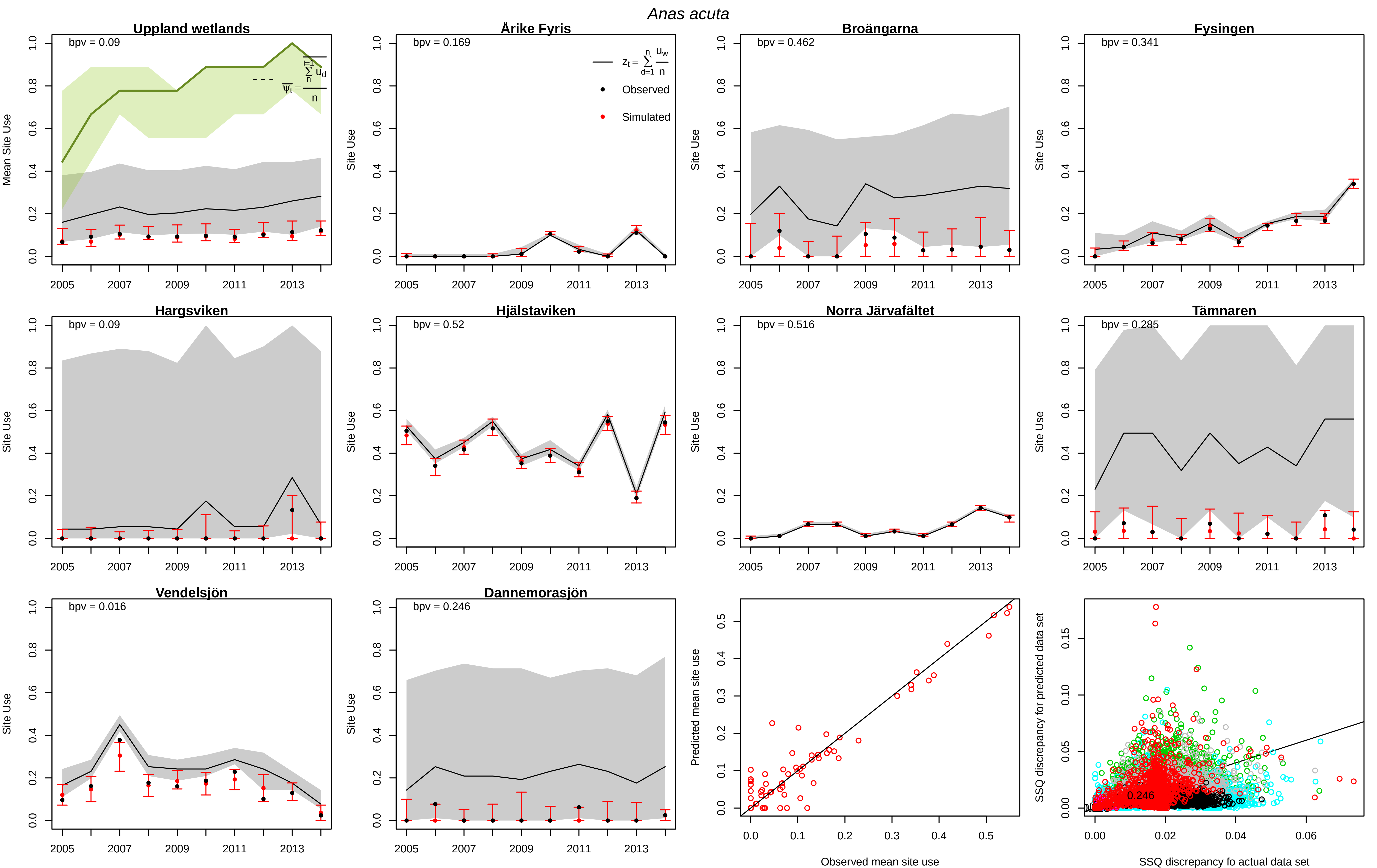

Anas acuta
Uppland wetlands
bpv = 0.09
Mean Site Use
Årike Fyris
bpv = 0.169
Observed
Simulated
Broängarna
bpv = 0.462
Fysingen
bpv = 0.341
Hargsviken
bpv = 0.09
Hjälstaviken
bpv = 0.52
Norra Järvafältet
bpv = 0.516
Tämnaren
bpv = 0.285
Vendelsjön
bpv = 0.016
Dannemorasjön
bpv = 0.246
Site Use
Predicted mean site use
Observed mean site use
SSQ discrepancy for predicted data set
SSQ discrepancy fo actual data set
0.246

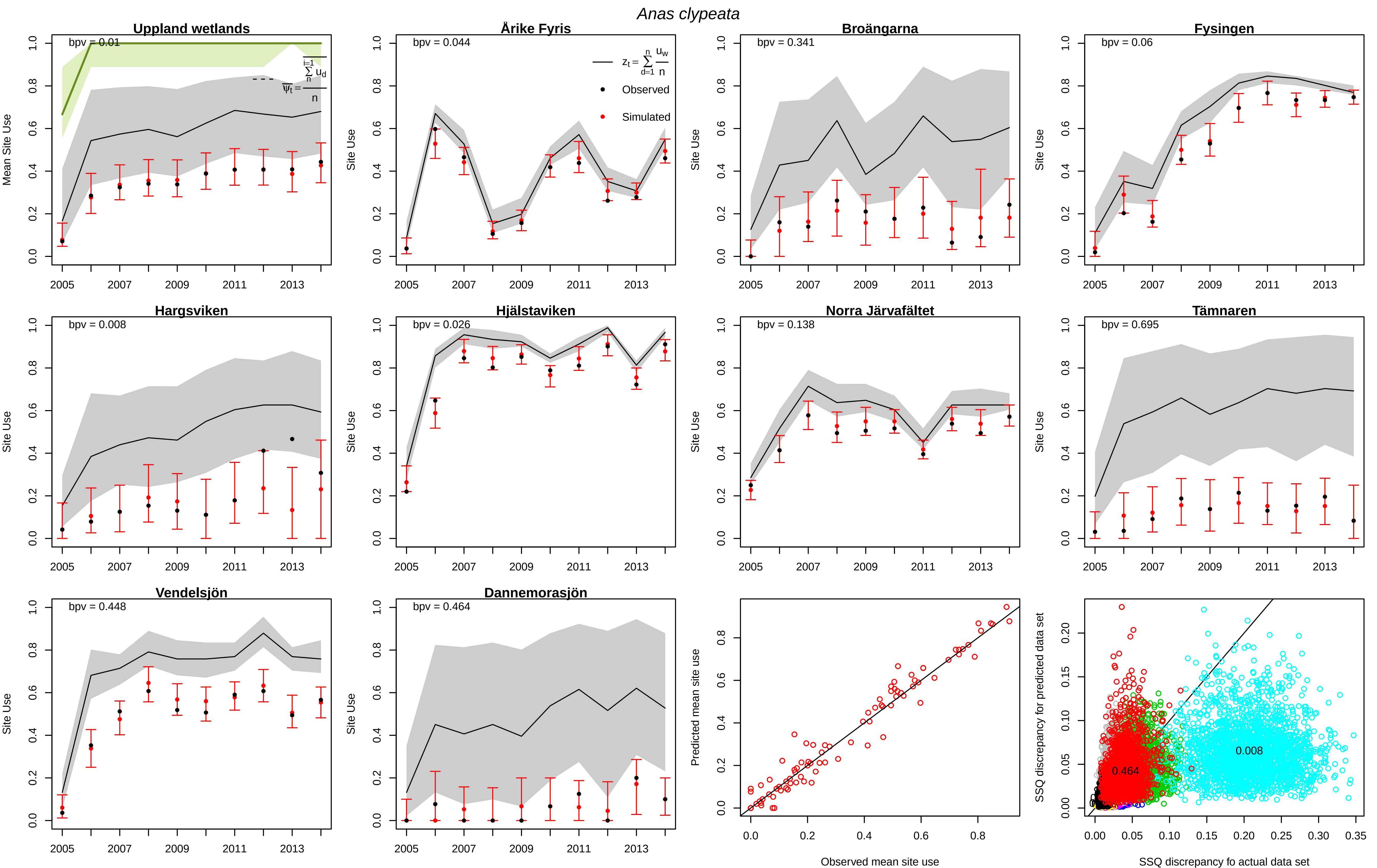

Anas clypeata
Uppland wetlands
bpv = 0.01
Årike Fyris
bpv = 0.044
Observed
Simulated
Broängarna
bpv = 0.341
Fysingen
bpv = 0.06
Hargsviken
bpv = 0.008
Hjälstaviken
bpv = 0.026
Norra Järvafältet
bpv = 0.138
Tämnaren
bpv = 0.695
Vendelsjön
bpv = 0.448
Dannemorasjön
bpv = 0.464
Mean Site Use
Site Use
Predicted mean site use
Observed mean site use
SSQ discrepancy for predicted data set
SSQ discrepancy fo actual data set
0.008
0.464

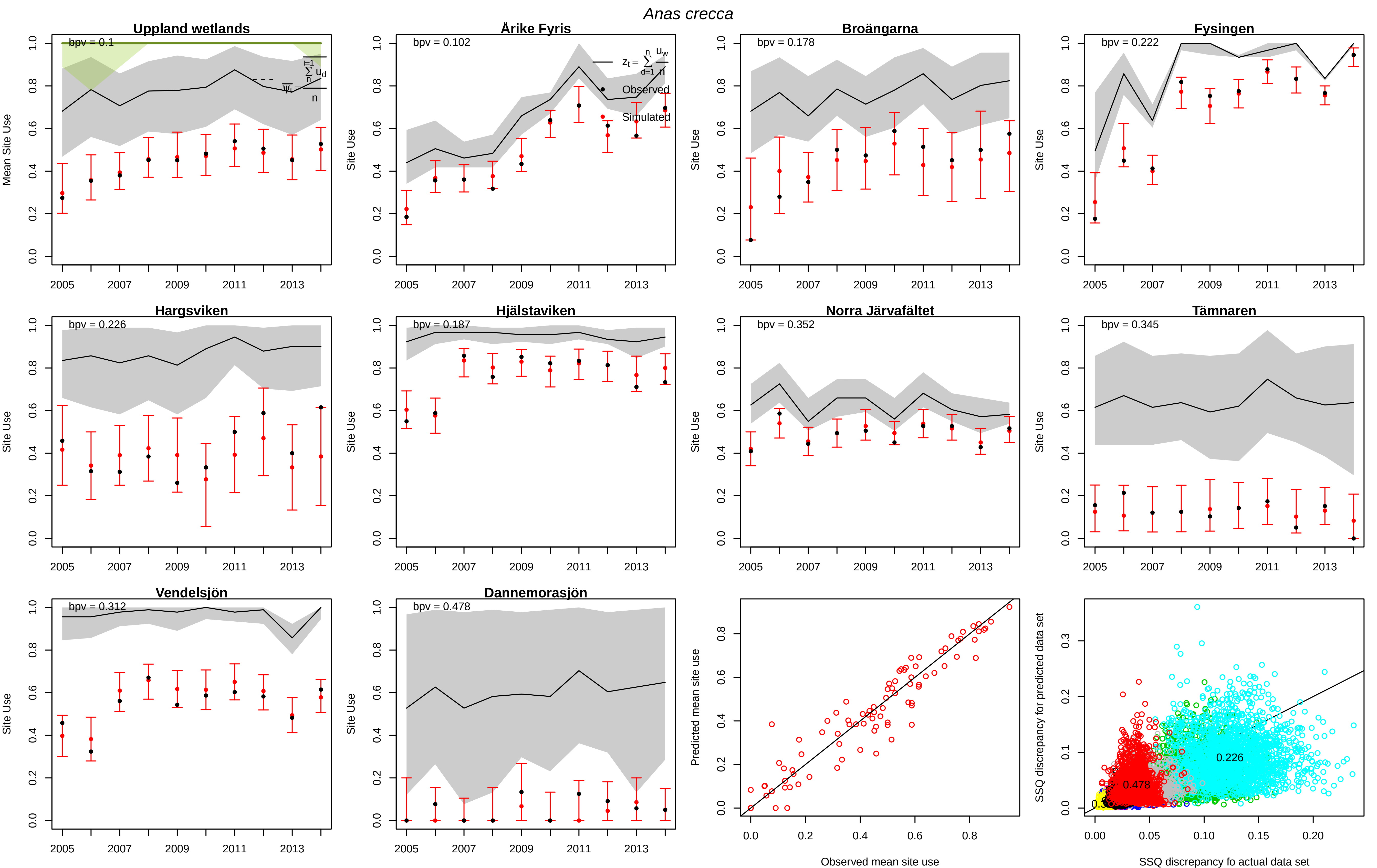

Anas crecca
Uppland wetlands
bpv = 0.1
Mean Site Use
Årike Fyris
bpv = 0.102
Observed
Simulated
Broängarna
bpv = 0.178
Fysingen
bpv = 0.222
Hargsviken
bpv = 0.226
Hjälstaviken
bpv = 0.187
Norra Järvafältet
bpv = 0.352
Tämnaren
bpv = 0.345
Vendelsjön
bpv = 0.312
Dannemorasjön
bpv = 0.478
Site Use
Predicted mean site use
Observed mean site use
SSQ discrepancy for predicted data set
SSQ discrepancy fo actual data set
0.478
0.226

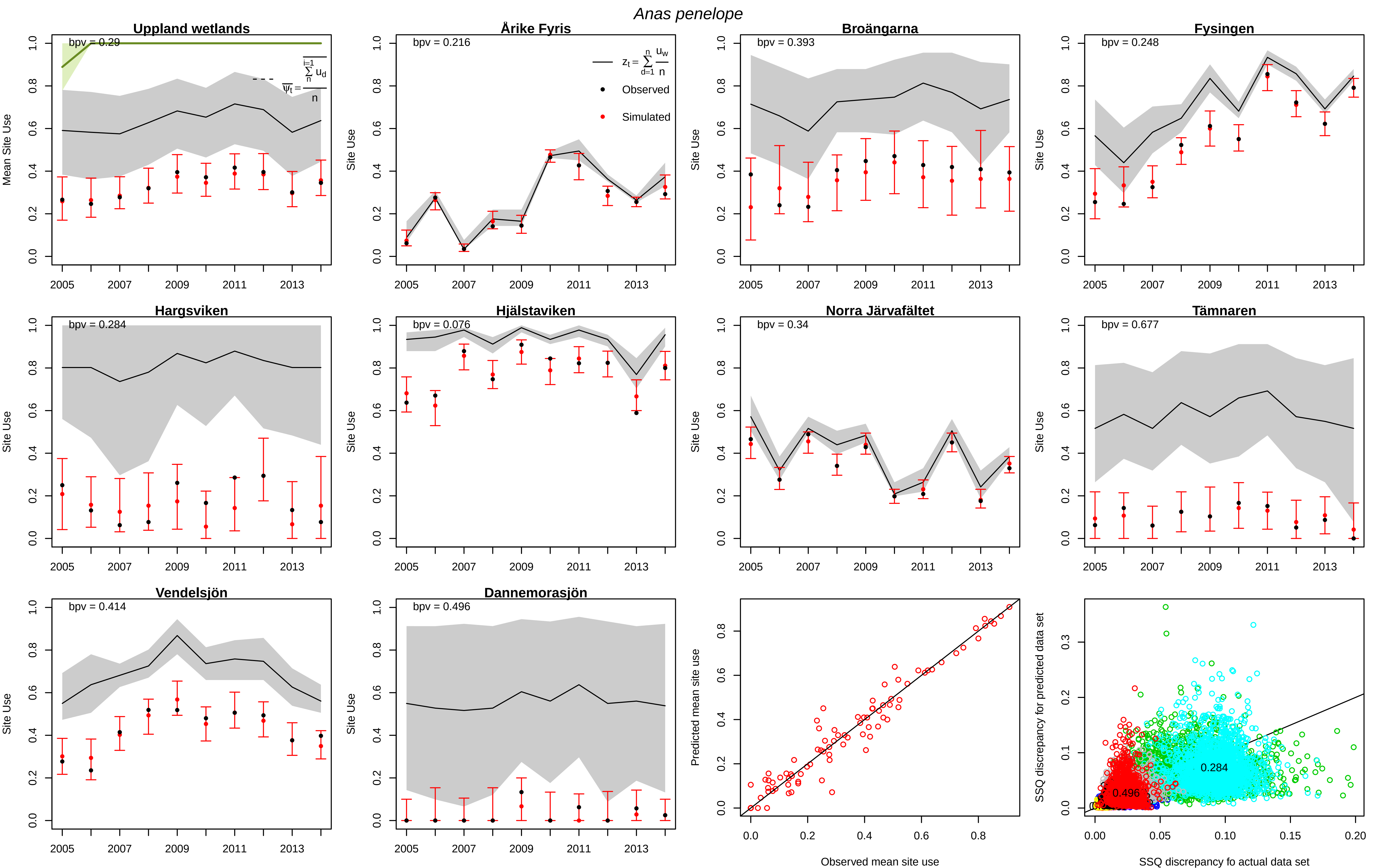
Anas penelope
Uppland wetlands
bpv = 0.29
Mean Site Use
Årike Fyris
bpv = 0.216
Observed
Simulated
Broängarna
bpv = 0.393
Fysingen
bpv = 0.248
Hargsviken
bpv = 0.284
Hjälstaviken
bpv = 0.076
Norra Järvafältet
bpv = 0.34
Tämnaren
bpv = 0.677
Vendelsjön
bpv = 0.414
Dannemorasjön
bpv = 0.496
Site Use
2005
2007
2009
2011
2013
Predicted mean site use
Observed mean site use
SSQ discrepancy for predicted data set
SSQ discrepancy fo actual data set
0.284
0.496

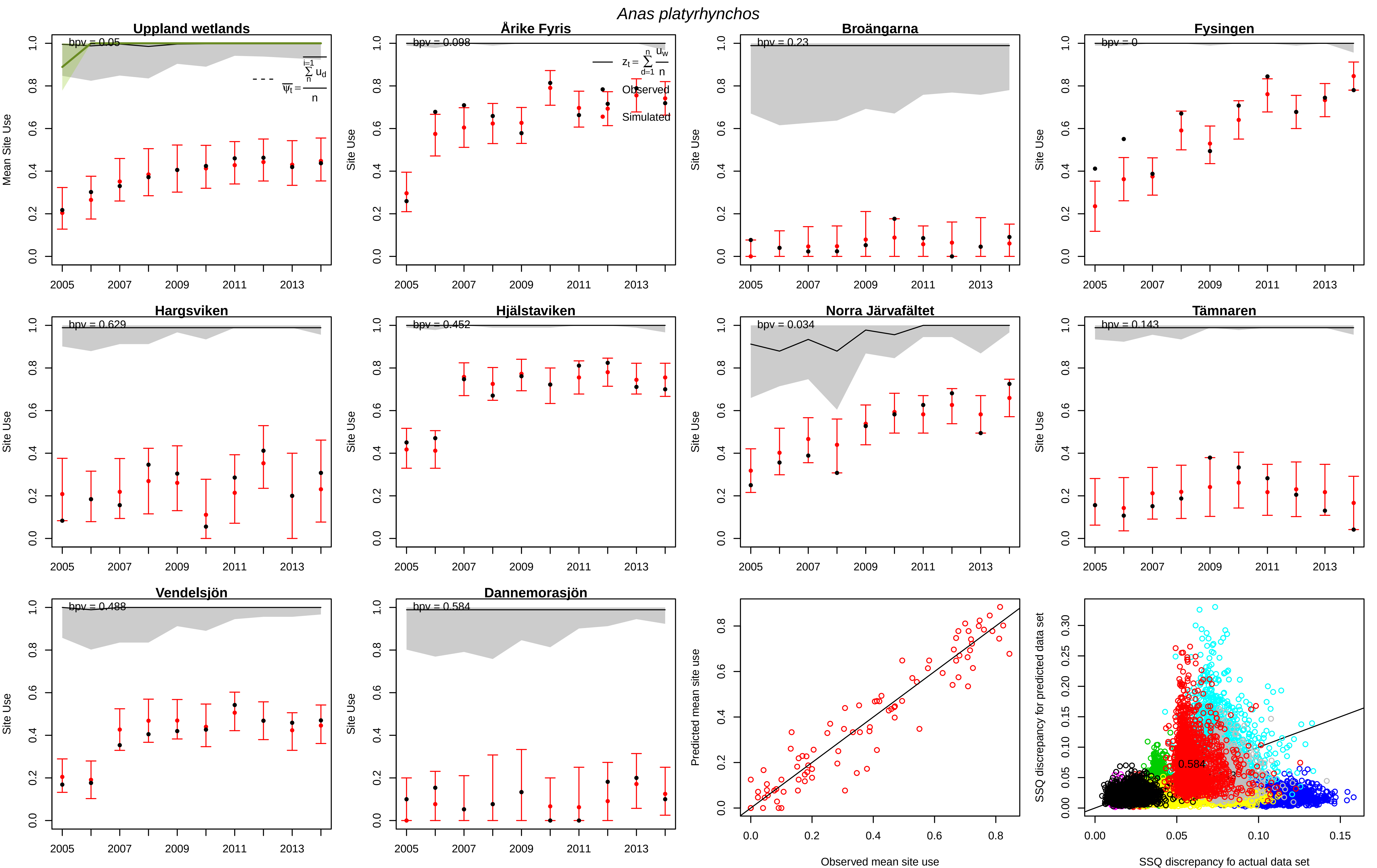
Anas platyrhynchos
Uppland wetlands
bpv = 0.05
Mean Site Use
Årike Fyris
bpv = 0.098
Observed
Simulated
Broängarna
bpv = 0.23
Fysingen
bpv = 0
Hargsviken
bpv = 0.629
Hjälstaviken
bpv = 0.452
Norra Järvafältet
bpv = 0.034
Tämnaren
bpv = 0.143
Vendelsjön
bpv = 0.488
Dannemorasjön
bpv = 0.584
Site Use
Predicted mean site use
Observed mean site use
SSQ discrepancy for predicted data set
SSQ discrepancy fo actual data set
0.584

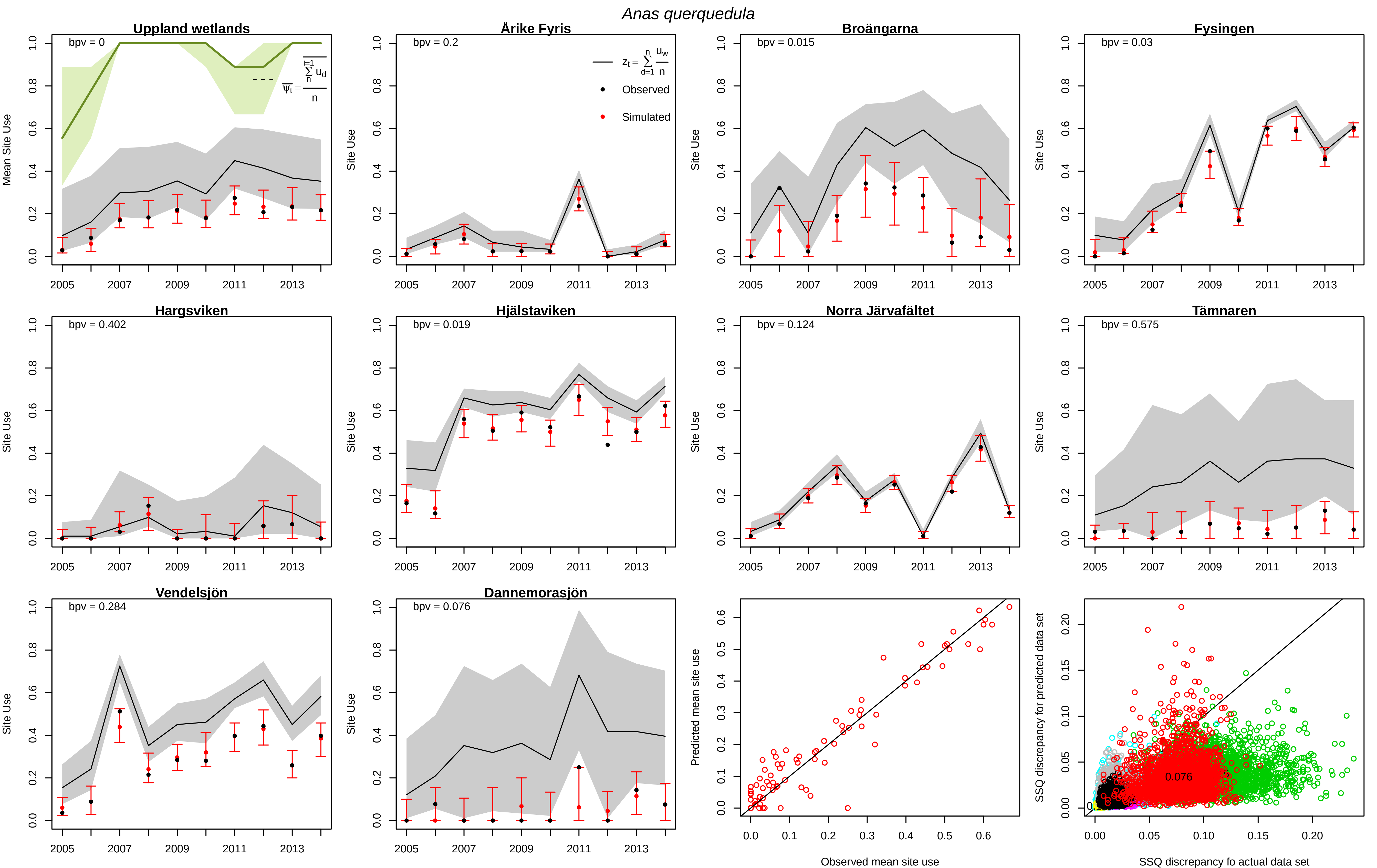

Anas querquedula
Uppland wetlands
bpv = 0
Mean Site Use
Årike Fyris
bpv = 0.2
Observed
Simulated
Broängarna
bpv = 0.015
Fysingen
bpv = 0.03
Hargsviken
bpv = 0.402
Hjälstaviken
bpv = 0.019
Norra Järvafältet
bpv = 0.124
Tämnaren
bpv = 0.575
Vendelsjön
bpv = 0.284
Dannemorasjön
bpv = 0.076
Site Use
Predicted mean site use
Observed mean site use
SSQ discrepancy for predicted data set
SSQ discrepancy fo actual data set
0.076

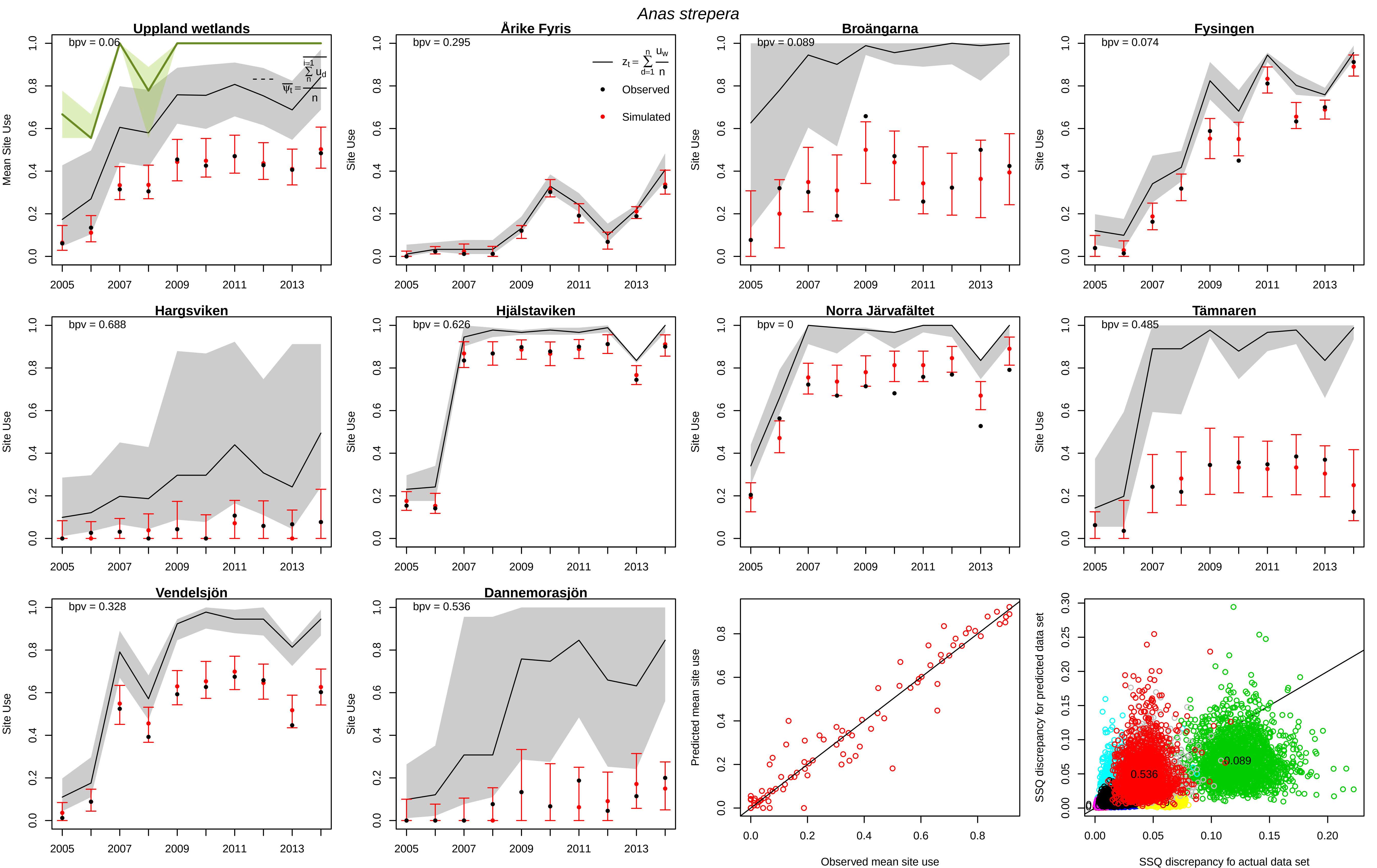

Anas strepera
Uppland wetlands
bpv = 0.06
Mean Site Use
Årike Fyris
bpv = 0.295
Observed
Simulated
Broängarna
bpv = 0.089
Fysingen
bpv = 0.074
Hargsviken
bpv = 0.688
Hjälstaviken
bpv = 0.626
Norra Järvafältet
bpv = 0
Tämnaren
bpv = 0.485
Vendelsjön
bpv = 0.328
Dannemorasjön
bpv = 0.536
Site Use
Predicted mean site use
Observed mean site use
0.536
0.089
SSQ discrepancy for predicted data set
SSQ discrepancy fo actual data set

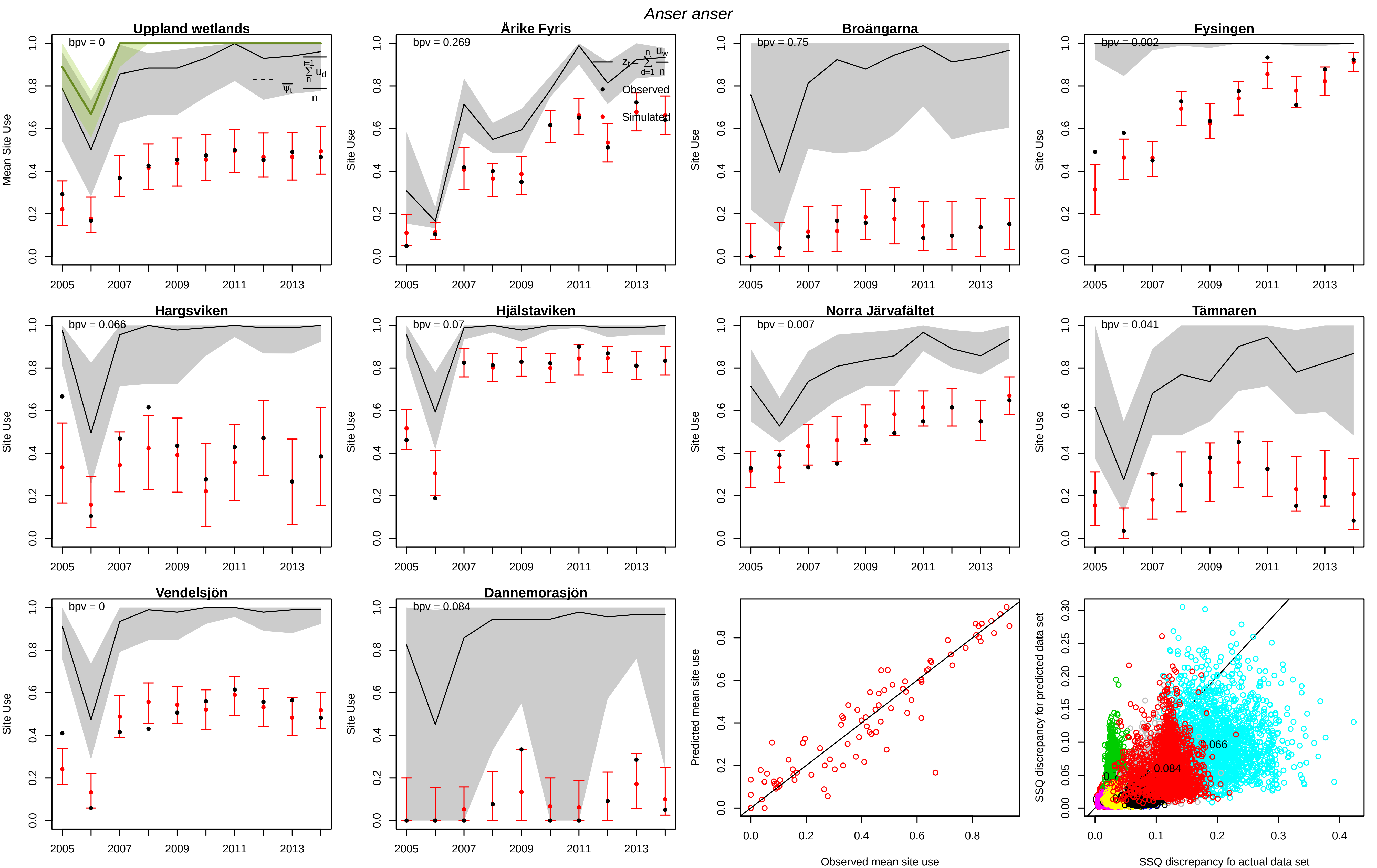
Anser anser
Uppland wetlands
bpv = 0
Mean Site Use
Årike Fyris
bpv = 0.269
Observed
Simulated
Broängarna
bpv = 0.75
Fysingen
bpv = 0.002
Hargsviken
bpv = 0.066
Hjälstaviken
bpv = 0.07
Norra Järvafältet
bpv = 0.007
Tämnaren
bpv = 0.041
Vendelsjön
bpv = 0
Dannemorasjön
bpv = 0.084
Site Use
Predicted mean site use
Observed mean site use
SSQ discrepancy for predicted data set
SSQ discrepancy fo actual data set

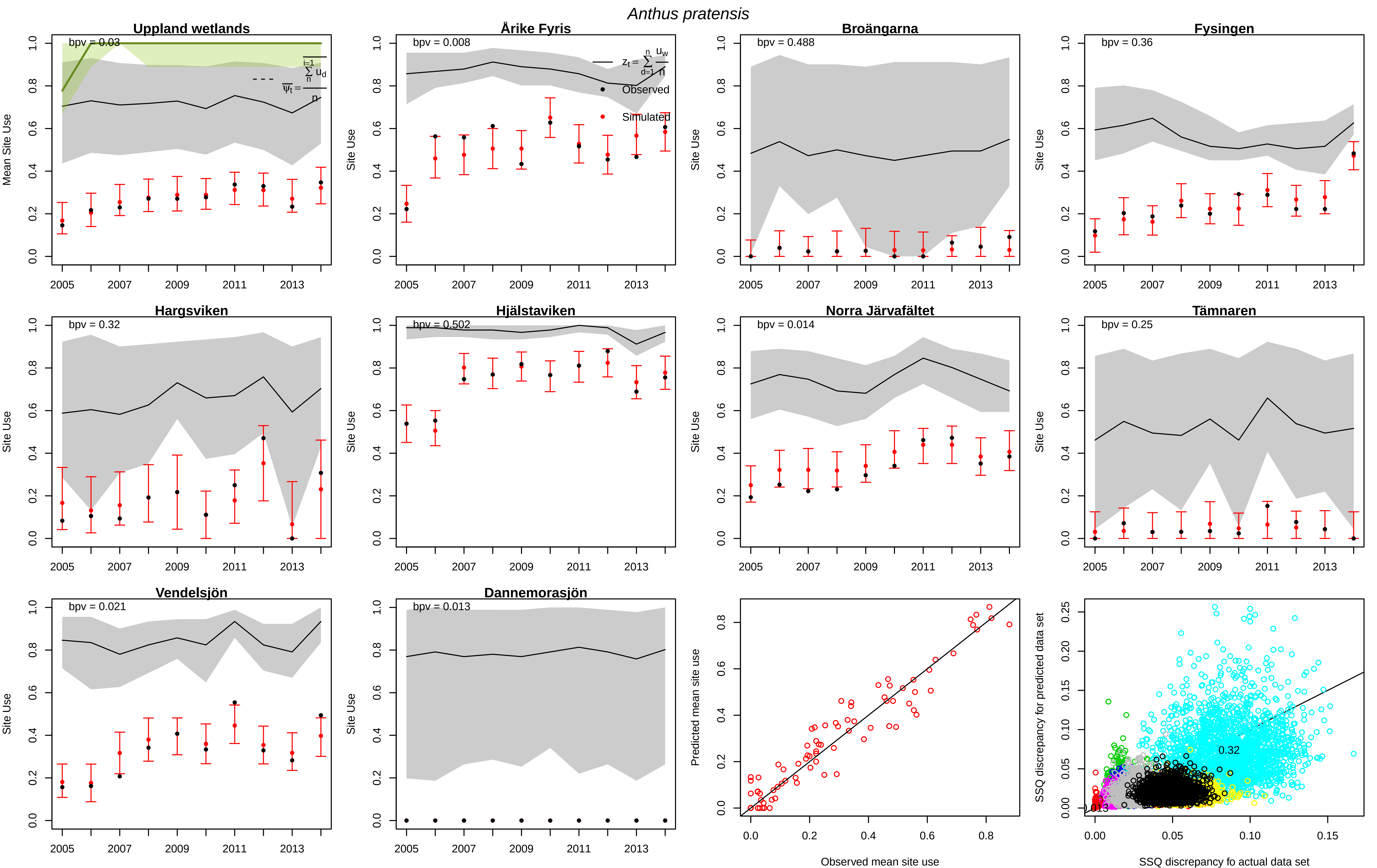

Anthus pratensis
Uppland wetlands
bpv = 0.03
Mean Site Use
Årike Fyris
bpv = 0.008
Observed
Simulated
Broängarna
bpv = 0.488
Fysingen
bpv = 0.36
Hargsviken
bpv = 0.32
Hjälstaviken
bpv = 0.502
Norra Järvafältet
bpv = 0.014
Tämnaren
bpv = 0.25
Vendelsjön
bpv = 0.021
Dannemorasjön
bpv = 0.013
Site Use
2005
2007
2009
2011
2013
Predicted mean site use
Observed mean site use
SSQ discrepancy for predicted data set
SSQ discrepancy fo actual data set
0.32

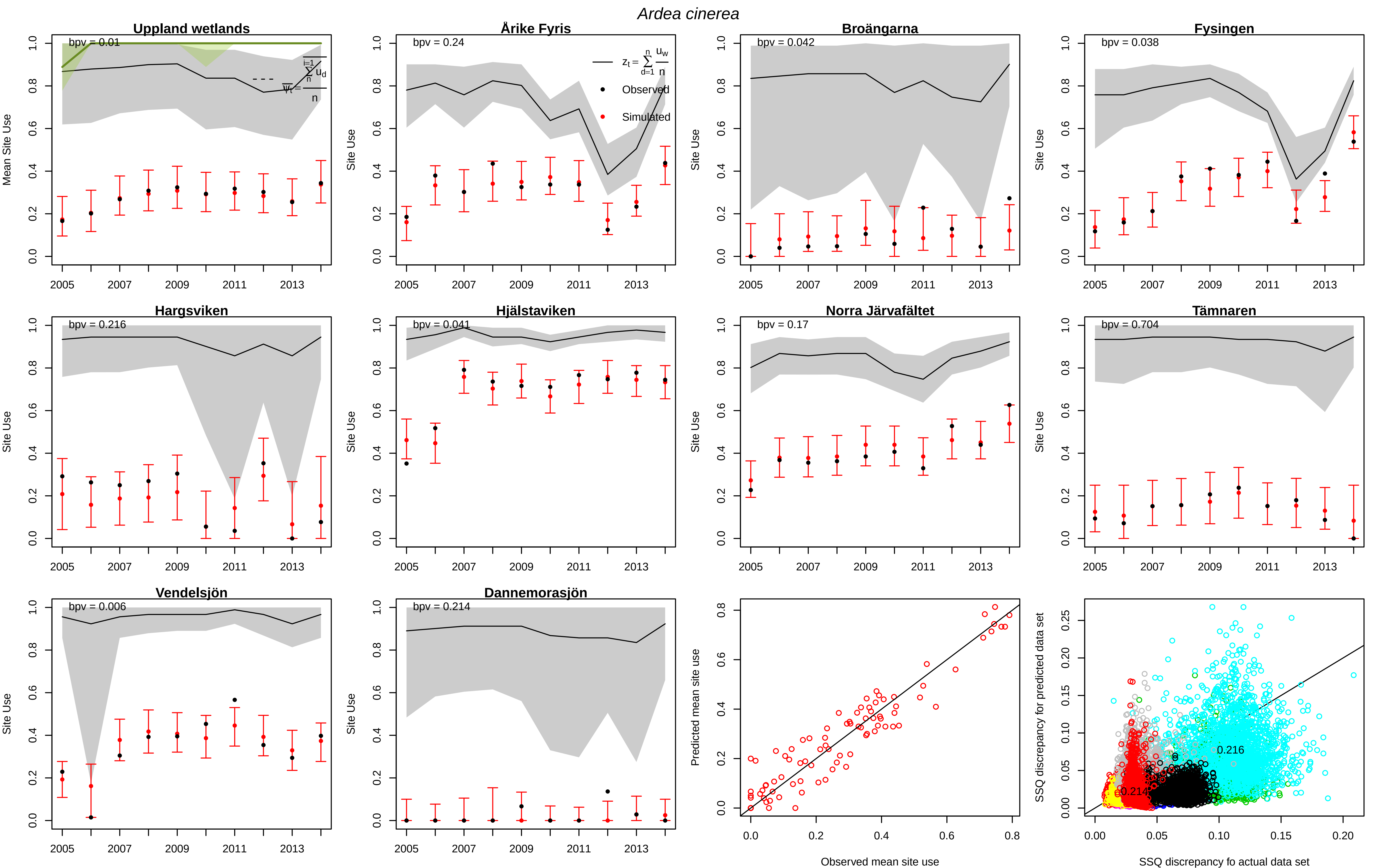
Ardea cinerea
Uppland wetlands
bpv = 0.01
Mean Site Use
Årike Fyris
bpv = 0.24
Observed
Simulated
Broängarna
bpv = 0.042
Fysingen
bpv = 0.038
Hargsviken
bpv = 0.216
Hjälstaviken
bpv = 0.041
Norra Järvafältet
bpv = 0.17
Tämnaren
bpv = 0.704
Vendelsjön
bpv = 0.006
Dannemorasjön
bpv = 0.214
Site Use
Predicted mean site use
Observed mean site use
SSQ discrepancy for predicted data set
SSQ discrepancy fo actual data set
0.216
0.214

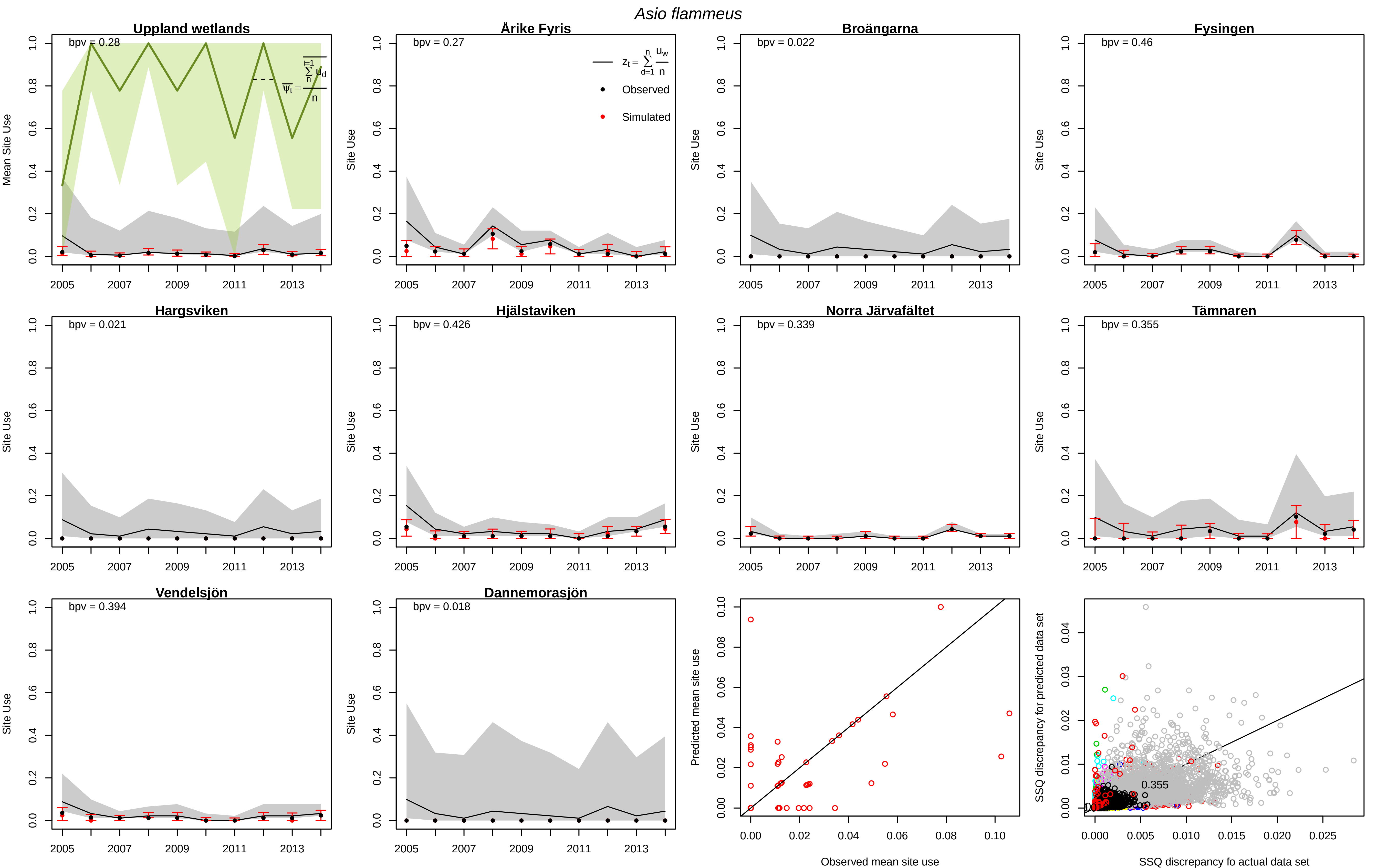

Asio flammeus
Uppland wetlands
bpv = 0.28
Mean Site Use
Årike Fyris
bpv = 0.27
Observed
Simulated
Broängarna
bpv = 0.022
Fysingen
bpv = 0.46
Hargsviken
bpv = 0.021
Hjälstaviken
bpv = 0.426
Norra Järvafältet
bpv = 0.339
Tämnaren
bpv = 0.355
Vendelsjön
bpv = 0.394
Dannemorasjön
bpv = 0.018
Site Use
Predicted mean site use
Observed mean site use
SSQ discrepancy for predicted data set
SSQ discrepancy fo actual data set
0.355

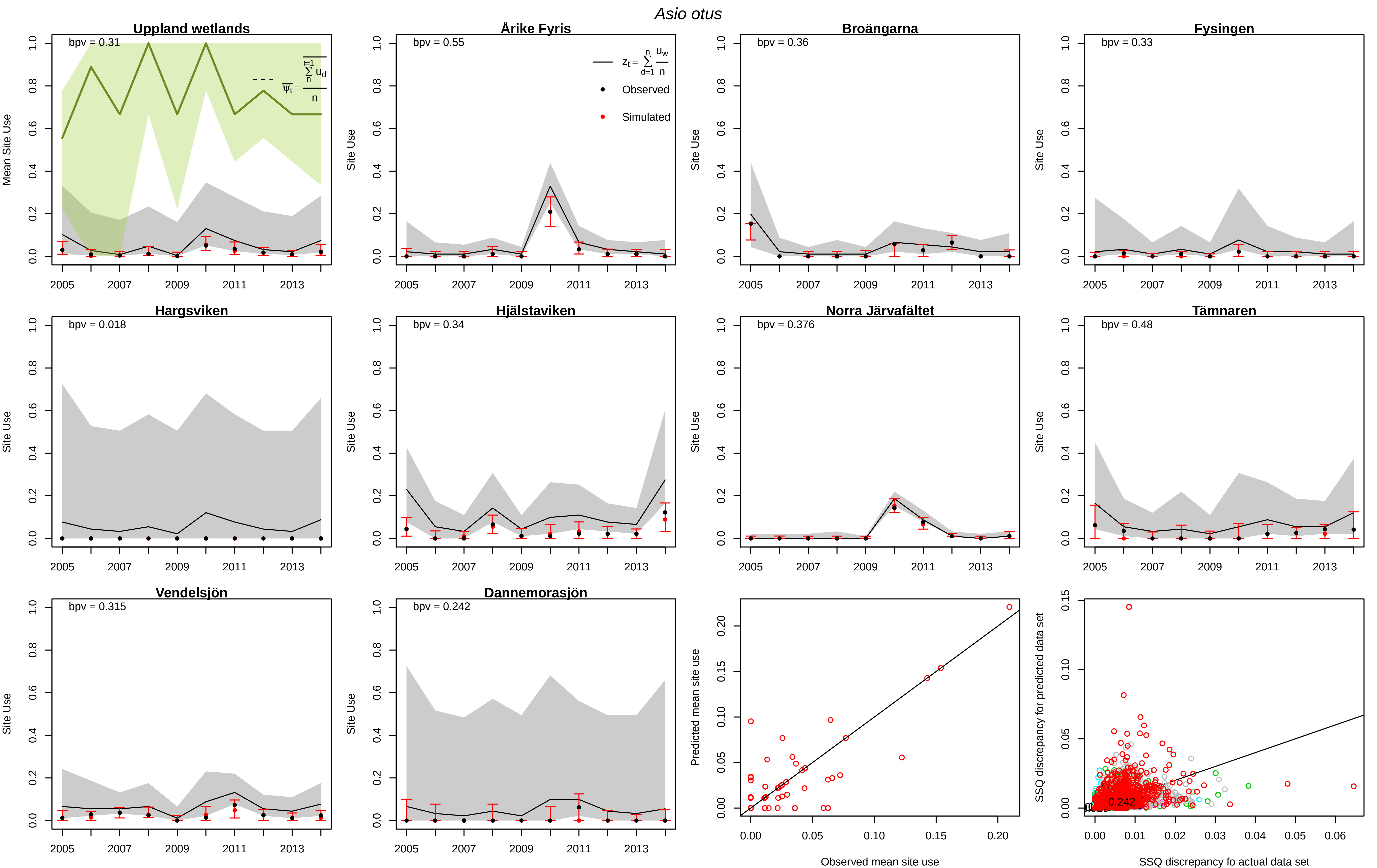

Asio otus
Uppland wetlands
bpv = 0.31
Mean Site Use
Årike Fyris
bpv = 0.55
Observed
Simulated
Broängarna
bpv = 0.36
Fysingen
bpv = 0.33
Hargsviken
bpv = 0.018
Hjälstaviken
bpv = 0.34
Norra Järvafältet
bpv = 0.376
Tämnaren
bpv = 0.48
Vendelsjön
bpv = 0.315
Dannemorasjön
bpv = 0.242
Site Use
Predicted mean site use
Observed mean site use
SSQ discrepancy for predicted data set
SSQ discrepancy fo actual data set
0.242

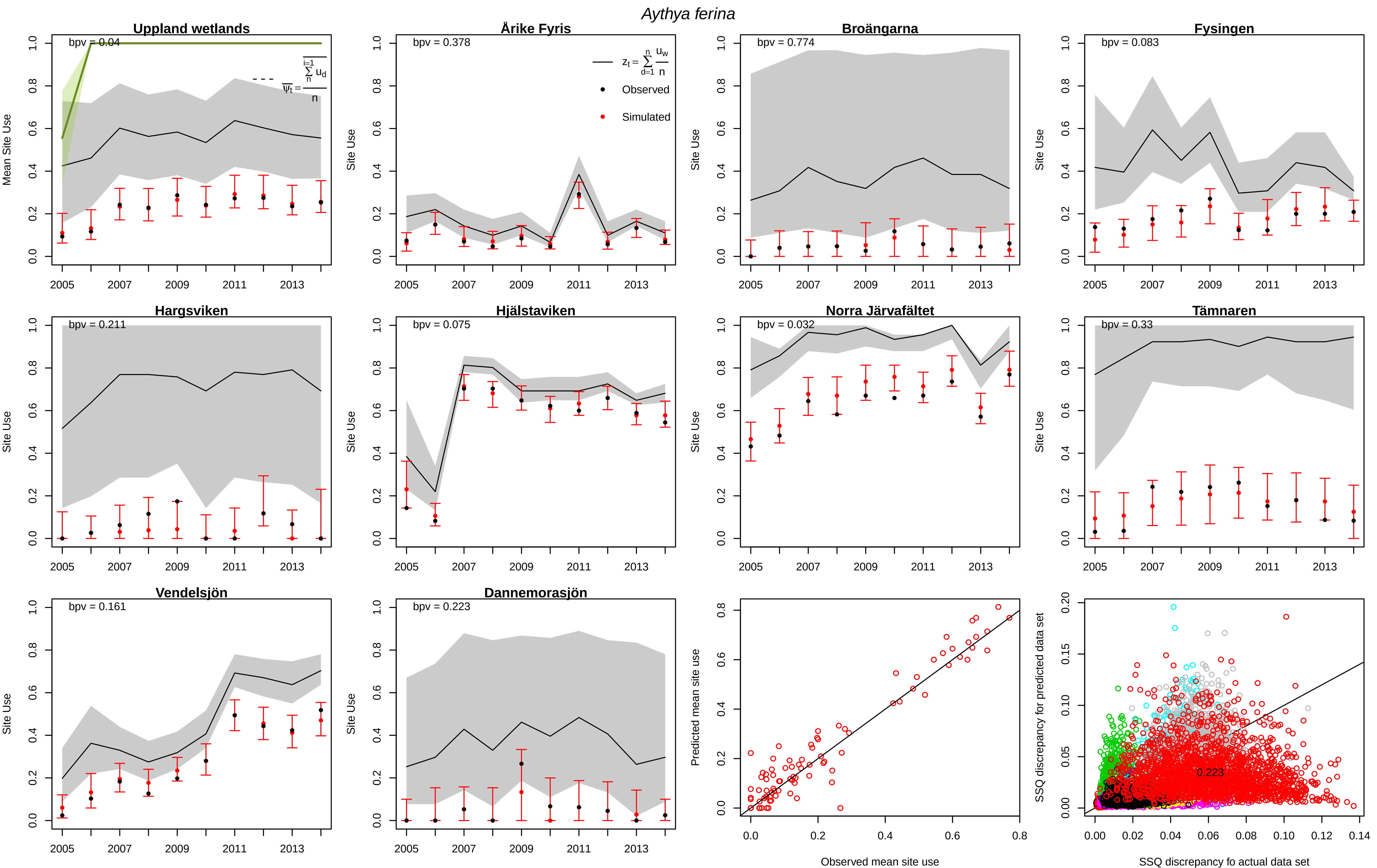

Aythya ferina
Uppland wetlands
bpv = 0.04
Mean Site Use
Årike Fyris
bpv = 0.378
Observed
Simulated
Broängarna
bpv = 0.774
Fysingen
bpv = 0.083
Hargsviken
bpv = 0.211
Hjälstaviken
bpv = 0.075
Norra Järvafältet
bpv = 0.032
Tämnaren
bpv = 0.33
Vendelsjön
bpv = 0.161
Dannemorasjön
bpv = 0.223
Site Use
Predicted mean site use
Observed mean site use
SSQ discrepancy for predicted data set
SSQ discrepancy fo actual data set
0.223

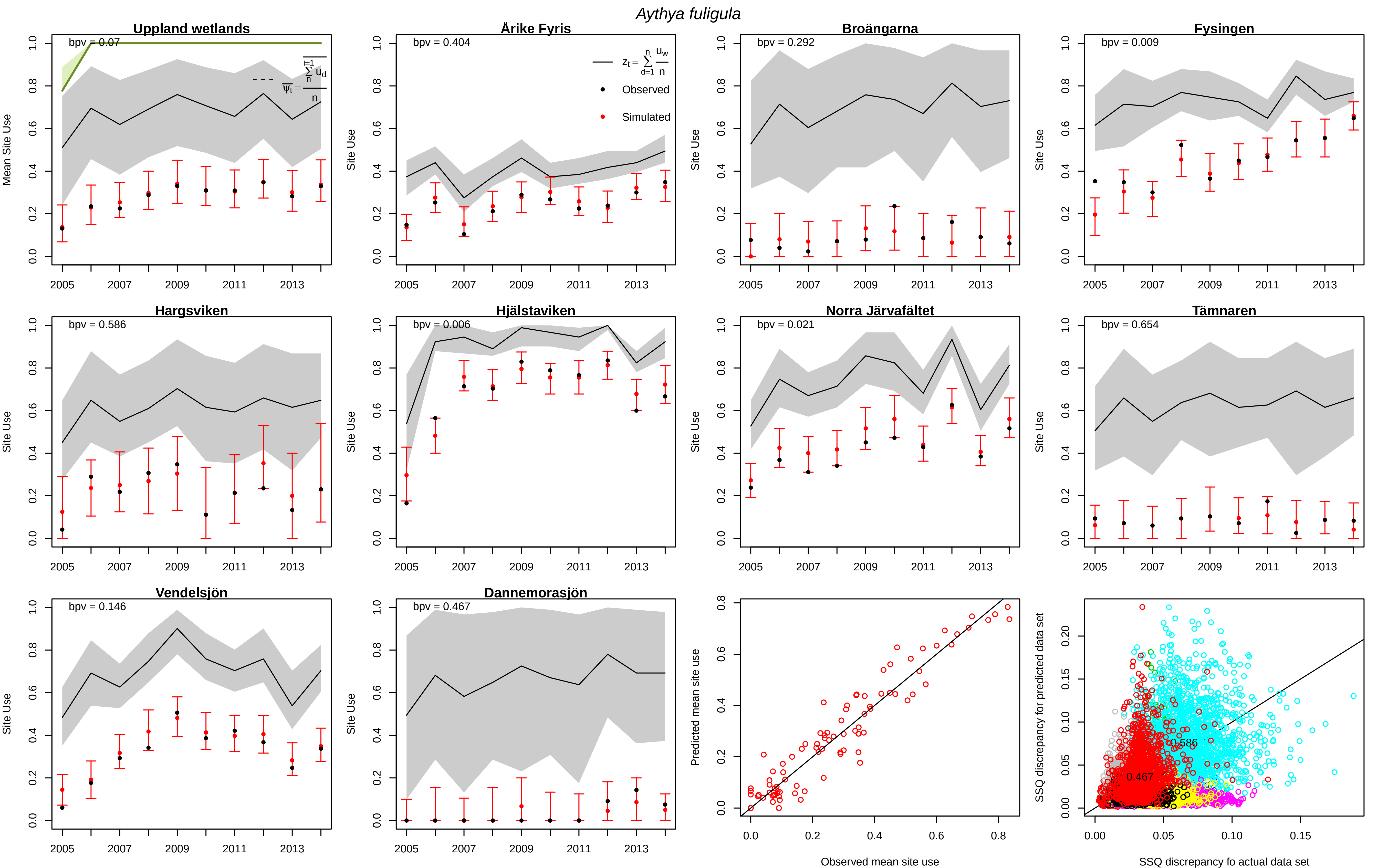

Aythya fuligula
Uppland wetlands
bpv = 0.07
Mean Site Use
Årike Fyris
bpv = 0.404
Observed
Simulated
Broängarna
bpv = 0.292
Fysingen
bpv = 0.009
Hargsviken
bpv = 0.586
Hjälstaviken
bpv = 0.006
Norra Järvafältet
bpv = 0.021
Tämnaren
bpv = 0.654
Vendelsjön
bpv = 0.146
Dannemorasjön
bpv = 0.467
Site Use
Predicted mean site use
Observed mean site use
SSQ discrepancy for predicted data set
SSQ discrepancy fo actual data set

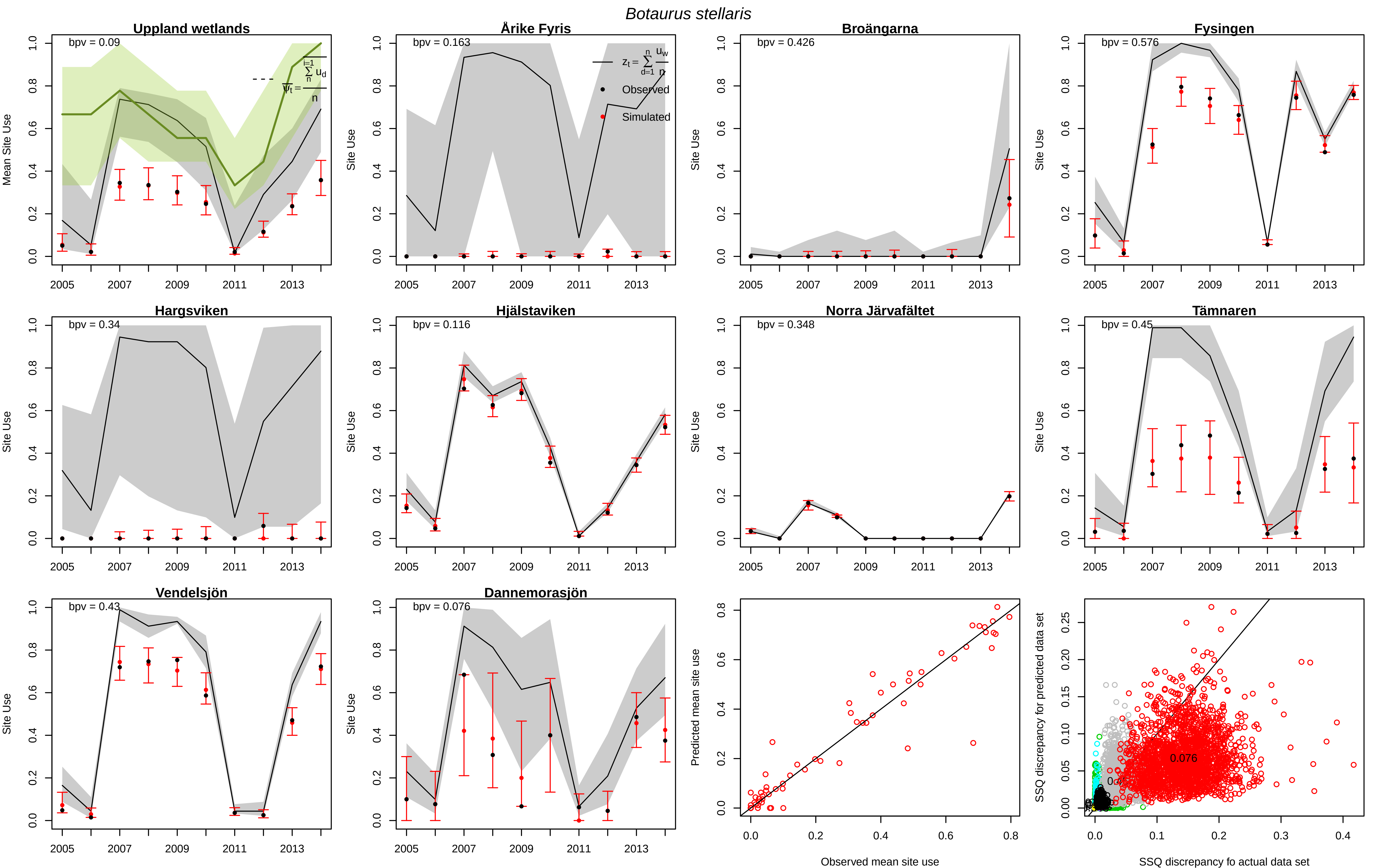

Botaurus stellaris
Uppland wetlands
bpv = 0.09
Mean Site Use
Årike Fyris
bpv = 0.163
Observed
Simulated
Broängarna
bpv = 0.426
Fysingen
bpv = 0.576
Hargsviken
bpv = 0.34
Hjälstaviken
bpv = 0.116
Norra Järvafältet
bpv = 0.348
Tämnaren
bpv = 0.45
Vendelsjön
bpv = 0.43
Dannemorasjön
bpv = 0.076
Site Use
Predicted mean site use
Observed mean site use
SSQ discrepancy for predicted data set
SSQ discrepancy fo actual data set
0.076

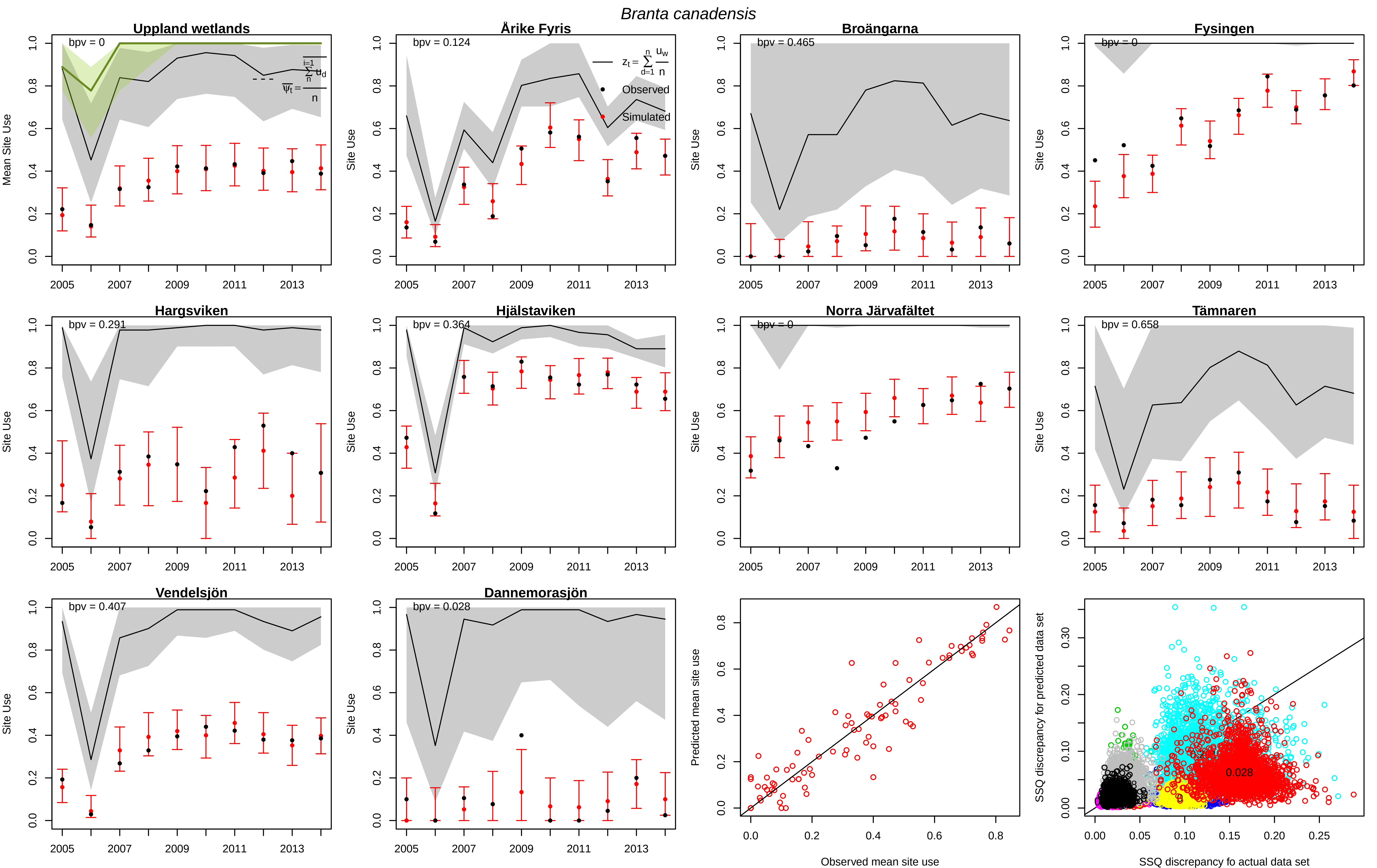

Branta canadensis
Uppland wetlands
Årike Fyris
Broängarna
Fysingen
Hargsviken
Hjälstaviken
Norra Järvafältet
Tämnaren
Vendelsjön
Dannemorasjön
bpv = 0
bpv = 0.124
bpv = 0.465
bpv = 0
bpv = 0.291
bpv = 0.364
bpv = 0
bpv = 0.658
bpv = 0.407
bpv = 0.028
Observed
Simulated
Mean Site Use
Site Use
Predicted mean site use
Observed mean site use
SSQ discrepancy for predicted data set
SSQ discrepancy fo actual data set
0.028

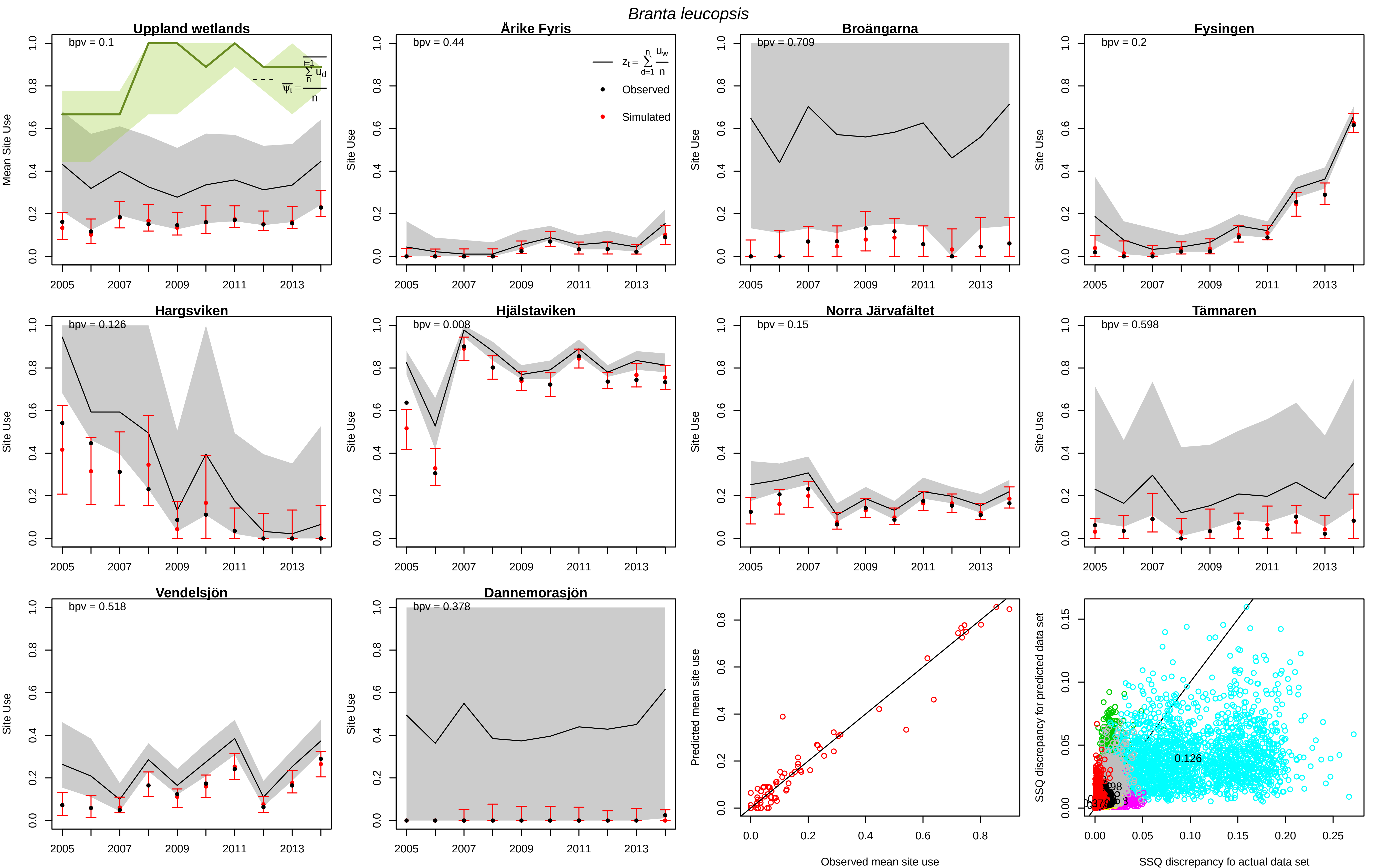

Branta leucopsis
Uppland wetlands
bpv = 0.1
Mean Site Use
Årike Fyris
bpv = 0.44
Observed
Simulated
Broängarna
bpv = 0.709
Fysingen
bpv = 0.2
Hargsviken
bpv = 0.126
Hjälstaviken
bpv = 0.008
Norra Järvafältet
bpv = 0.15
Tämnaren
bpv = 0.598
Vendelsjön
bpv = 0.518
Dannemorasjön
bpv = 0.378
Site Use
Predicted mean site use
Observed mean site use
SSQ discrepancy for predicted data set
SSQ discrepancy fo actual data set

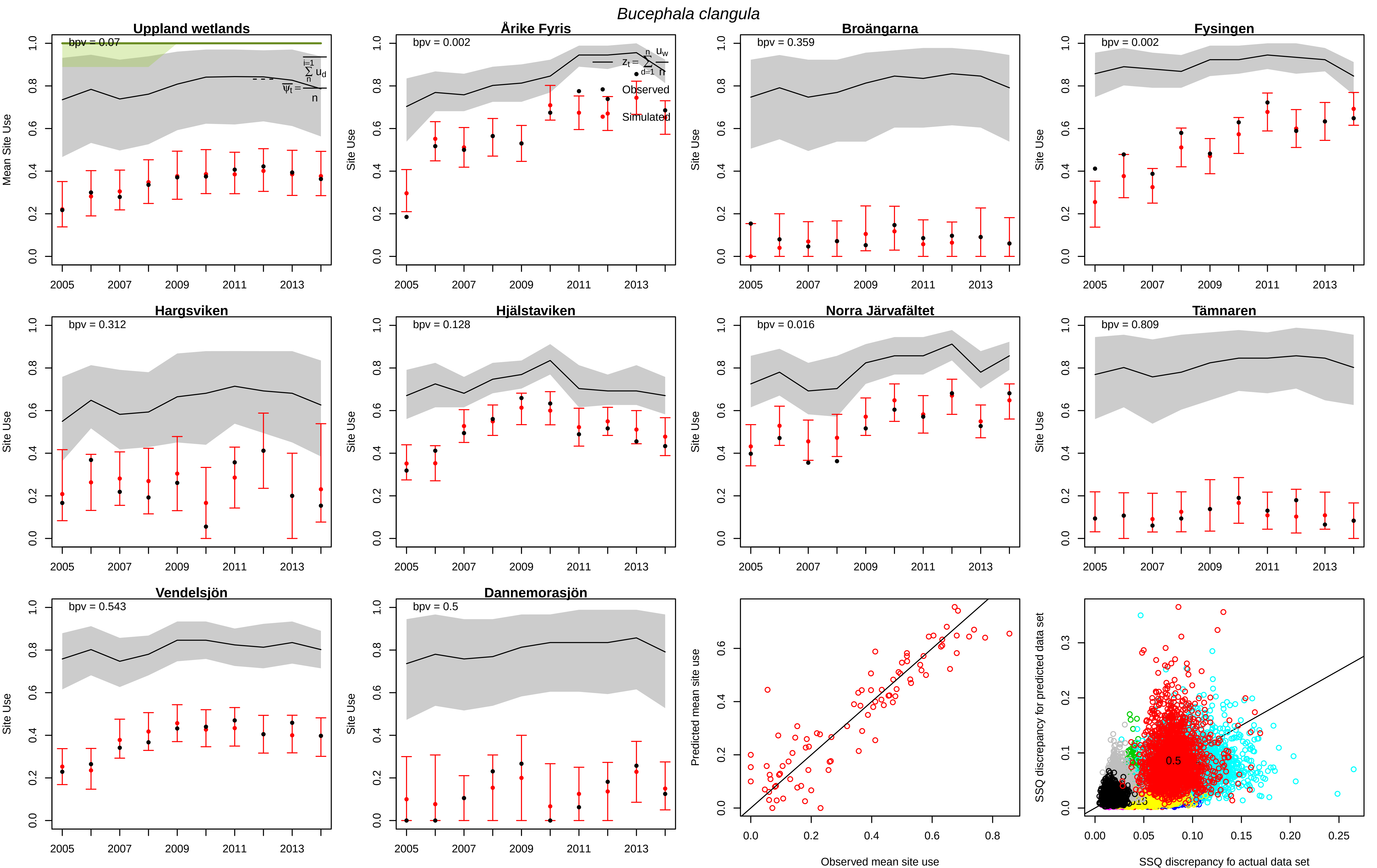

Bucephala clangula
Uppland wetlands
bpv = 0.07
Observed
Simulated
Årike Fyris
bpv = 0.002
Broängarna
bpv = 0.359
Fysingen
bpv = 0.002
Hargsviken
bpv = 0.312
Hjälstaviken
bpv = 0.128
Norra Järvafältet
bpv = 0.016
Tämnaren
bpv = 0.809
Vendelsjön
bpv = 0.543
Dannemorasjön
bpv = 0.5
Mean Site Use
Site Use
Predicted mean site use
Observed mean site use
SSQ discrepancy for predicted data set
SSQ discrepancy fo actual data set
0.5

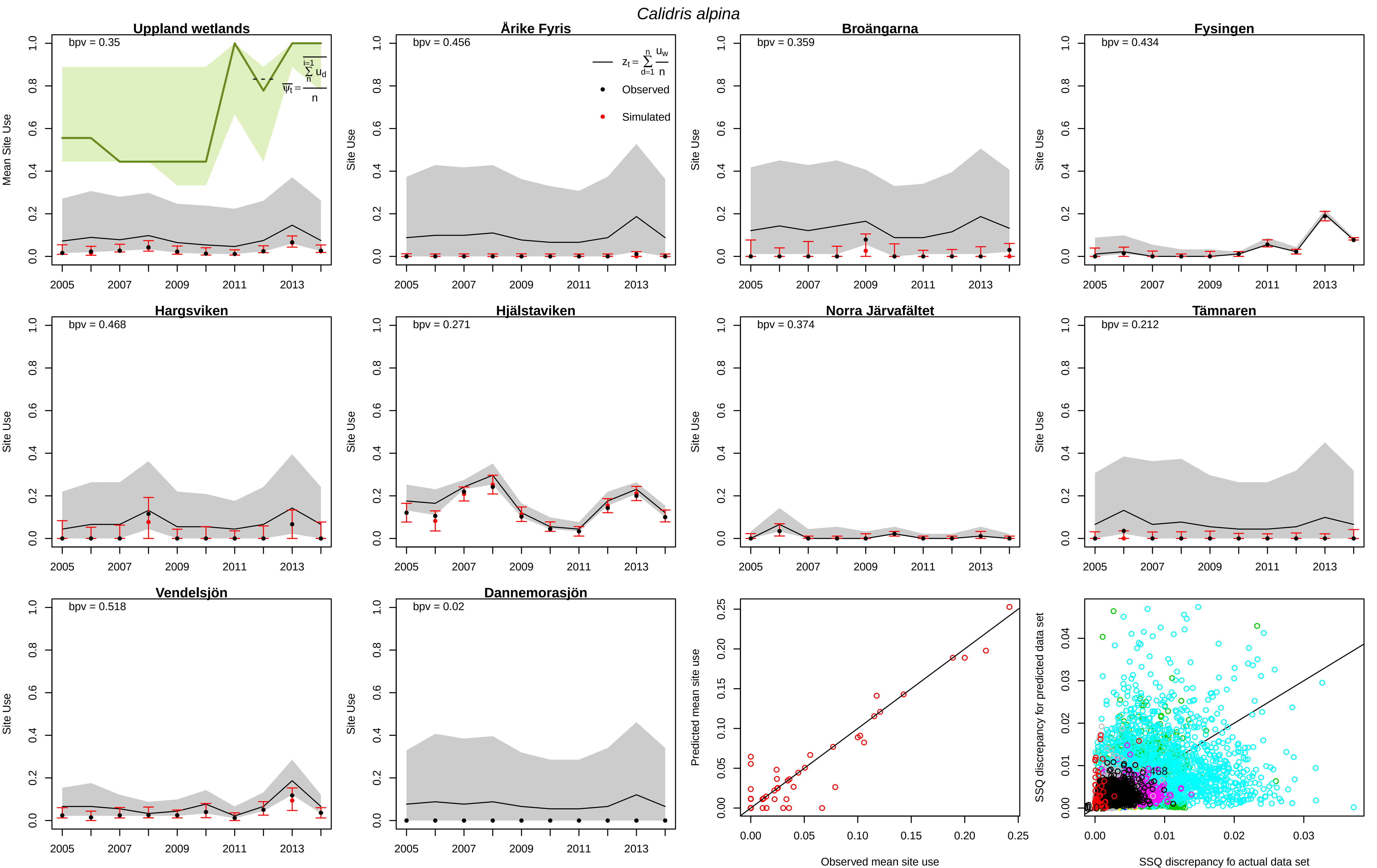

Calidris alpina
Uppland wetlands
bpv = 0.35
Mean Site Use
Årike Fyris
bpv = 0.456
Observed
Simulated
Broängarna
bpv = 0.359
Fysingen
bpv = 0.434
Hargsviken
bpv = 0.468
Hjälstaviken
bpv = 0.271
Norra Järvafältet
bpv = 0.374
Tämnaren
bpv = 0.212
Vendelsjön
bpv = 0.518
Dannemorasjön
bpv = 0.02
Site Use
Predicted mean site use
Observed mean site use
SSQ discrepancy for predicted data set
SSQ discrepancy fo actual data set

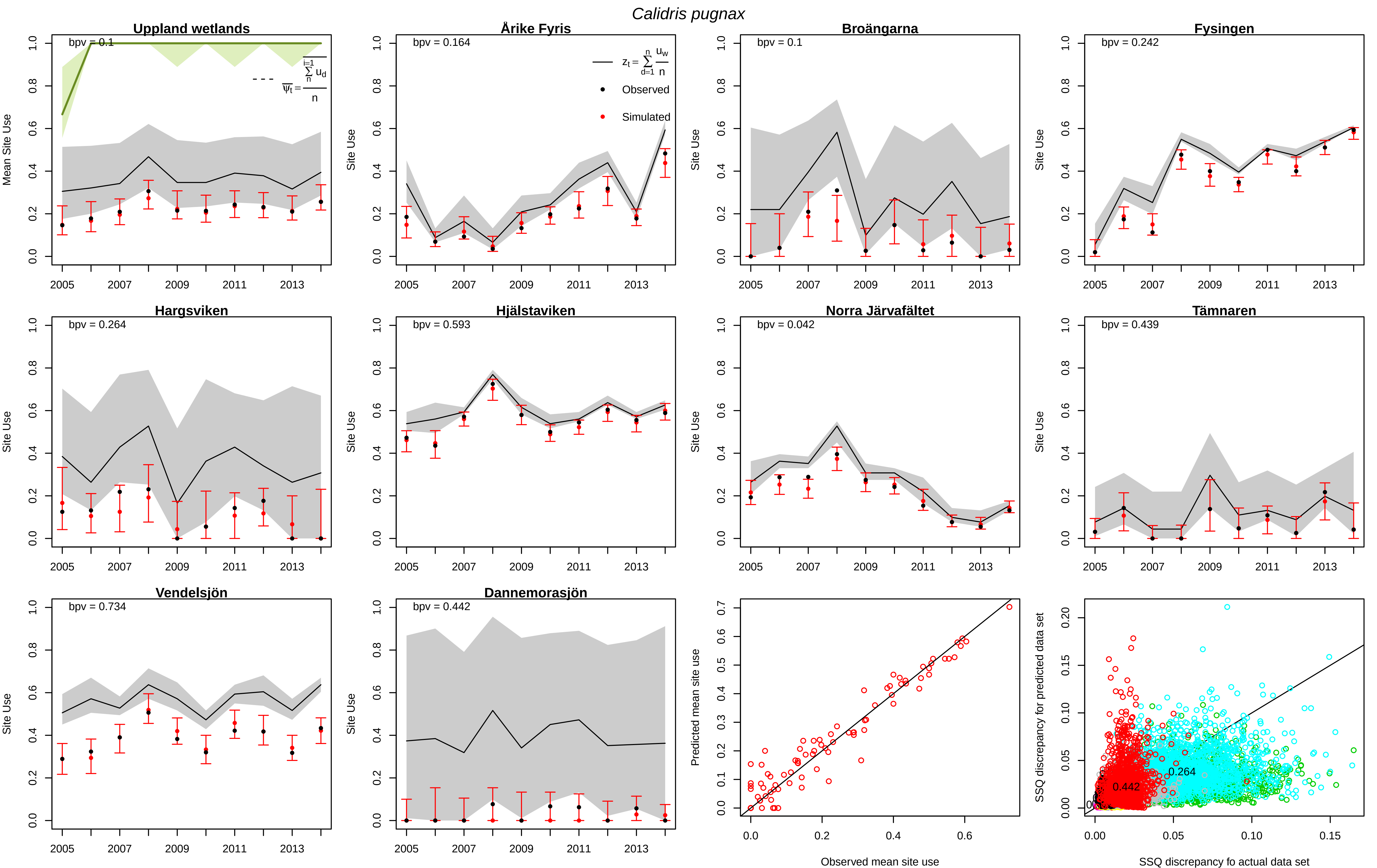

Calidris pugnax
Uppland wetlands
bpv = 0.1
Mean Site Use
Årike Fyris
bpv = 0.164
Observed
Simulated
Broängarna
bpv = 0.1
Fysingen
bpv = 0.242
Hargsviken
bpv = 0.264
Hjälstaviken
bpv = 0.593
Norra Järvafältet
bpv = 0.042
Tämnaren
bpv = 0.439
Vendelsjön
bpv = 0.734
Dannemorasjön
bpv = 0.442
Site Use
Predicted mean site use
Observed mean site use
SSQ discrepancy for predicted data set
SSQ discrepancy fo actual data set
0.264
0.442

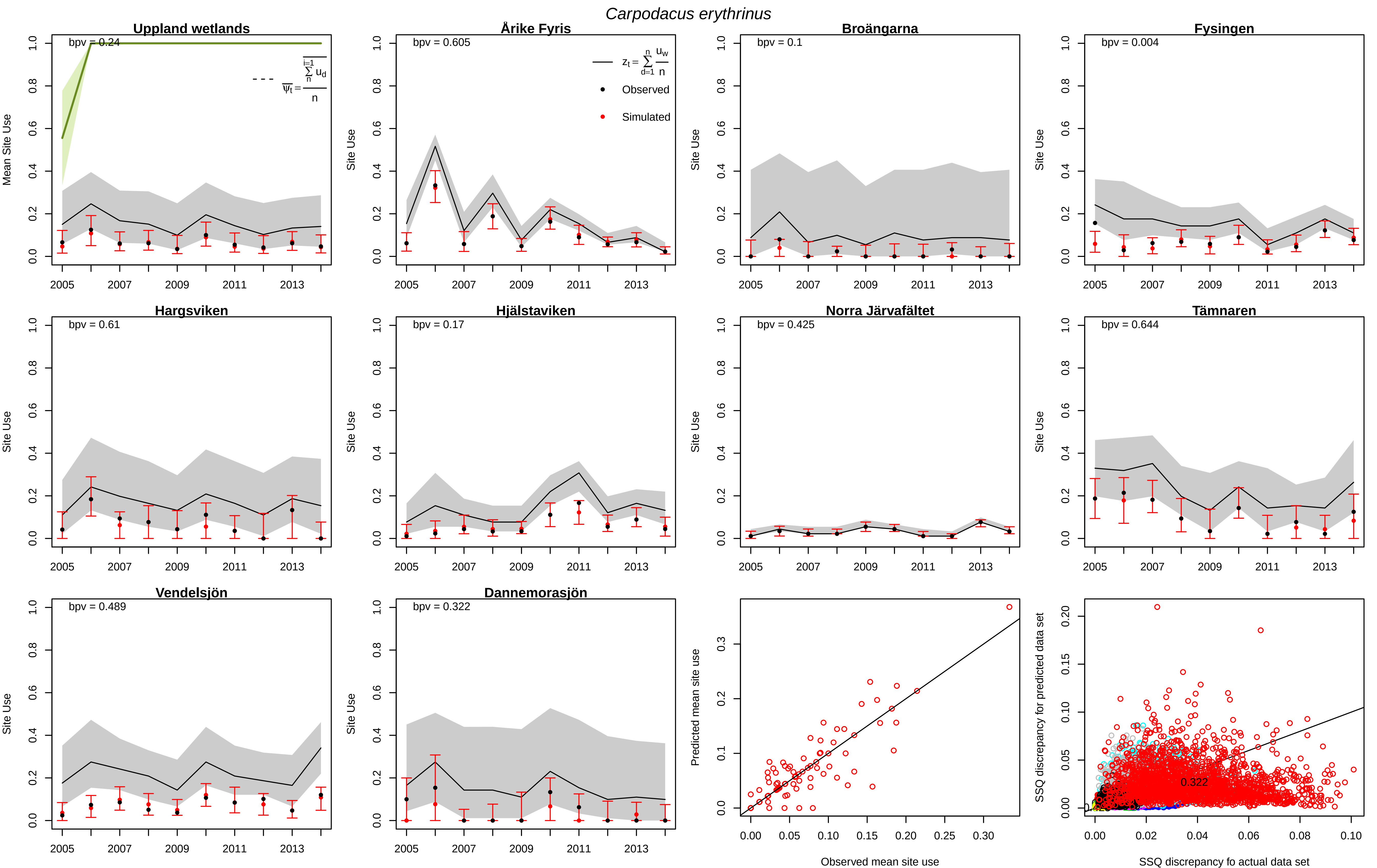

Carpodacus erythrinus
Uppland wetlands
bpv = 0.24
Mean Site Use
Årike Fyris
bpv = 0.605
Observed
Simulated
Broängarna
bpv = 0.1
Fysingen
bpv = 0.004
Hargsviken
bpv = 0.61
Hjälstaviken
bpv = 0.17
Norra Järvafältet
bpv = 0.425
Tämnaren
bpv = 0.644
Vendelsjön
bpv = 0.489
Dannemorasjön
bpv = 0.322
Site Use
Predicted mean site use
Observed mean site use
SSQ discrepancy for predicted data set
SSQ discrepancy fo actual data set
0.322

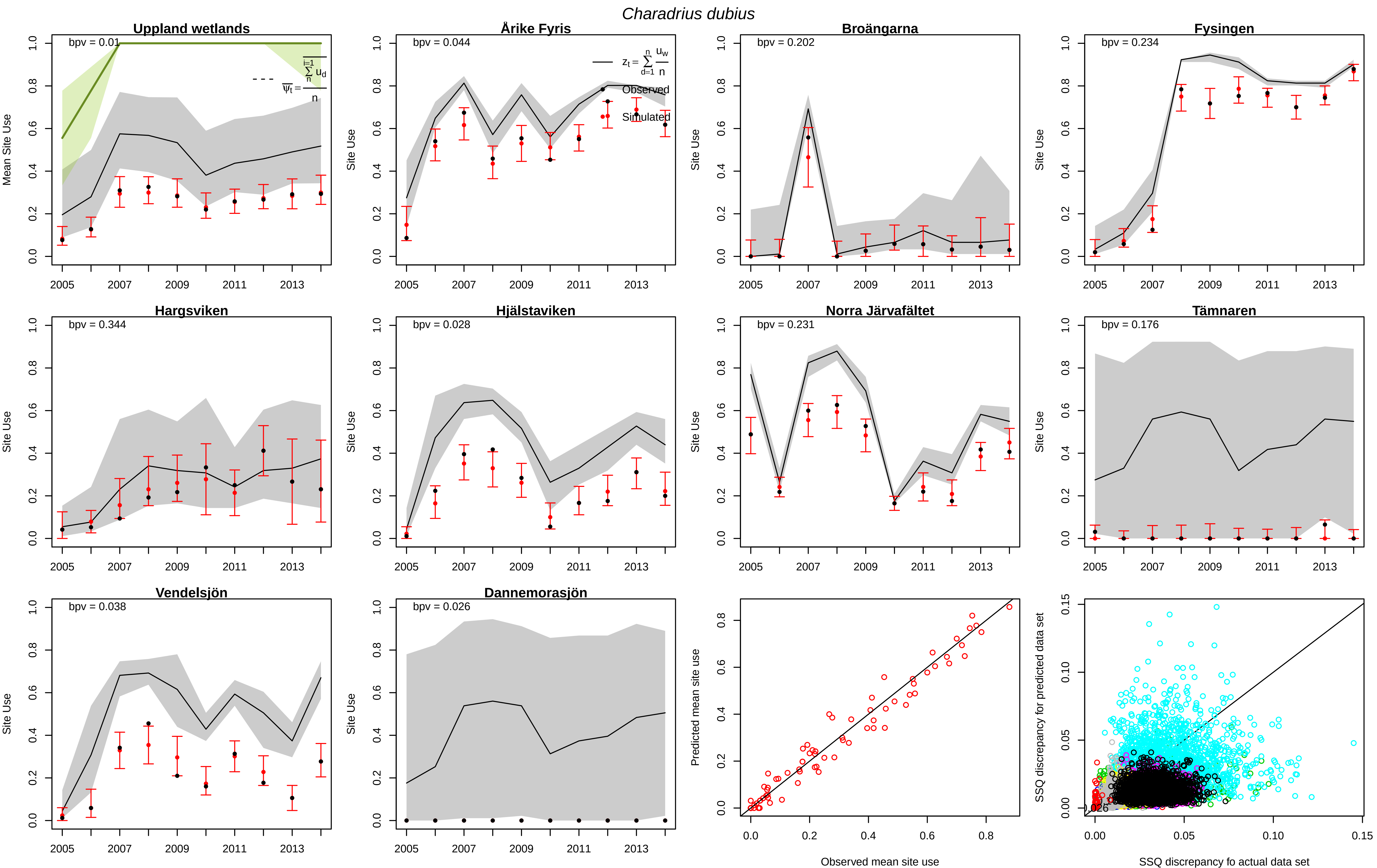

Charadrius dubius
Uppland wetlands
bpv = 0.01
Mean Site Use
Årike Fyris
bpv = 0.044
Observed
Simulated
Broängarna
bpv = 0.202
Fysingen
bpv = 0.234
Hargsviken
bpv = 0.344
Hjälstaviken
bpv = 0.028
Norra Järvafältet
bpv = 0.231
Tämnaren
bpv = 0.176
Vendelsjön
bpv = 0.038
Dannemorasjön
bpv = 0.026
Site Use
Predicted mean site use
Observed mean site use
SSQ discrepancy for predicted data set
SSQ discrepancy fo actual data set

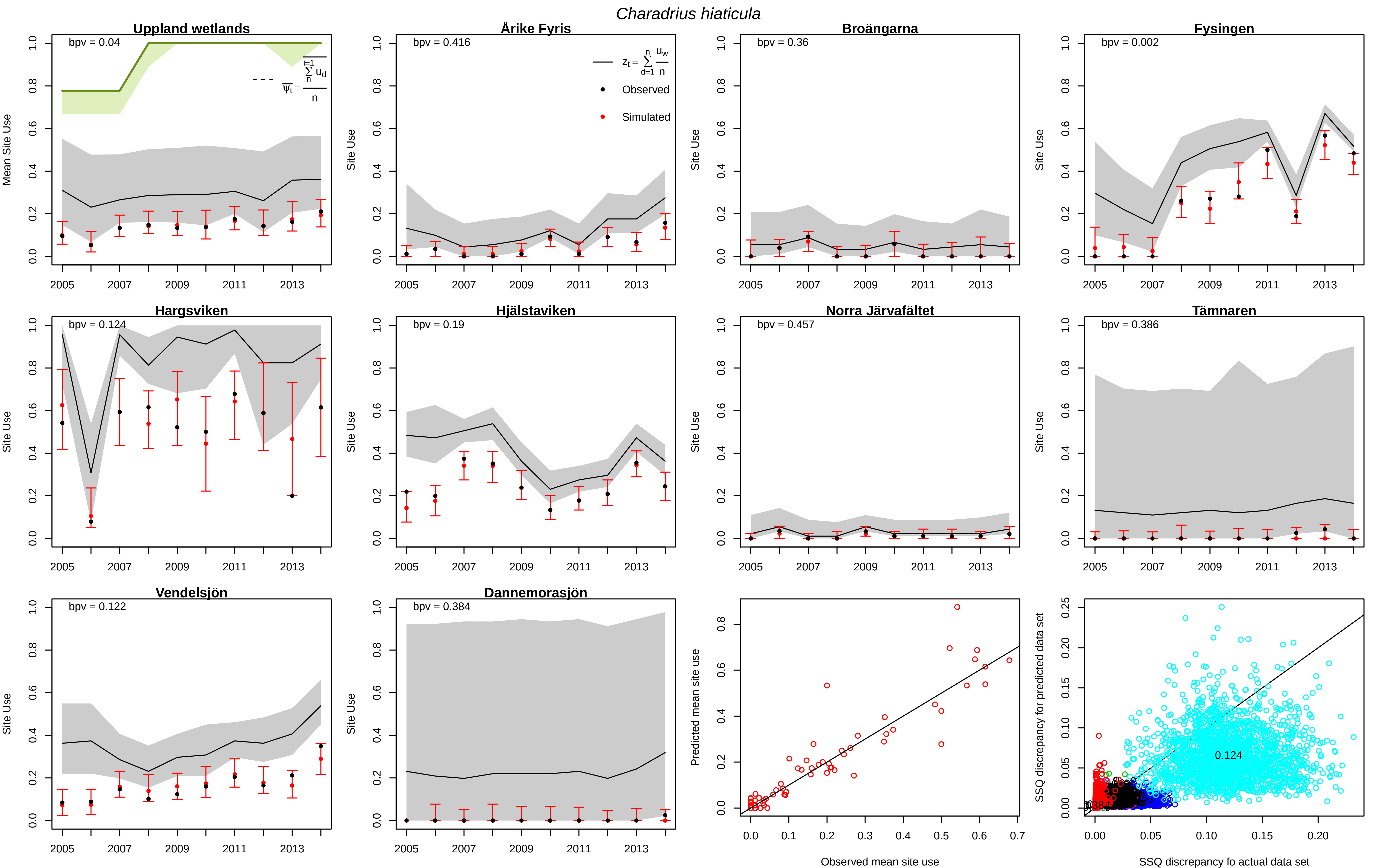

Charadrius hiaticula
Uppland wetlands
bpv = 0.04
Mean Site Use
Årike Fyris
bpv = 0.416
Observed
Simulated
Broängarna
bpv = 0.36
Fysingen
bpv = 0.002
Hargsviken
bpv = 0.124
Hjälstaviken
bpv = 0.19
Norra Järvafältet
bpv = 0.457
Tämnaren
bpv = 0.386
Vendelsjön
bpv = 0.122
Dannemorasjön
bpv = 0.384
Site Use
Predicted mean site use
Observed mean site use
SSQ discrepancy for predicted data set
SSQ discrepancy fo actual data set
0.124

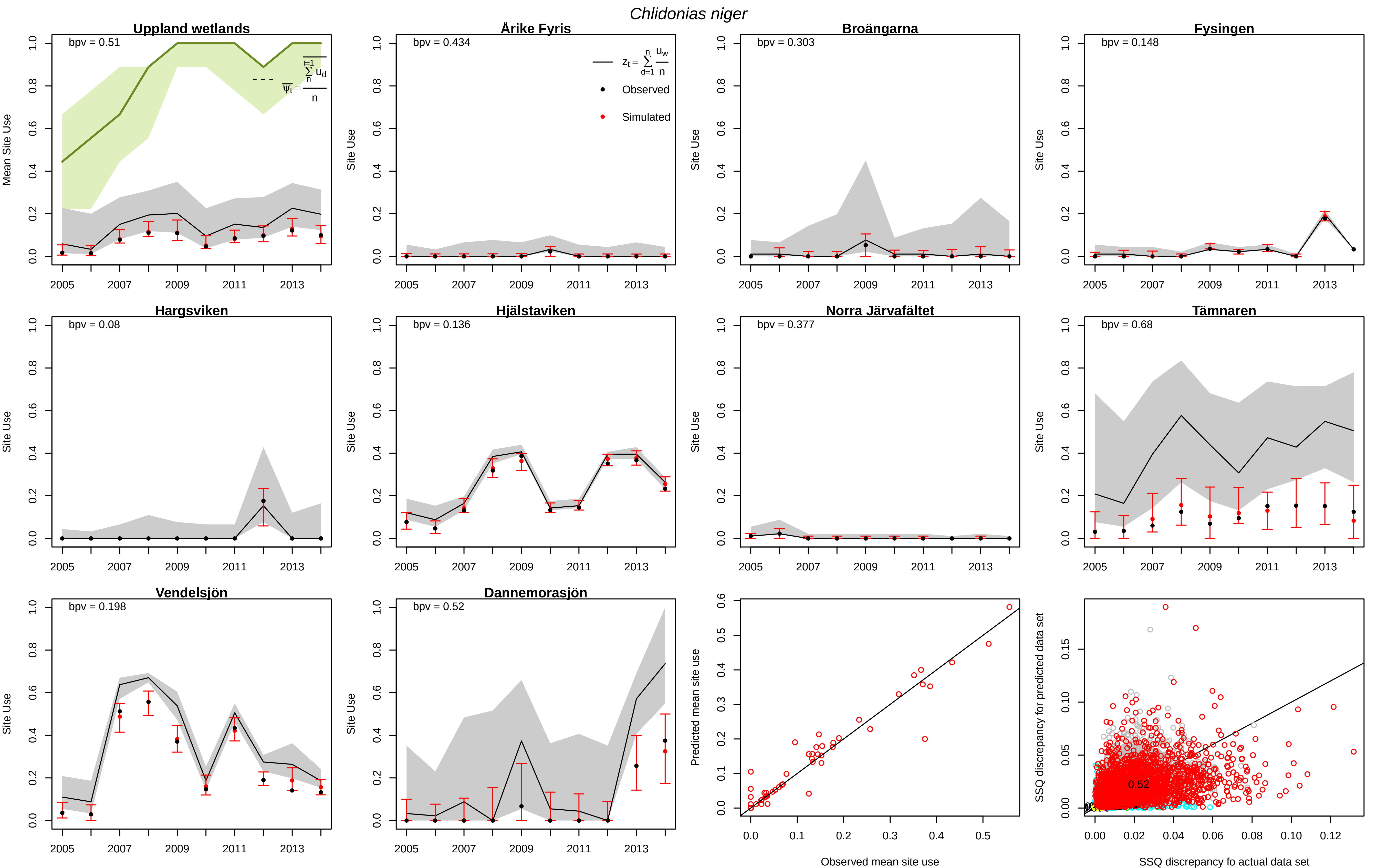
Chlidonias niger
Uppland wetlands
bpv = 0.51
Mean Site Use
Årike Fyris
bpv = 0.434
Observed
Simulated
Broängarna
bpv = 0.303
Fysingen
bpv = 0.148
Hargsviken
bpv = 0.08
Hjälstaviken
bpv = 0.136
Norra Järvafältet
bpv = 0.377
Tämnaren
bpv = 0.68
Vendelsjön
bpv = 0.198
Dannemorasjön
bpv = 0.52
Site Use
Predicted mean site use
Observed mean site use
SSQ discrepancy for predicted data set
SSQ discrepancy fo actual data set
0.52

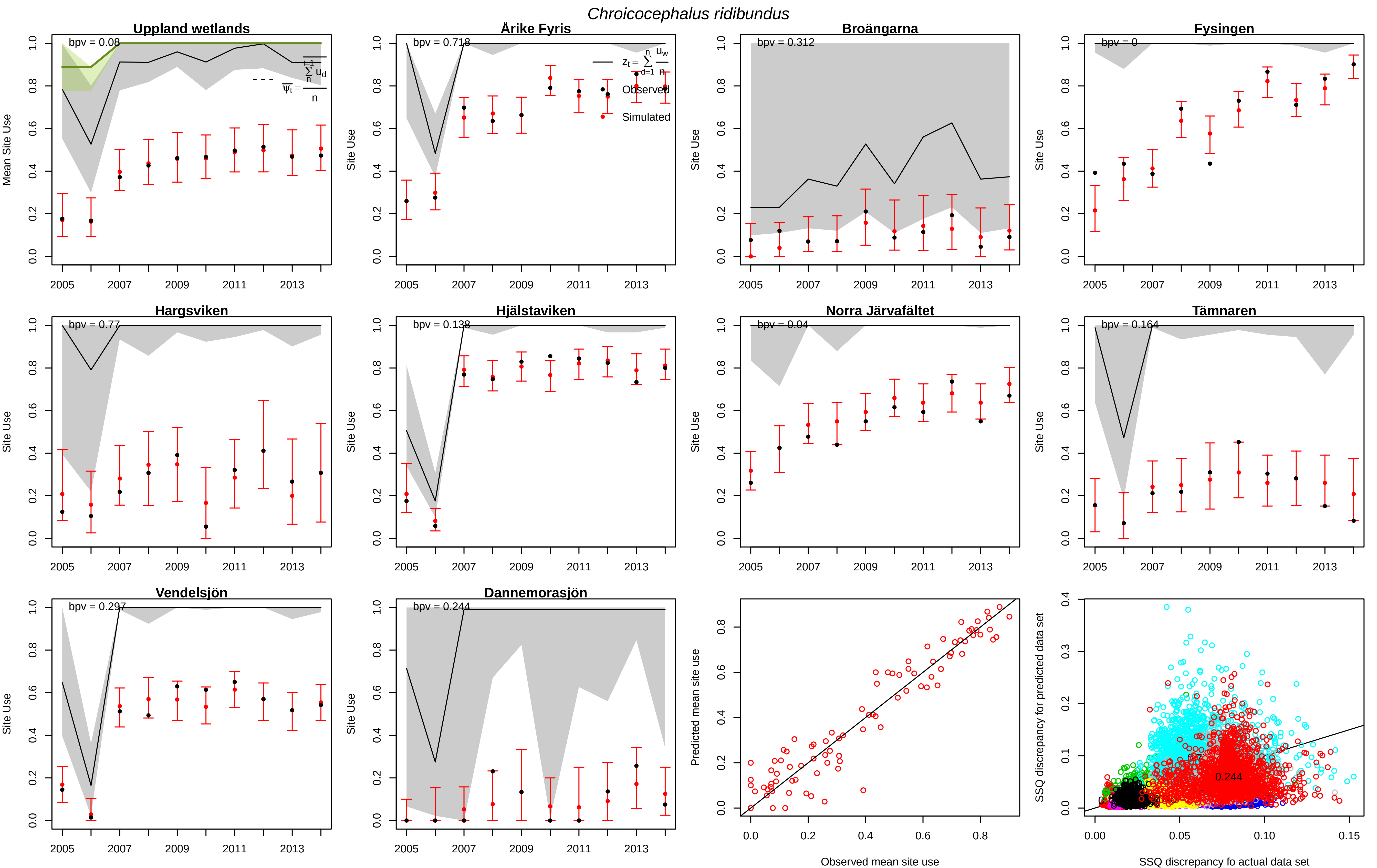
Chroicocephalus ridibundus
Uppland wetlands
bpv = 0.08
Mean Site Use
Årike Fyris
bpv = 0.718
Observed
Simulated
Broängarna
bpv = 0.312
Fysingen
bpv = 0
Hargsviken
bpv = 0.77
Hjälstaviken
bpv = 0.138
Norra Järvafältet
bpv = 0.04
Tämnaren
bpv = 0.164
Vendelsjön
bpv = 0.297
Dannemorasjön
bpv = 0.244
Site Use
Predicted mean site use
Observed mean site use
SSQ discrepancy for predicted data set
SSQ discrepancy fo actual data set
0.244

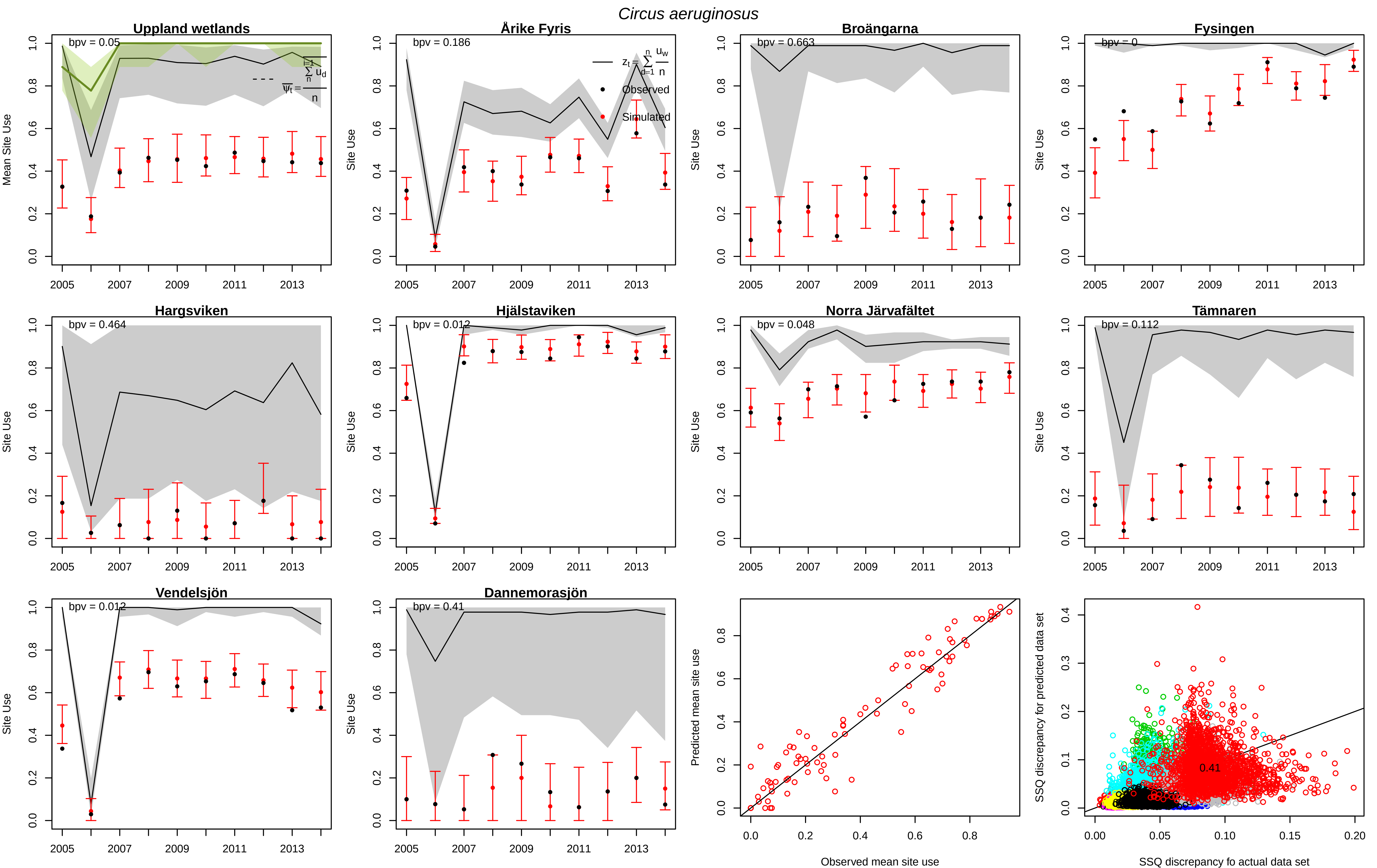
Circus aeruginosus
Uppland wetlands
bpv = 0.05
Mean Site Use
Årike Fyris
bpv = 0.186
Observed
Simulated
Broängarna
bpv = 0.663
Fysingen
bpv = 0
Hargsviken
bpv = 0.464
Hjälstaviken
bpv = 0.012
Norra Järvafältet
bpv = 0.048
Tämnaren
bpv = 0.112
Vendelsjön
bpv = 0.012
Dannemorasjön
bpv = 0.41
Site Use
2005
2007
2009
2011
2013
Predicted mean site use
Observed mean site use
0.41
SSQ discrepancy for predicted data set
SSQ discrepancy fo actual data set

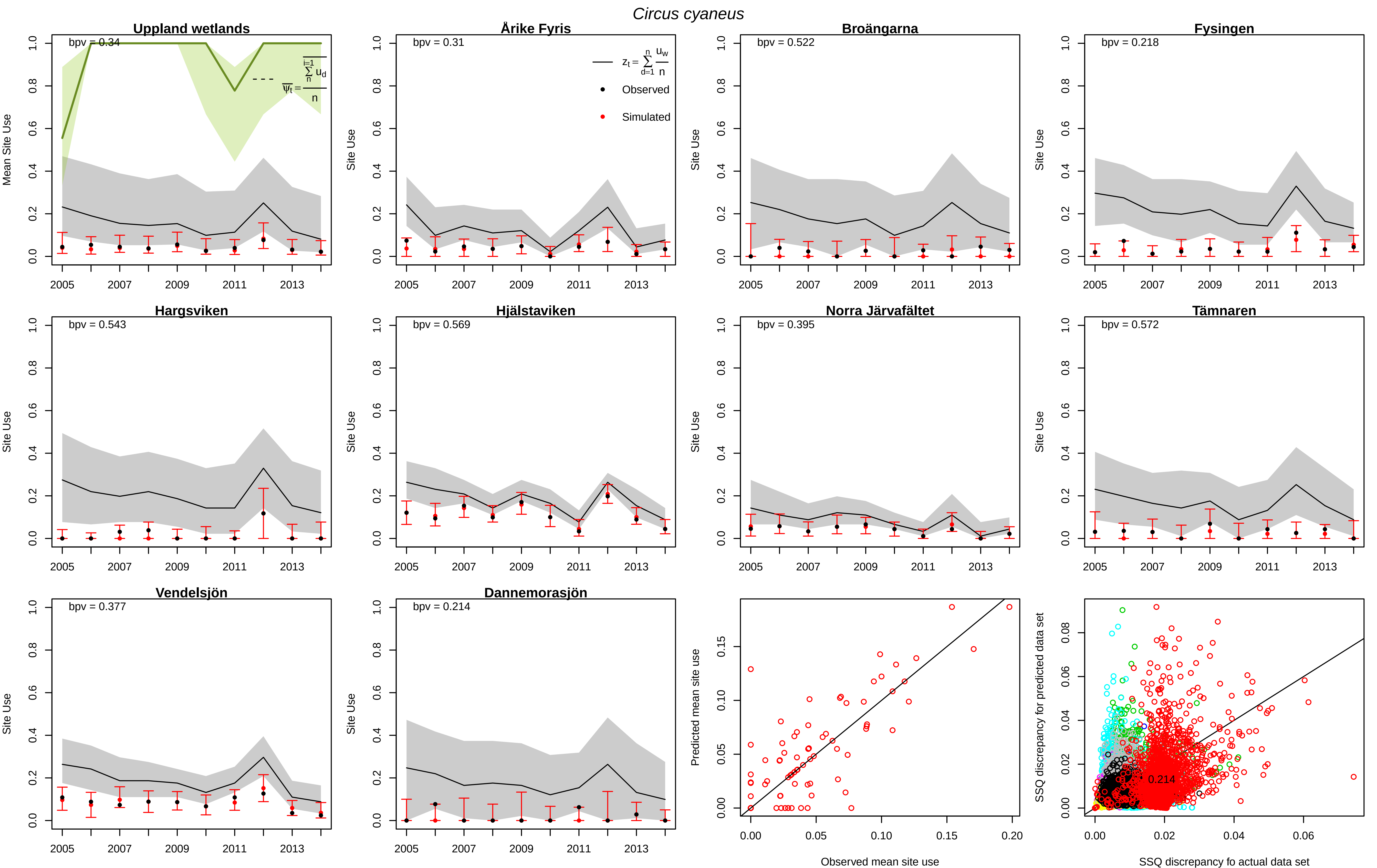

Circus cyaneus
Uppland wetlands
bpv = 0.34
Mean Site Use
Årike Fyris
bpv = 0.31
Observed
Simulated
Broängarna
bpv = 0.522
Fysingen
bpv = 0.218
Hargsviken
bpv = 0.543
Hjälstaviken
bpv = 0.569
Norra Järvafältet
bpv = 0.395
Tämnaren
bpv = 0.572
Vendelsjön
bpv = 0.377
Dannemorasjön
bpv = 0.214
Site Use
Predicted mean site use
Observed mean site use
SSQ discrepancy for predicted data set
SSQ discrepancy fo actual data set
0.214

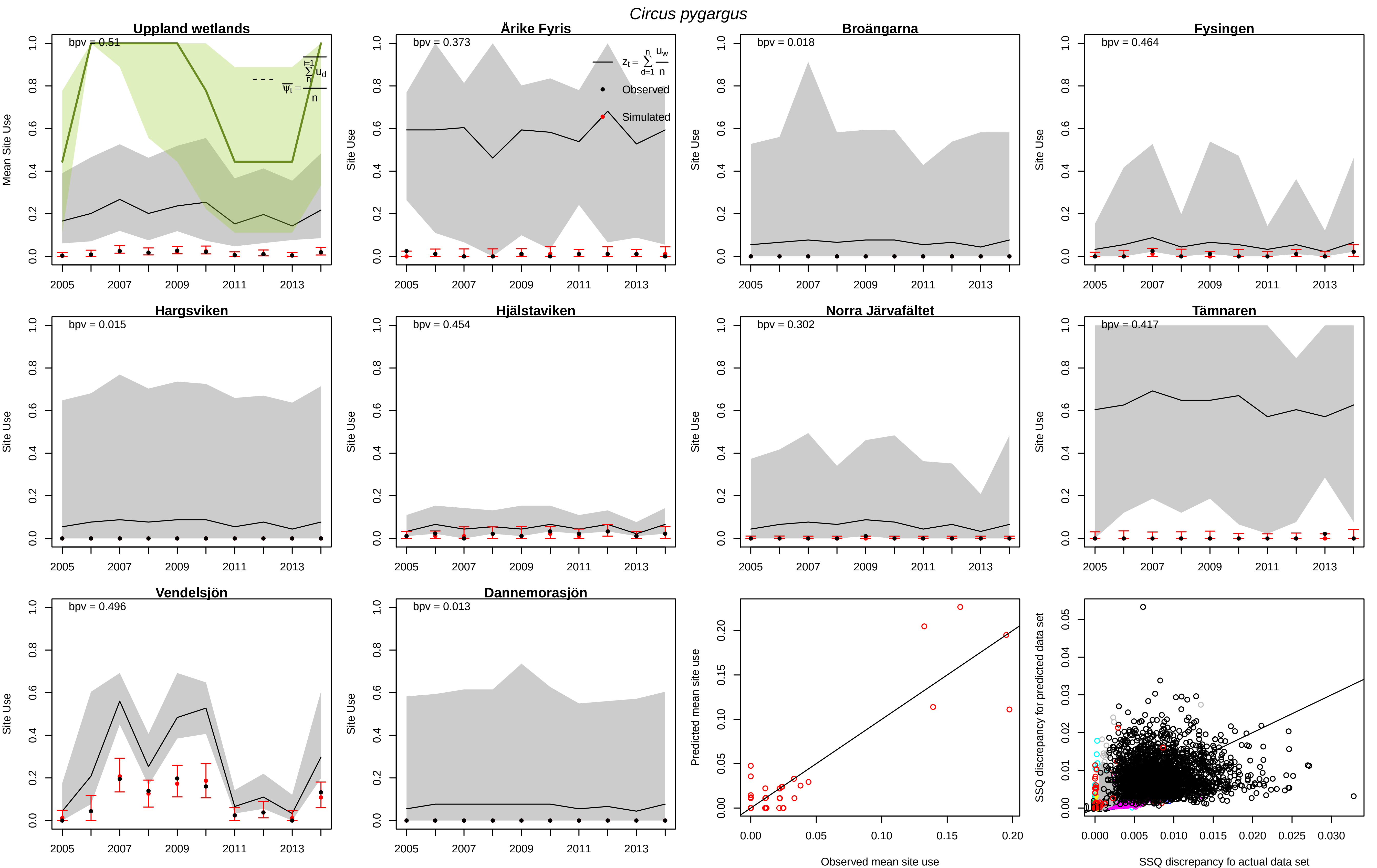

Circus pygargus
Uppland wetlands
bpv = 0.51
Mean Site Use
Årike Fyris
bpv = 0.373
Observed
Simulated
Broängarna
bpv = 0.018
Fysingen
bpv = 0.464
Hargsviken
bpv = 0.015
Hjälstaviken
bpv = 0.454
Norra Järvafältet
bpv = 0.302
Tämnaren
bpv = 0.417
Vendelsjön
bpv = 0.496
Dannemorasjön
bpv = 0.013
Site Use
Predicted mean site use
Observed mean site use
SSQ discrepancy for predicted data set
SSQ discrepancy fo actual data set

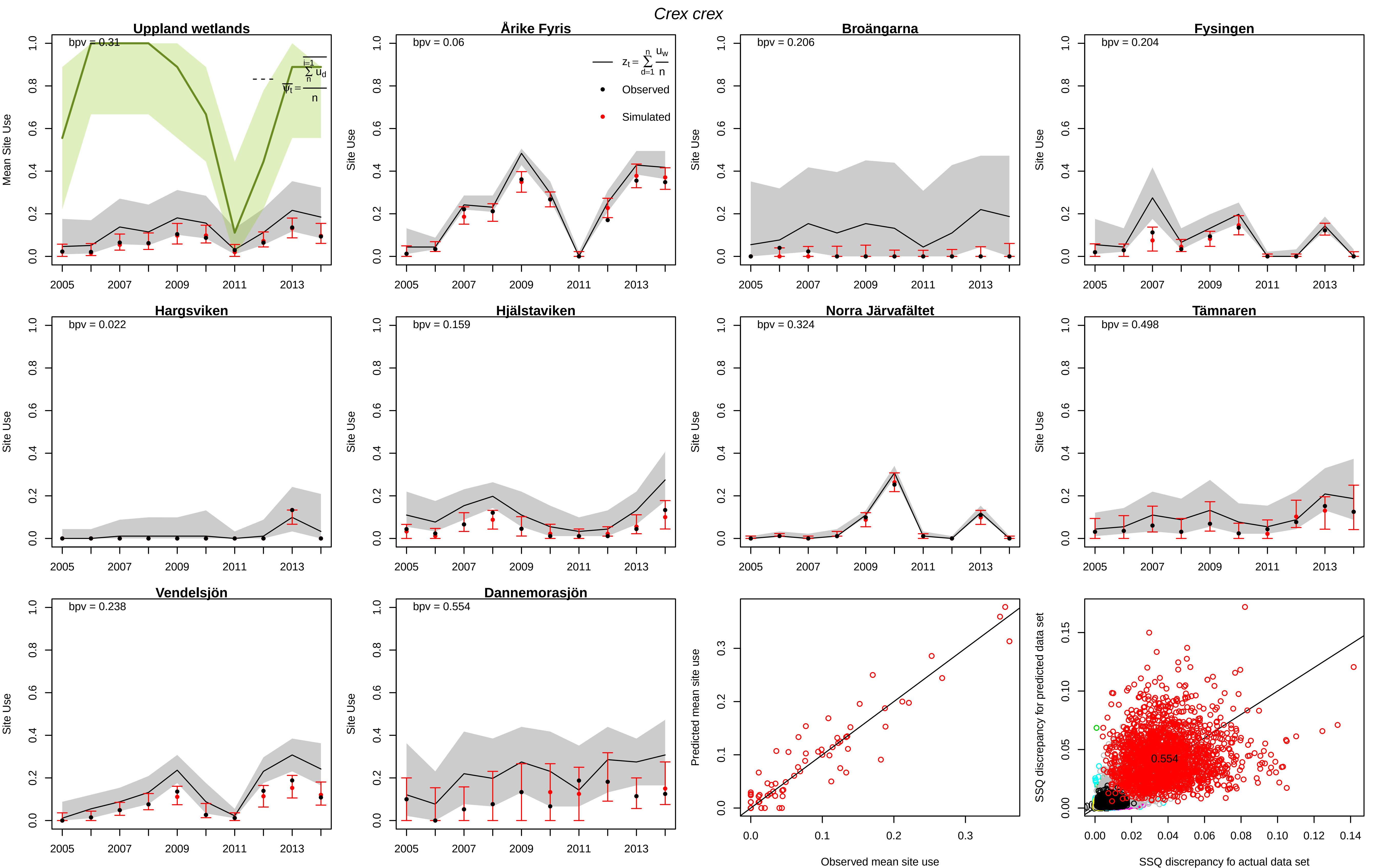
Crex crex
Uppland wetlands
bpv = 0.31
Mean Site Use
Årike Fyris
bpv = 0.06
Observed
Simulated
Broängarna
bpv = 0.206
Fysingen
bpv = 0.204
Hargsviken
bpv = 0.022
Hjälstaviken
bpv = 0.159
Norra Järvafältet
bpv = 0.324
Tämnaren
bpv = 0.498
Vendelsjön
bpv = 0.238
Dannemorasjön
bpv = 0.554
Site Use
Predicted mean site use
Observed mean site use
0.554
SSQ discrepancy for predicted data set
SSQ discrepancy fo actual data set

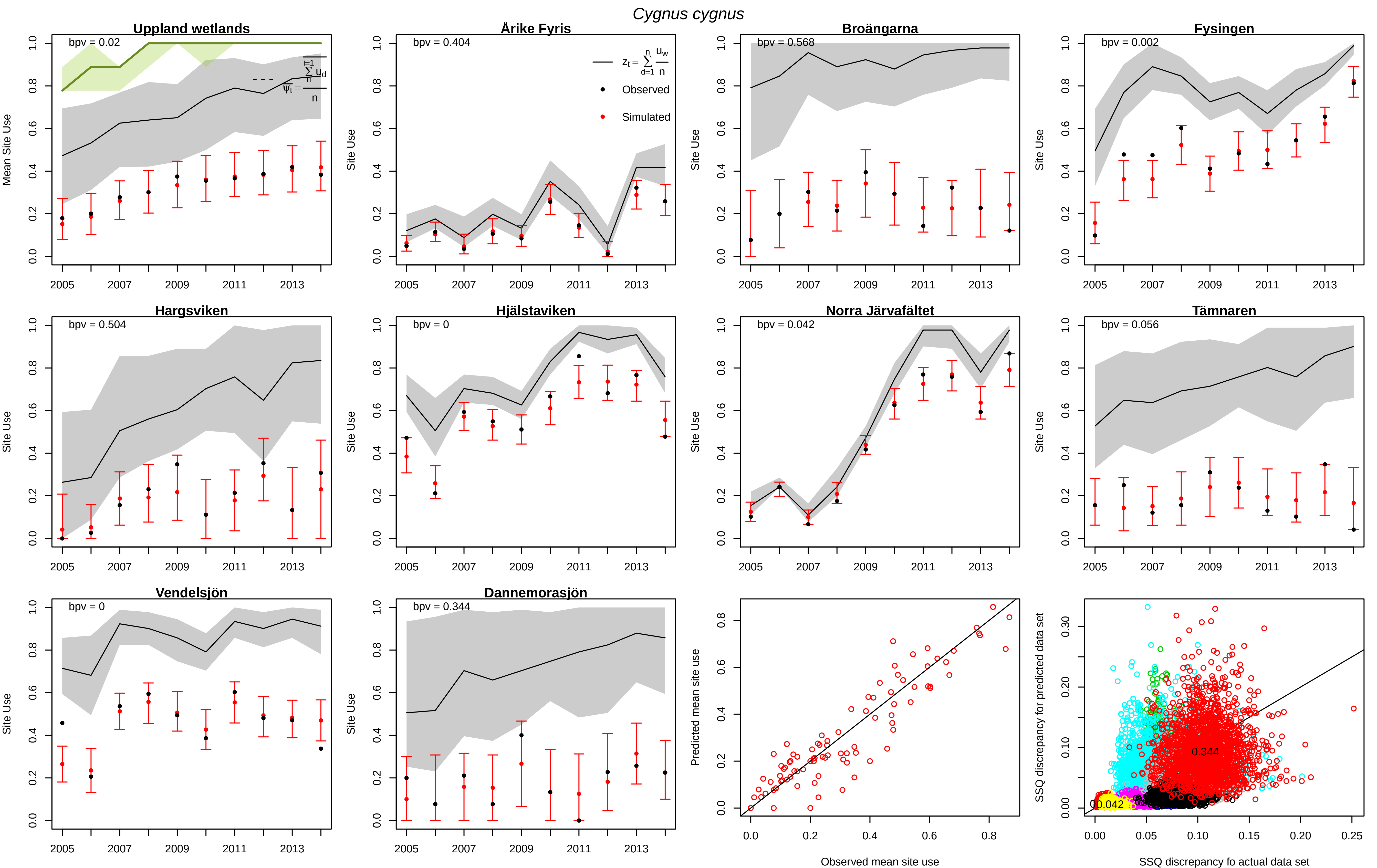

Cygnus cygnus
Uppland wetlands
bpv = 0.02
Mean Site Use
Årike Fyris
bpv = 0.404
Observed
Simulated
Broängarna
bpv = 0.568
Fysingen
bpv = 0.002
Hargsviken
bpv = 0.504
Hjälstaviken
bpv = 0
Norra Järvafältet
bpv = 0.042
Tämnaren
bpv = 0.056
Vendelsjön
bpv = 0
Dannemorasjön
bpv = 0.344
Site Use
Predicted mean site use
Observed mean site use
SSQ discrepancy for predicted data set
SSQ discrepancy fo actual data set
0.344
0.042

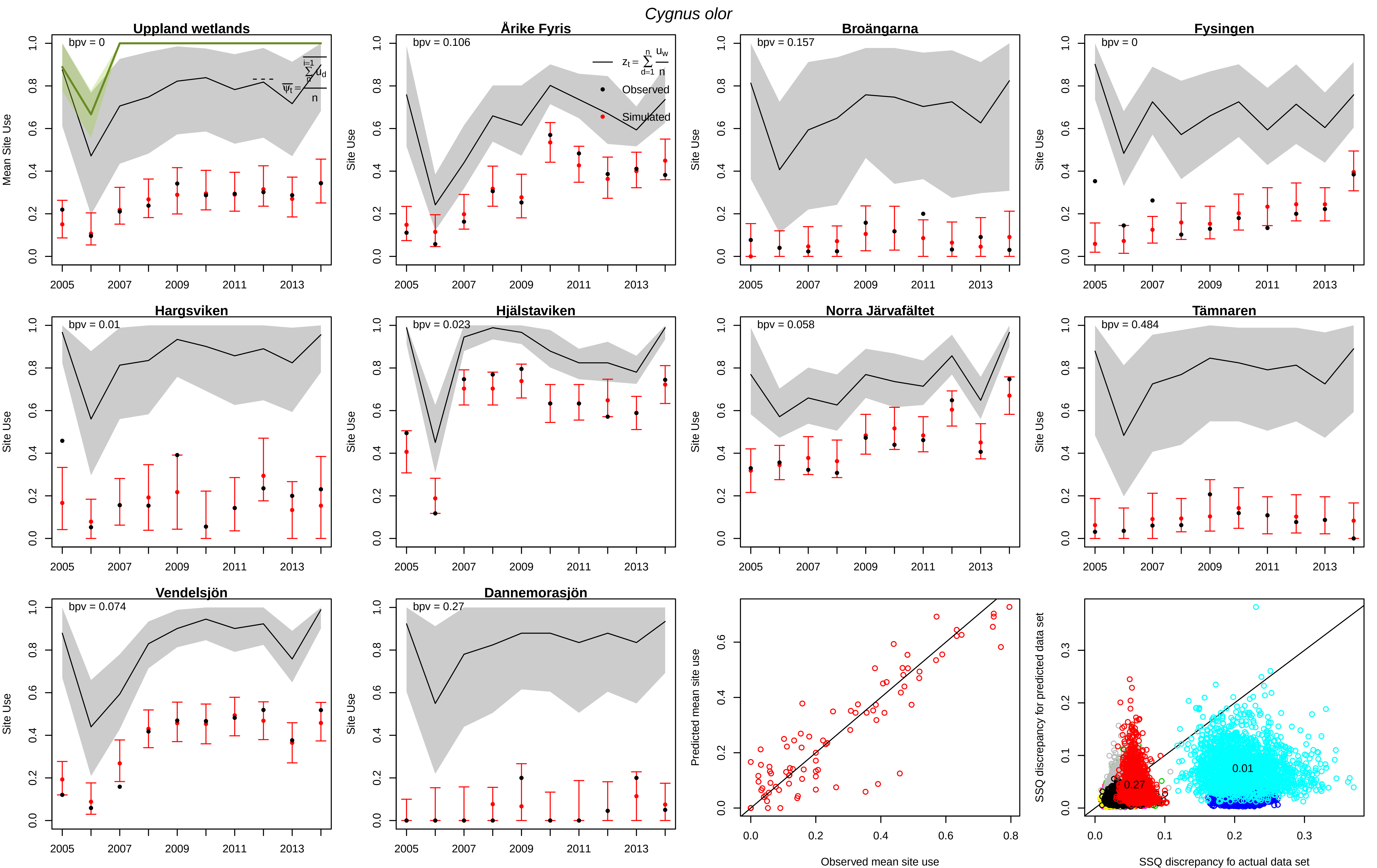
Cygnus olor
Uppland wetlands
bpv = 0
Mean Site Use
Årike Fyris
bpv = 0.106
Observed
Simulated
Broängarna
bpv = 0.157
Fysingen
bpv = 0
Hargsviken
bpv = 0.01
Hjälstaviken
bpv = 0.023
Norra Järvafältet
bpv = 0.058
Tämnaren
bpv = 0.484
Vendelsjön
bpv = 0.074
Dannemorasjön
bpv = 0.27
Site Use
Predicted mean site use
Observed mean site use
SSQ discrepancy for predicted data set
SSQ discrepancy fo actual data set
0.27
0.01

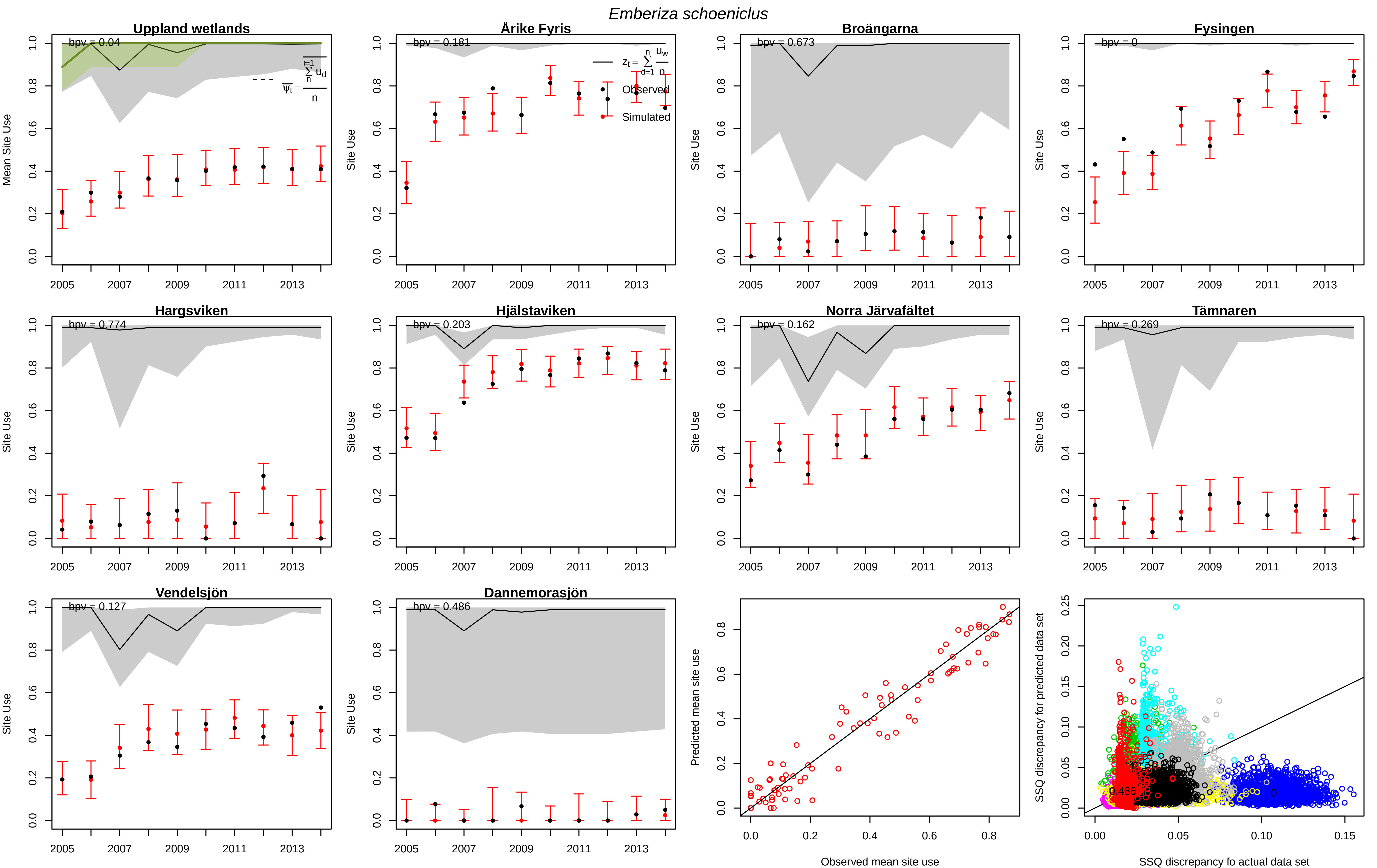

Emberiza schoeniclus
Uppland wetlands
bpv = 0.04
Mean Site Use
Årike Fyris
bpv = 0.181
Observed
Simulated
Broängarna
bpv = 0.673
Fysingen
bpv = 0
Hargsviken
bpv = 0.774
Hjälstaviken
bpv = 0.203
Norra Järvafältet
bpv = 0.162
Tämnaren
bpv = 0.269
Vendelsjön
bpv = 0.127
Dannemorasjön
bpv = 0.486
Site Use
Predicted mean site use
Observed mean site use
SSQ discrepancy for predicted data set
SSQ discrepancy fo actual data set

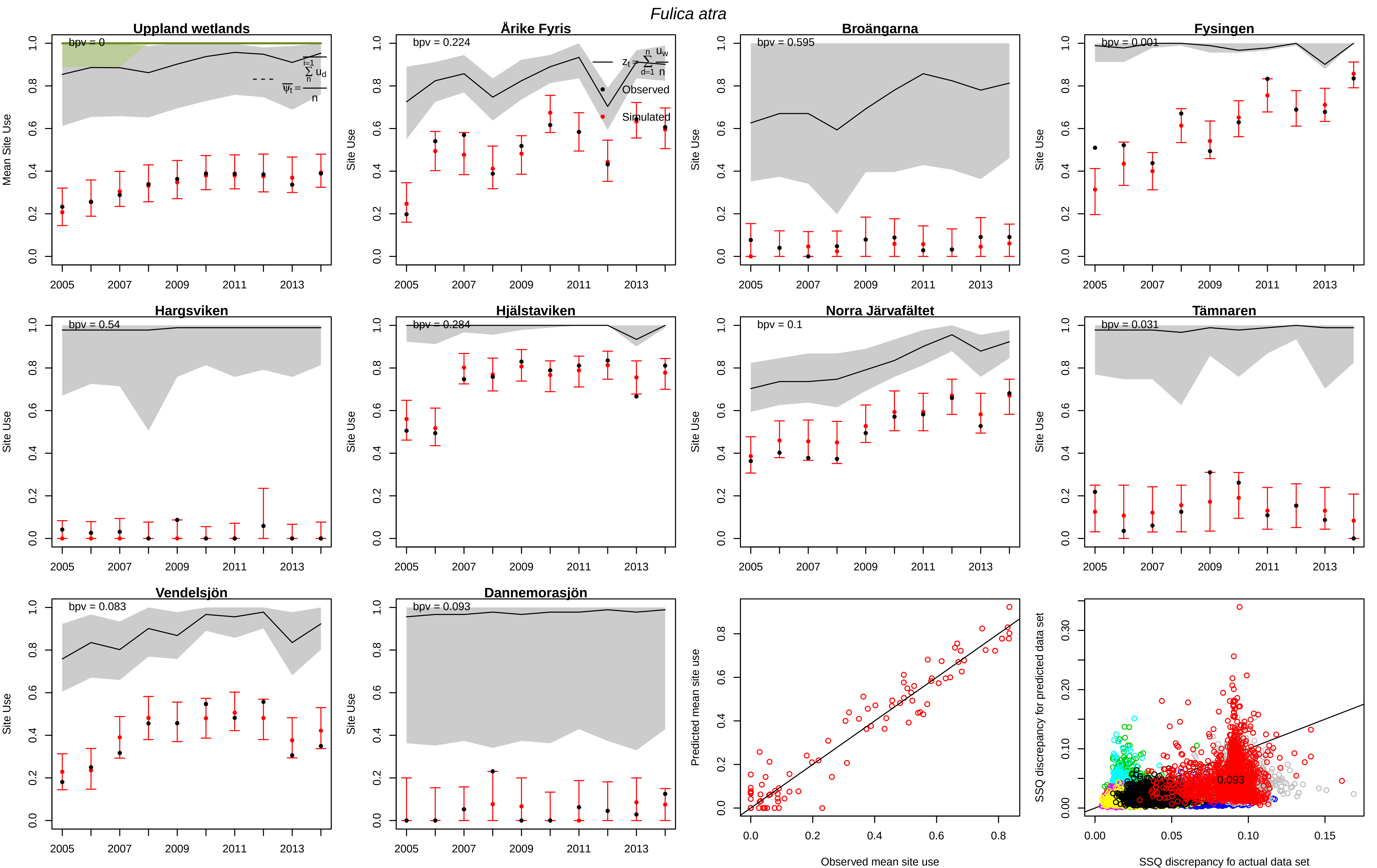

Fulica atra
Uppland wetlands
bpv = 0
Mean Site Use
Observed
Simulated
Årike Fyris
bpv = 0.224
Broängarna
bpv = 0.595
Fysingen
bpv = 0.001
Hargsviken
bpv = 0.54
Hjälstaviken
bpv = 0.284
Norra Järvafältet
bpv = 0.1
Tämnaren
bpv = 0.031
Vendelsjön
bpv = 0.083
Dannemorasjön
bpv = 0.093
Site Use
2005 2007 2009 2011 2013
Predicted mean site use
Observed mean site use
SSQ discrepancy for predicted data set
SSQ discrepancy fo actual data set
0.093

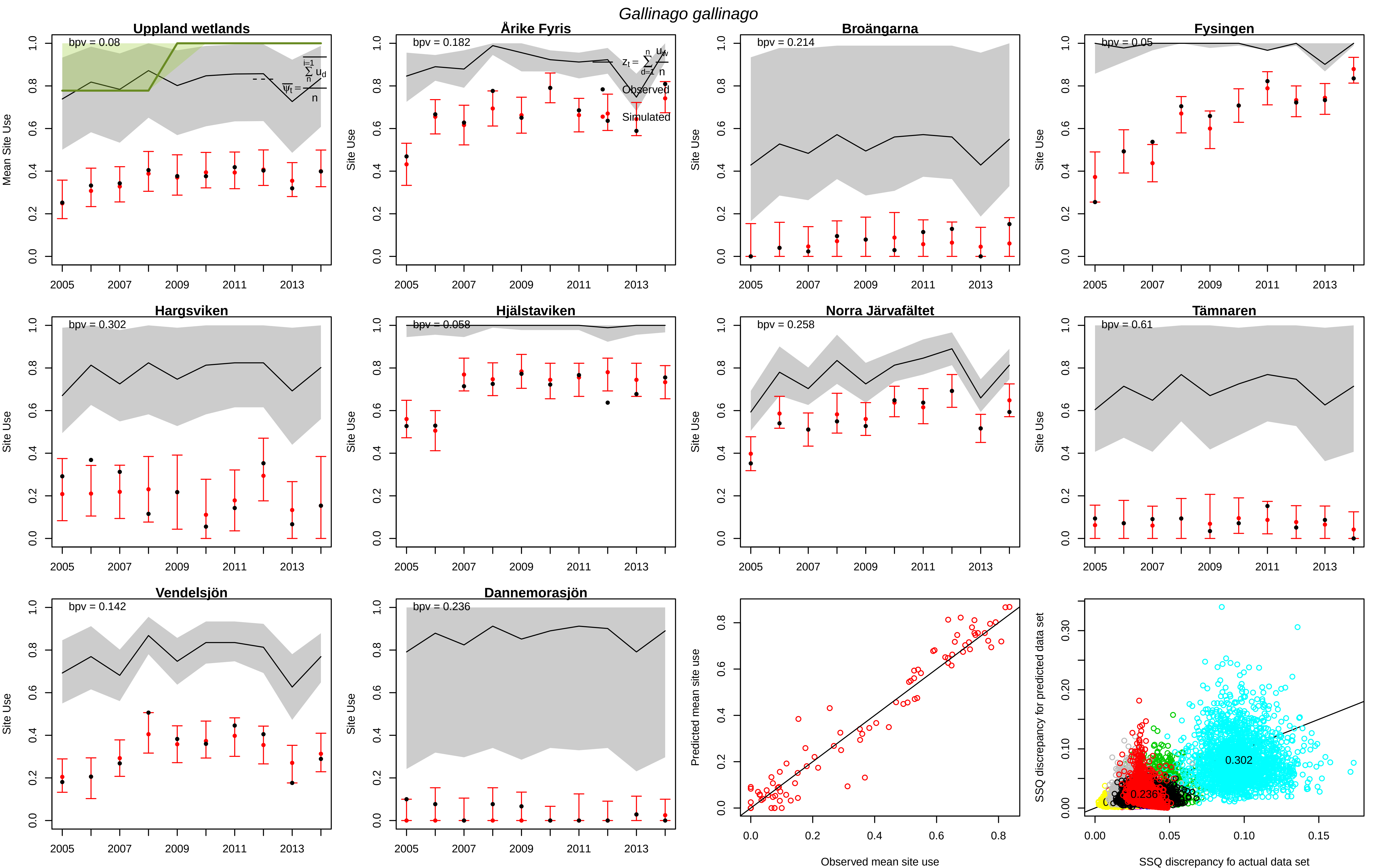

Gallinago gallinago
Uppland wetlands
bpv = 0.08
Mean Site Use
Årike Fyris
bpv = 0.182
Observed
Simulated
Broängarna
bpv = 0.214
Fysingen
bpv = 0.05
Hargsviken
bpv = 0.302
Hjälstaviken
bpv = 0.058
Norra Järvafältet
bpv = 0.258
Tämnaren
bpv = 0.61
Vendelsjön
bpv = 0.142
Dannemorasjön
bpv = 0.236
Site Use
Predicted mean site use
Observed mean site use
SSQ discrepancy for predicted data set
SSQ discrepancy fo actual data set
0.302
0.236

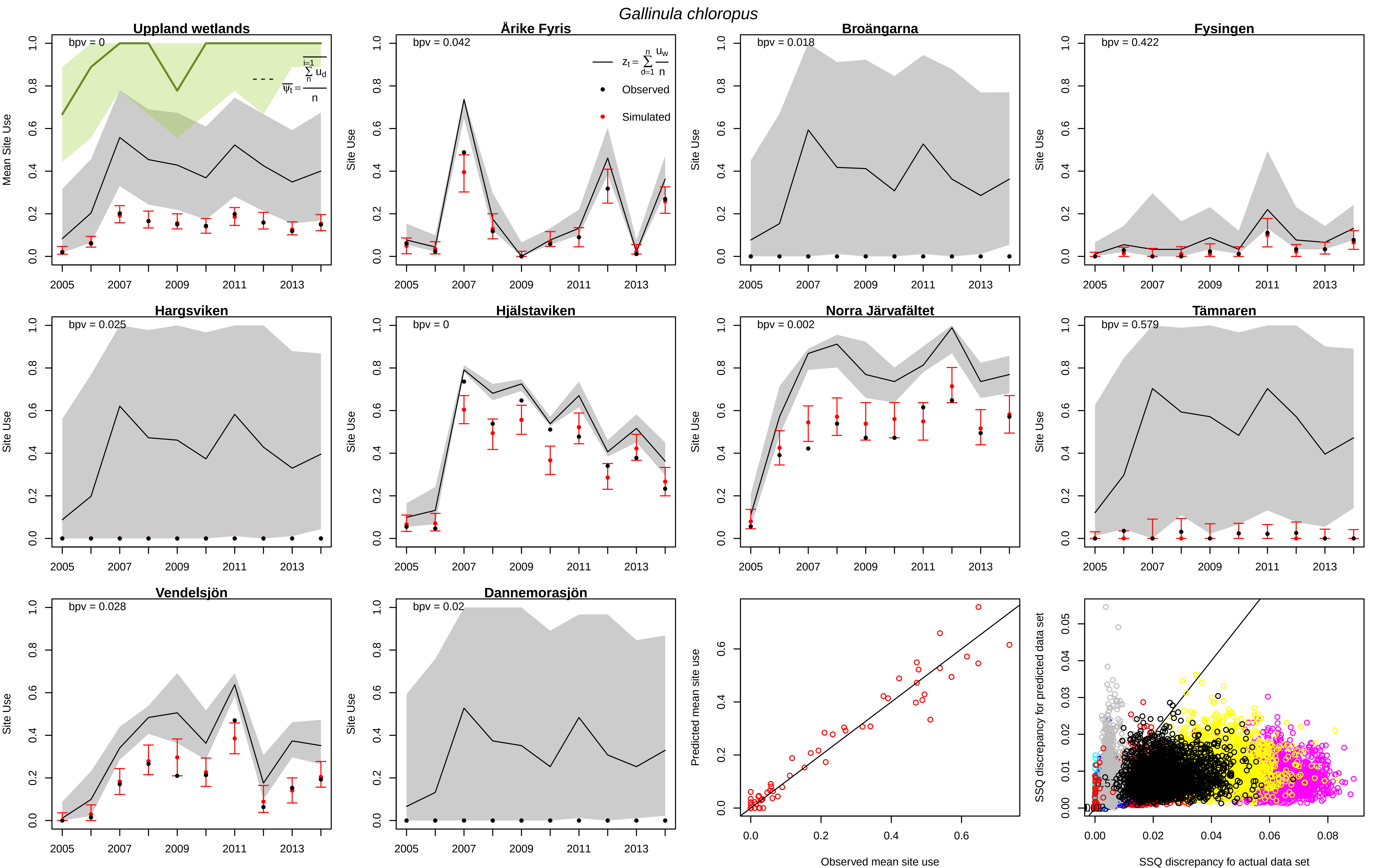
Gallinula chloropus
Uppland wetlands
bpv = 0
Mean Site Use
Årike Fyris
bpv = 0.042
Observed
Simulated
Site Use
Broängarna
bpv = 0.018
Fysingen
bpv = 0.422
Hargsviken
bpv = 0.025
Hjälstaviken
bpv = 0
Norra Järvafältet
bpv = 0.002
Tämnaren
bpv = 0.579
Vendelsjön
bpv = 0.028
Dannemorasjön
bpv = 0.02
Predicted mean site use
Observed mean site use
SSQ discrepancy for predicted data set
SSQ discrepancy fo actual data set

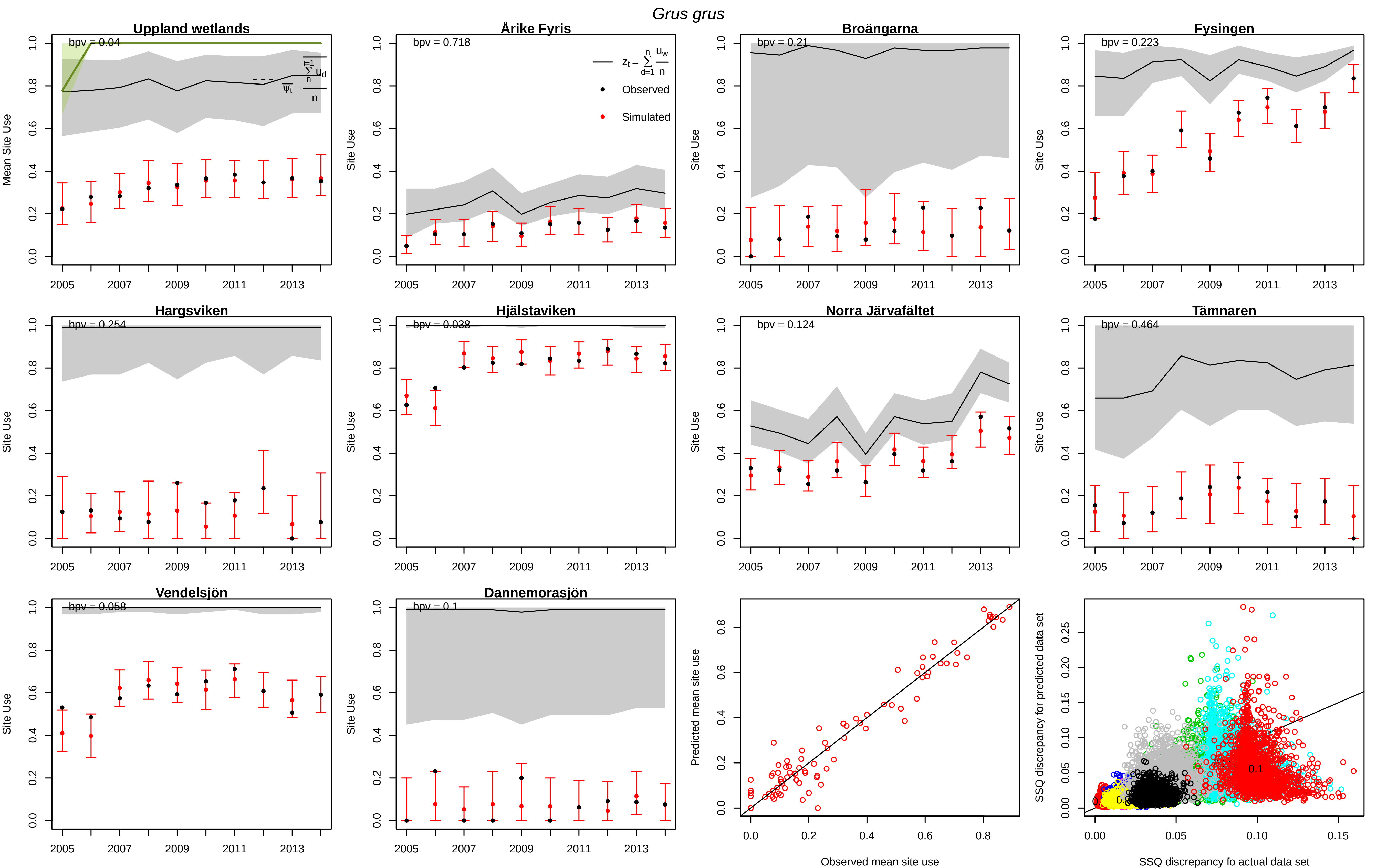

Grus grus
Uppland wetlands
bpv = 0.04
Mean Site Use
Årike Fyris
bpv = 0.718
Observed
Simulated
Broängarna
bpv = 0.21
Fysingen
bpv = 0.223
Hargsviken
bpv = 0.254
Hjälstaviken
bpv = 0.038
Norra Järvafältet
bpv = 0.124
Tämnaren
bpv = 0.464
Vendelsjön
bpv = 0.058
Dannemorasjön
bpv = 0.1
Site Use
Predicted mean site use
Observed mean site use
SSQ discrepancy for predicted data set
SSQ discrepancy fo actual data set

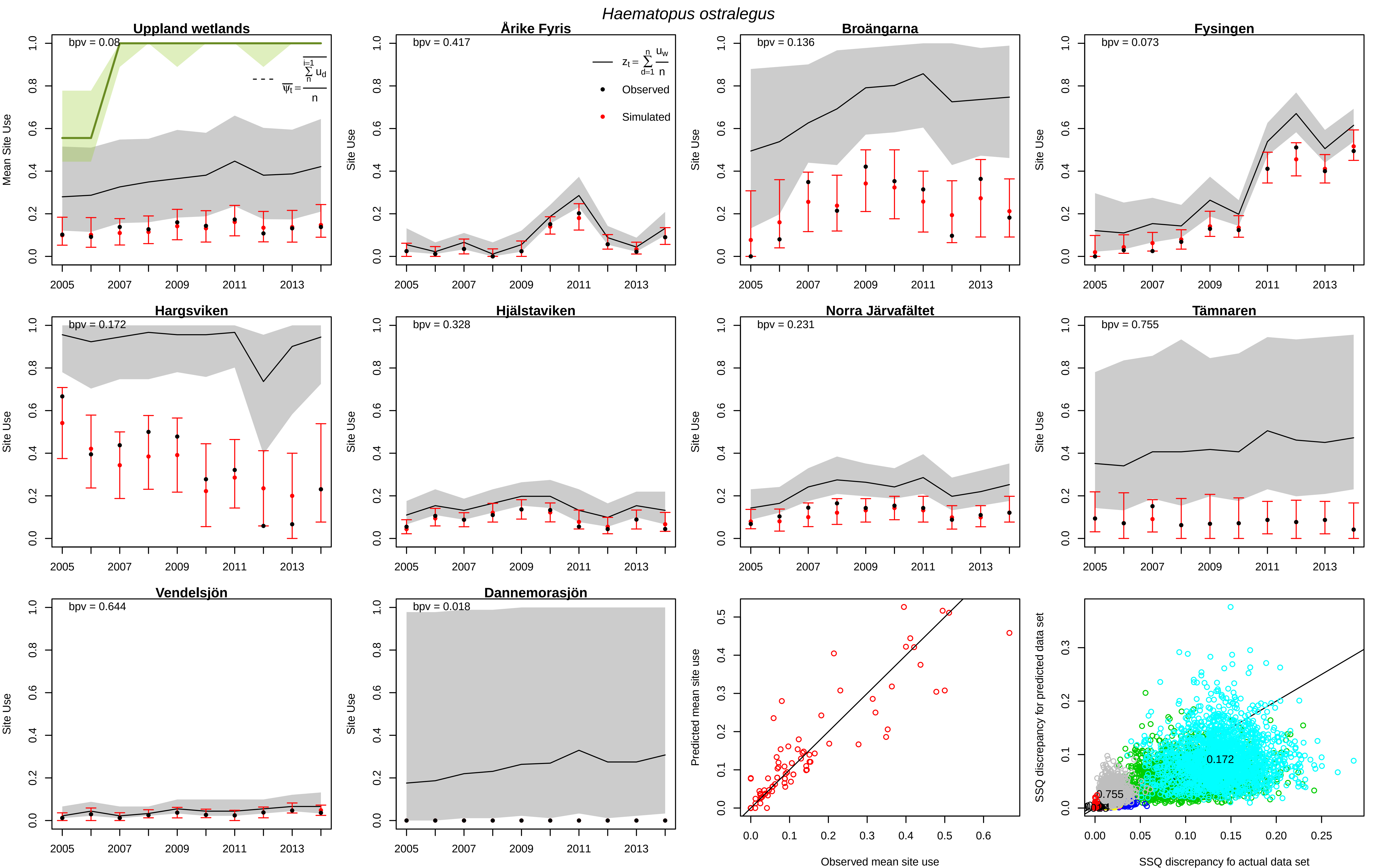

Haematopus ostralegus
Uppland wetlands
bpv = 0.08
Mean Site Use
Årike Fyris
bpv = 0.417
Observed
Simulated
Broängarna
bpv = 0.136
Fysingen
bpv = 0.073
Hargsviken
bpv = 0.172
Hjälstaviken
bpv = 0.328
Norra Järvafältet
bpv = 0.231
Tämnaren
bpv = 0.755
Vendelsjön
bpv = 0.644
Dannemorasjön
bpv = 0.018
Site Use
Predicted mean site use
Observed mean site use
SSQ discrepancy for predicted data set
SSQ discrepancy fo actual data set
0.172
0.755

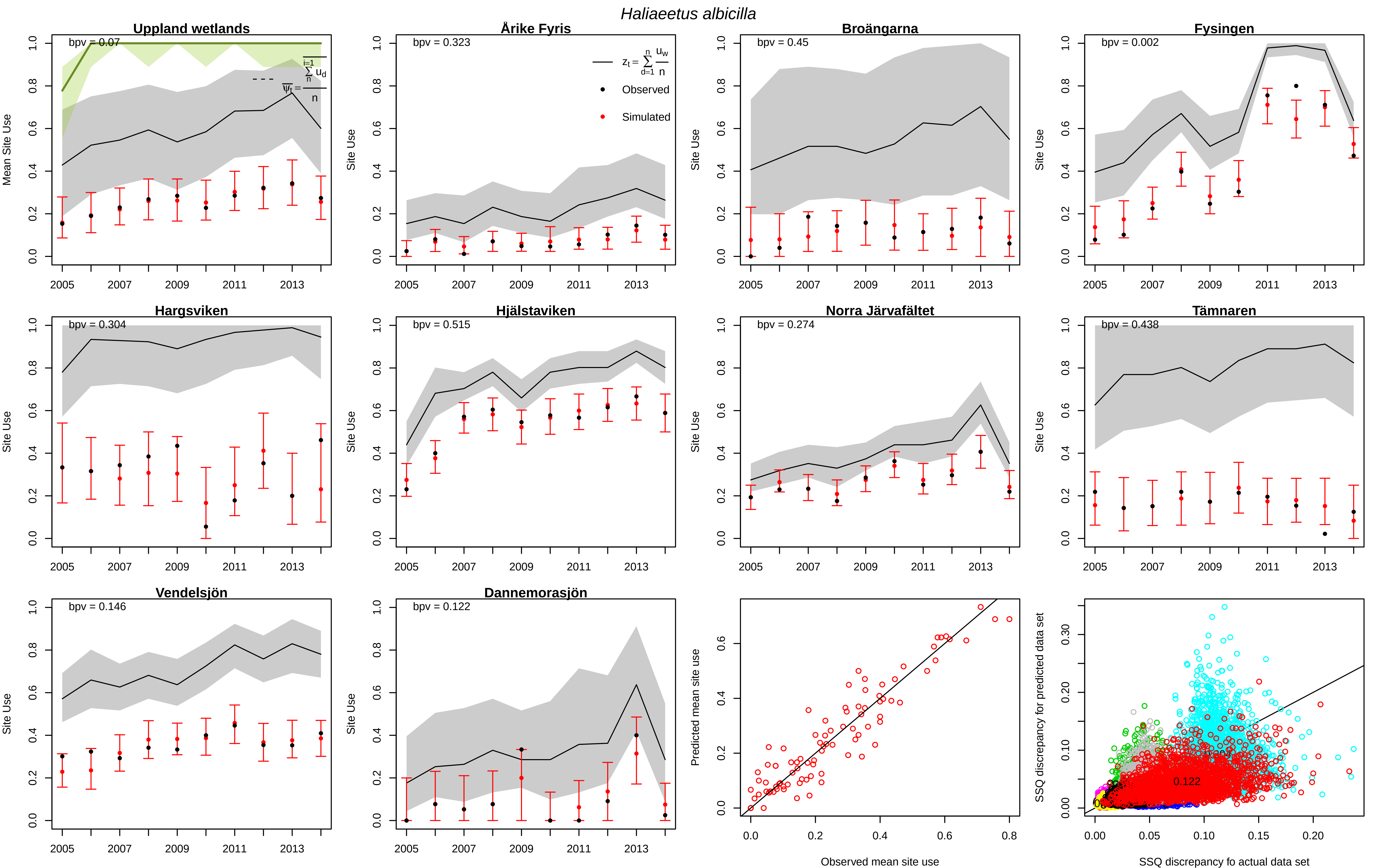

Haliaeetus albicilla
Uppland wetlands
bpv = 0.07
Mean Site Use
Årike Fyris
bpv = 0.323
Observed
Simulated
Broängarna
bpv = 0.45
Fysingen
bpv = 0.002
Hargsviken
bpv = 0.304
Hjälstaviken
bpv = 0.515
Norra Järvafältet
bpv = 0.274
Tämnaren
bpv = 0.438
Vendelsjön
bpv = 0.146
Dannemorasjön
bpv = 0.122
Site Use
Predicted mean site use
Observed mean site use
SSQ discrepancy for predicted data set
SSQ discrepancy fo actual data set
0.122

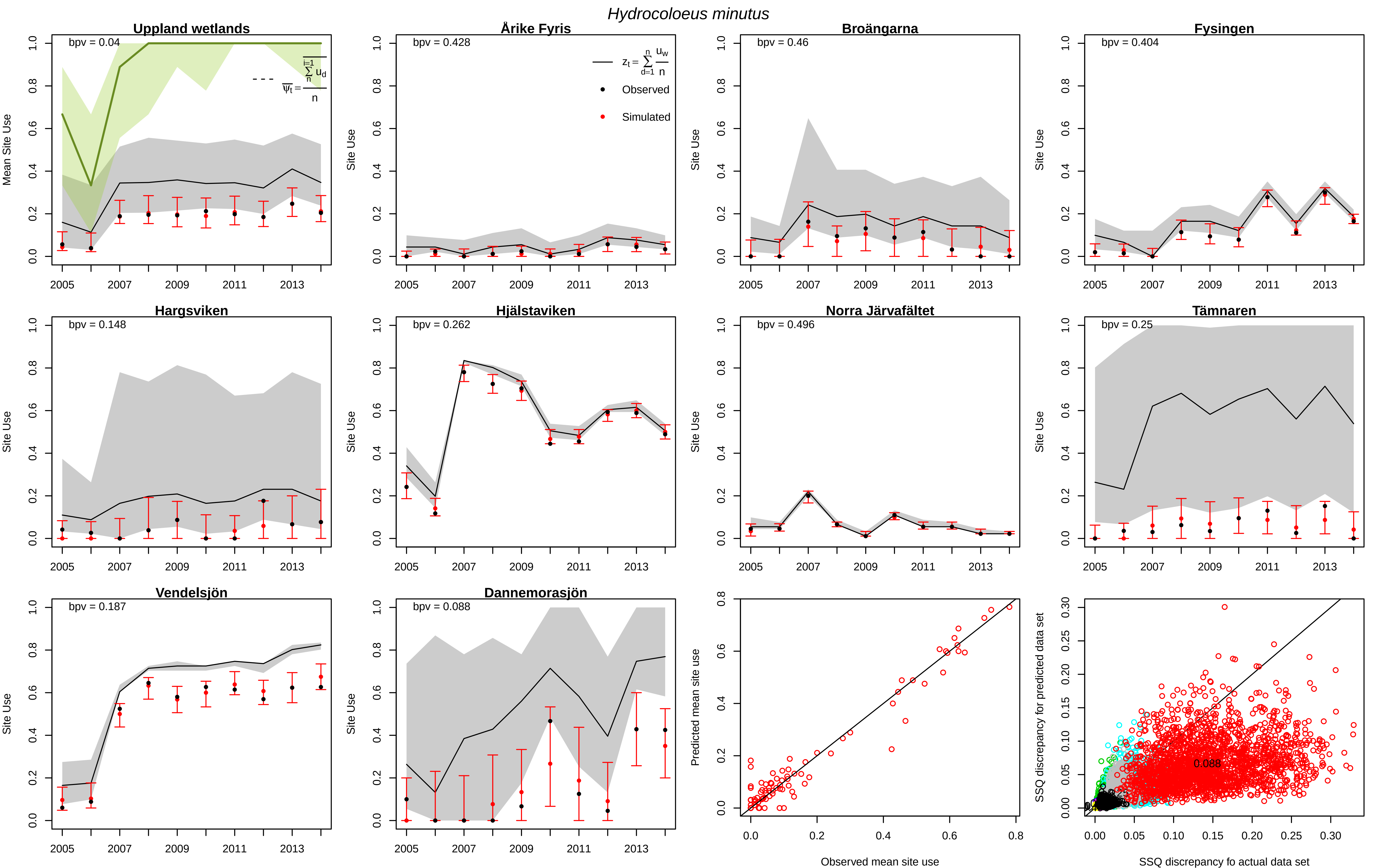
Hydrocoloeus minutus
Uppland wetlands
bpv = 0.04
Mean Site Use
Årike Fyris
bpv = 0.428
Observed
Simulated
Broängarna
bpv = 0.46
Fysingen
bpv = 0.404
Hargsviken
bpv = 0.148
Hjälstaviken
bpv = 0.262
Norra Järvafältet
bpv = 0.496
Tämnaren
bpv = 0.25
Vendelsjön
bpv = 0.187
Dannemorasjön
bpv = 0.088
Site Use
Predicted mean site use
Observed mean site use
SSQ discrepancy for predicted data set
SSQ discrepancy fo actual data set
0.088

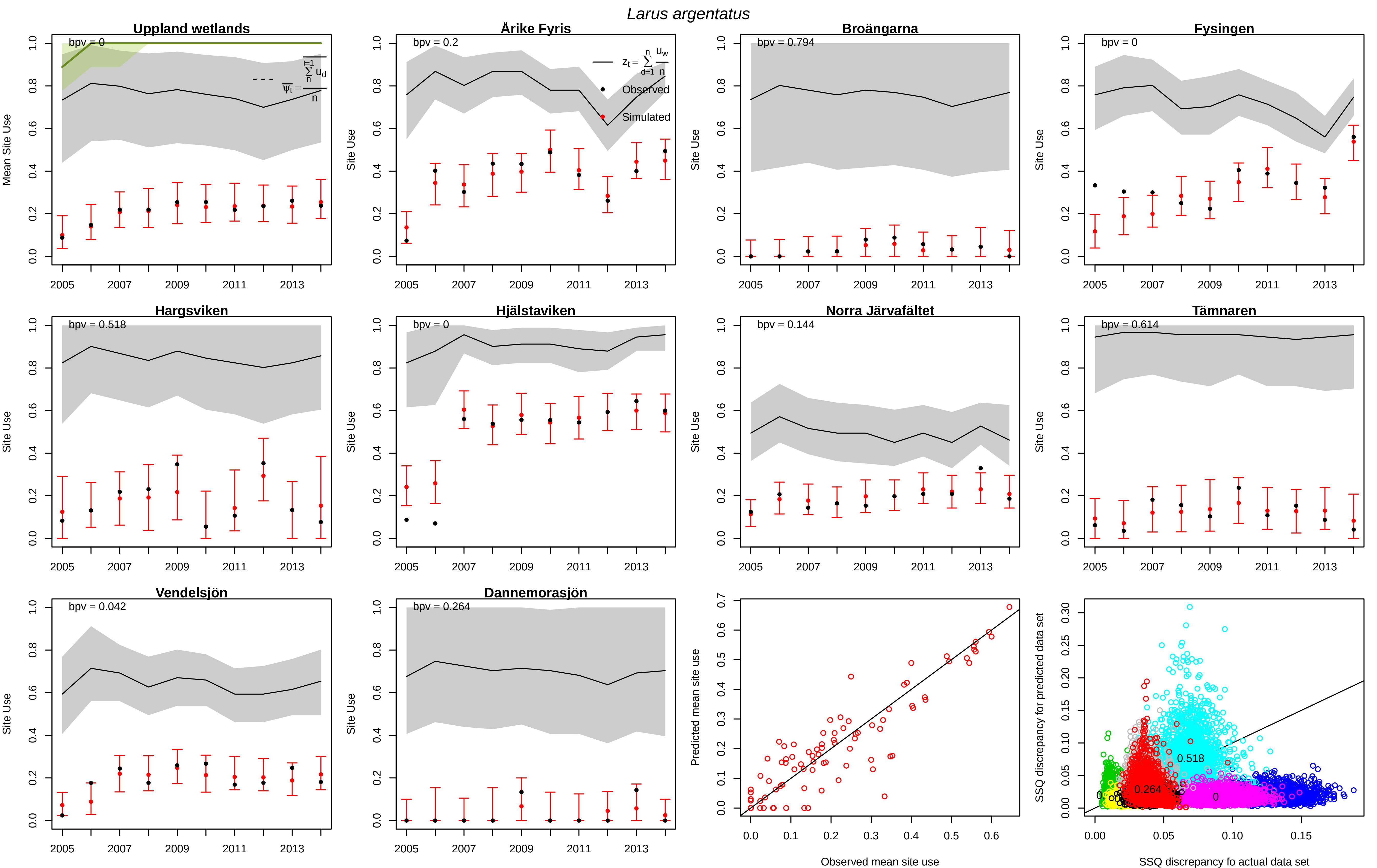
Larus argentatus
Uppland wetlands
bpv = 0
Årike Fyris
bpv = 0.2
Observed
Simulated
Broängarna
bpv = 0.794
Fysingen
bpv = 0
Hargsviken
bpv = 0.518
Hjälstaviken
bpv = 0
Norra Järvafältet
bpv = 0.144
Tämnaren
bpv = 0.614
Vendelsjön
bpv = 0.042
Dannemorasjön
bpv = 0.264
Mean Site Use
Site Use
Predicted mean site use
Observed mean site use
SSQ discrepancy for predicted data set
SSQ discrepancy fo actual data set
0.518
0.264
0

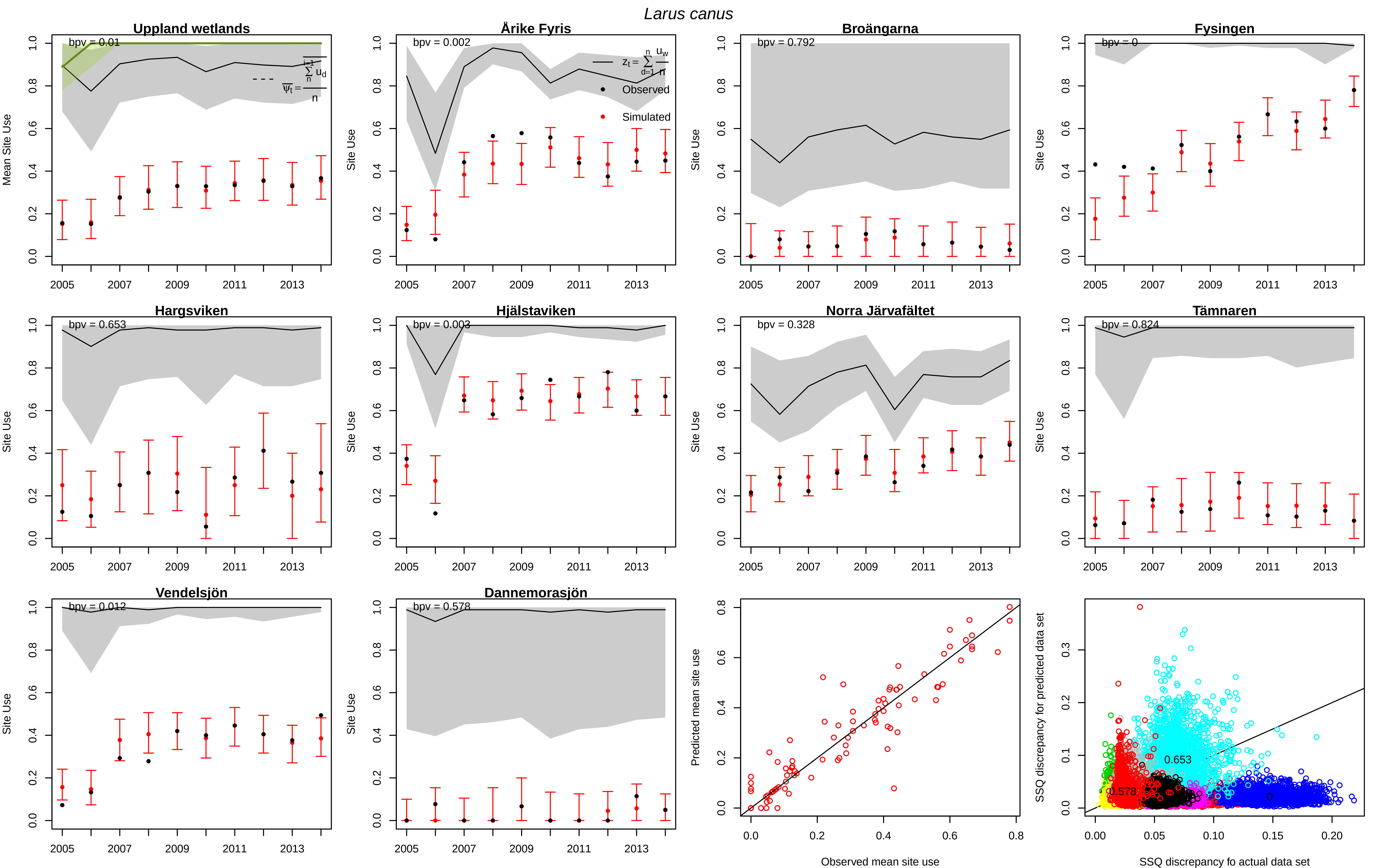

Larus canus
Uppland wetlands
bpv = 0.01
Mean Site Use
Årike Fyris
bpv = 0.002
Observed
Simulated
Site Use
Broängarna
bpv = 0.792
Fysingen
bpv = 0
Hargsviken
bpv = 0.653
Hjälstaviken
bpv = 0.003
Norra Järvafältet
bpv = 0.328
Tämnaren
bpv = 0.824
Vendelsjön
bpv = 0.012
Dannemorasjön
bpv = 0.578
Predicted mean site use
Observed mean site use
SSQ discrepancy for predicted data set
SSQ discrepancy fo actual data set
0.653
0.578
0

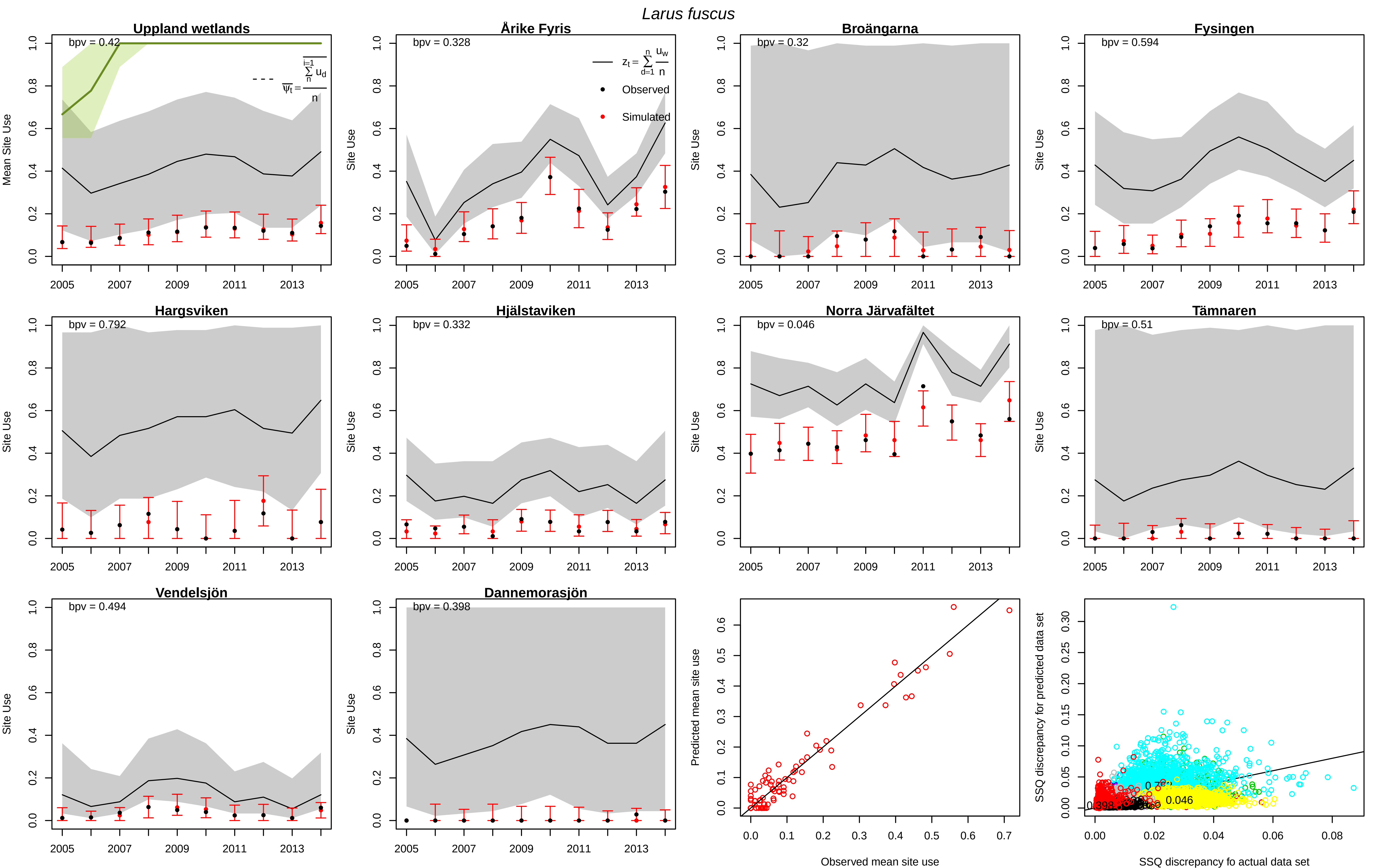

Larus fuscus
Uppland wetlands
bpv = 0.42
Mean Site Use
Årike Fyris
bpv = 0.328
Observed
Simulated
Site Use
Broängarna
bpv = 0.32
Fysingen
bpv = 0.594
Hargsviken
bpv = 0.792
Hjälstaviken
bpv = 0.332
Norra Järvafältet
bpv = 0.046
Tämnaren
bpv = 0.51
Vendelsjön
bpv = 0.494
Dannemorasjön
bpv = 0.398
2005
2007
2009
2011
2013
Predicted mean site use
Observed mean site use
SSQ discrepancy for predicted data set
SSQ discrepancy fo actual data set
0.046

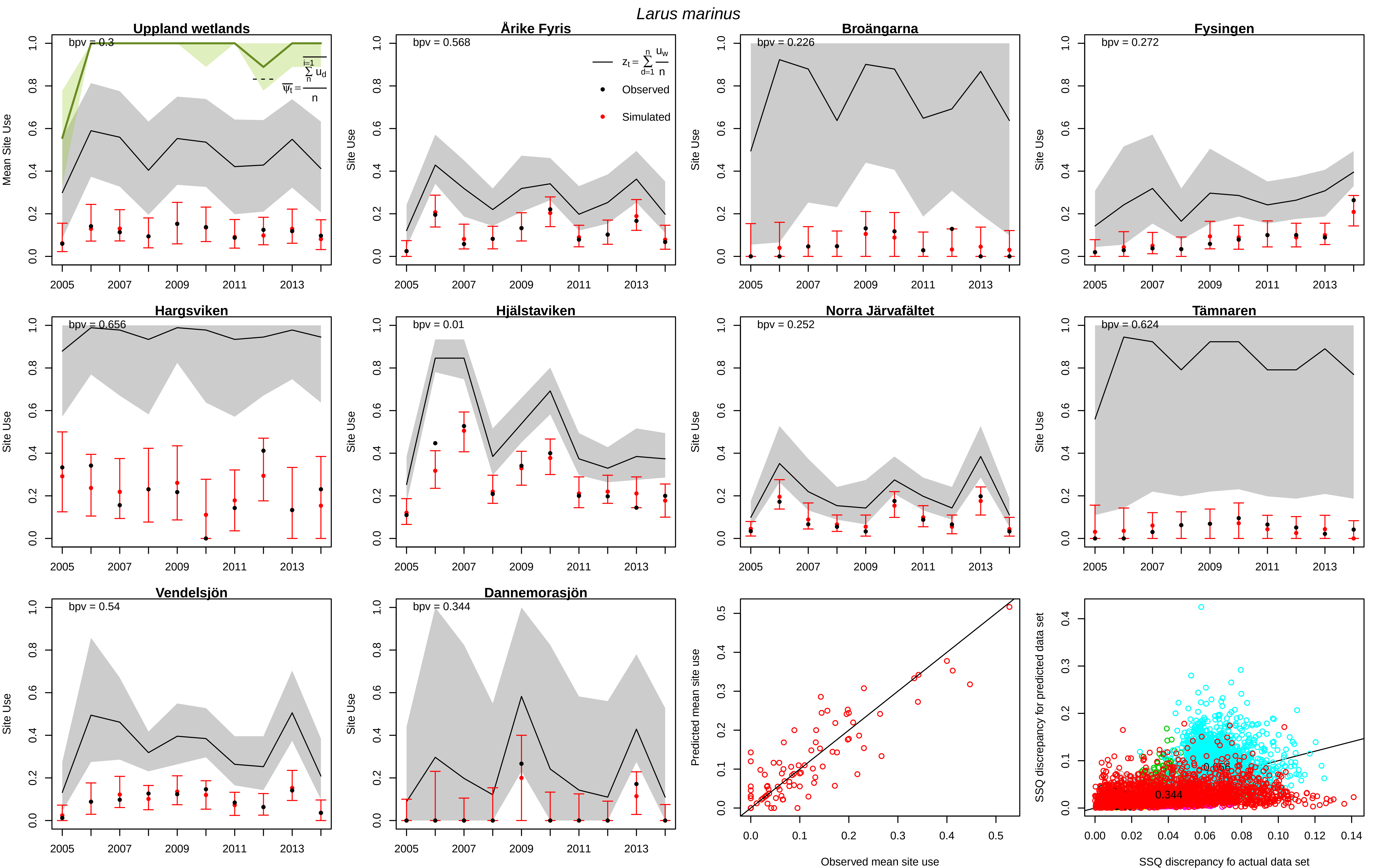

Larus marinus
Uppland wetlands
bpv = 0.3
Mean Site Use
Årike Fyris
bpv = 0.568
Observed
Simulated
Broängarna
bpv = 0.226
Fysingen
bpv = 0.272
Hargsviken
bpv = 0.656
Hjälstaviken
bpv = 0.01
Norra Järvafältet
bpv = 0.252
Tämnaren
bpv = 0.624
Vendelsjön
bpv = 0.54
Dannemorasjön
bpv = 0.344
Site Use
Predicted mean site use
Observed mean site use
SSQ discrepancy for predicted data set
SSQ discrepancy fo actual data set
0.344

Limosa limosa

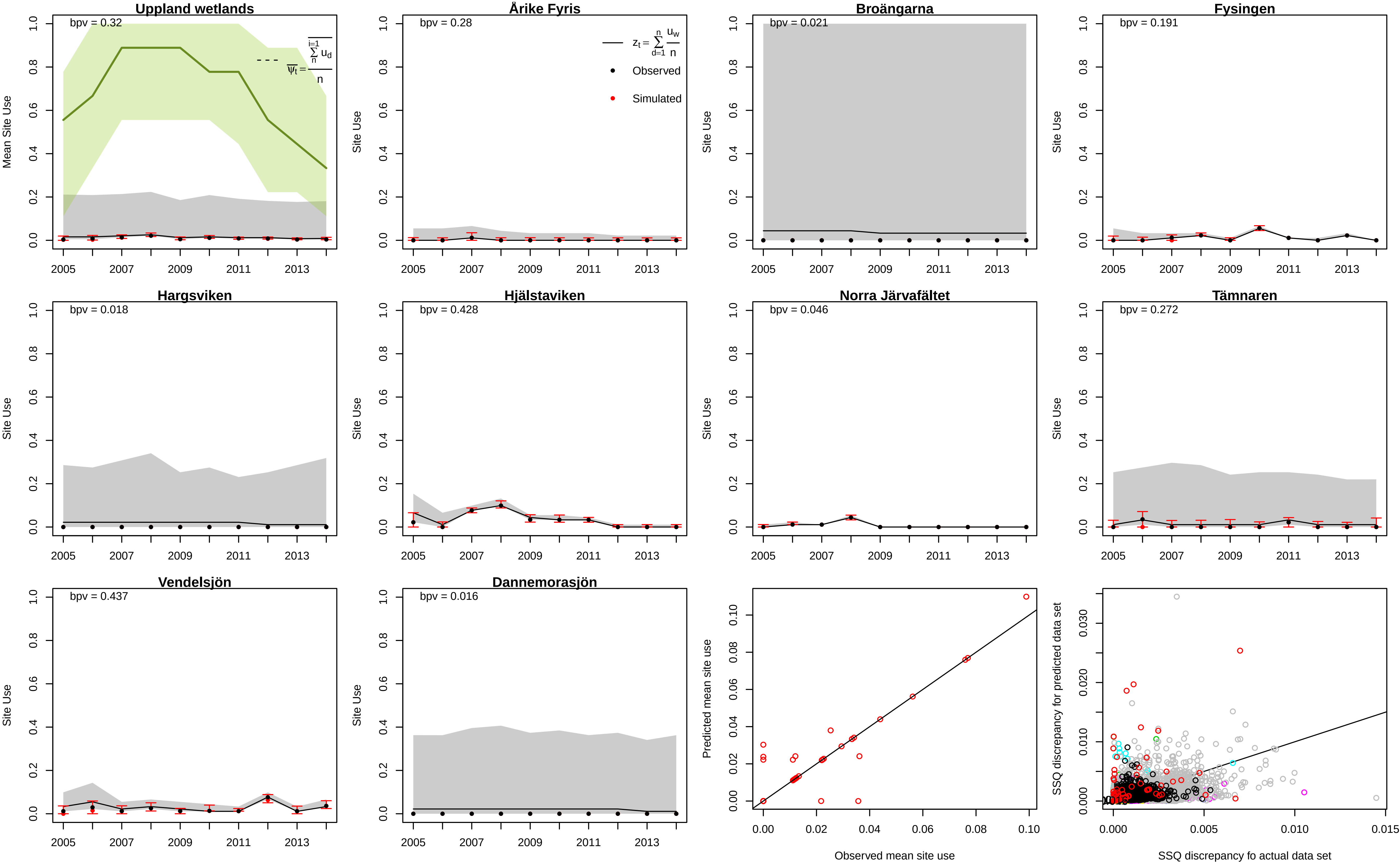

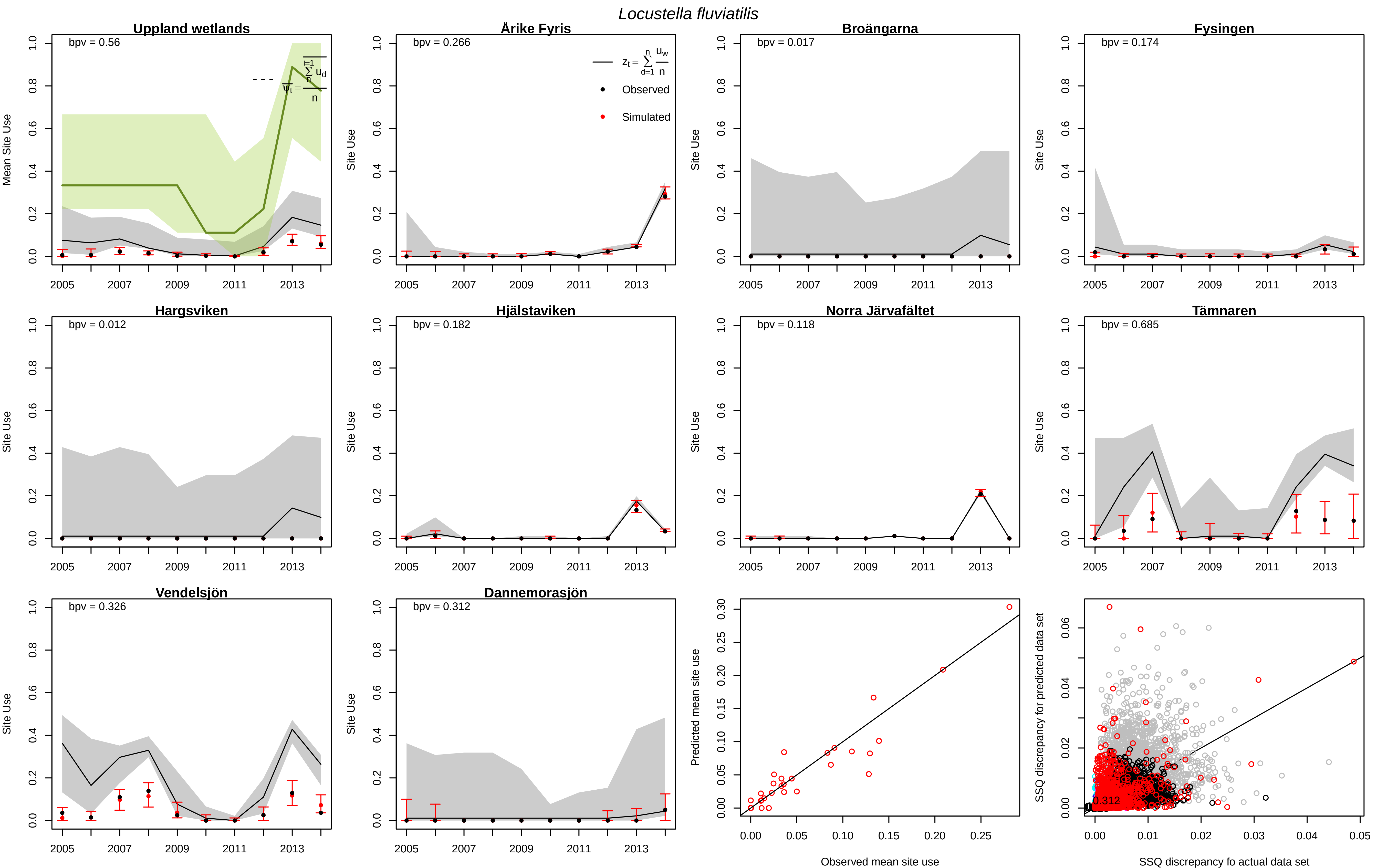
Locustella fluviatilis
Uppland wetlands
bpv = 0.56
Mean Site Use
Årike Fyris
bpv = 0.266
Observed
Simulated
Broängarna
bpv = 0.017
Fysingen
bpv = 0.174
Hargsviken
bpv = 0.012
Hjälstaviken
bpv = 0.182
Norra Järvafältet
bpv = 0.118
Tämnaren
bpv = 0.685
Vendelsjön
bpv = 0.326
Dannemorasjön
bpv = 0.312
Site Use
Predicted mean site use
Observed mean site use
SSQ discrepancy for predicted data set
SSQ discrepancy fo actual data set
0.312

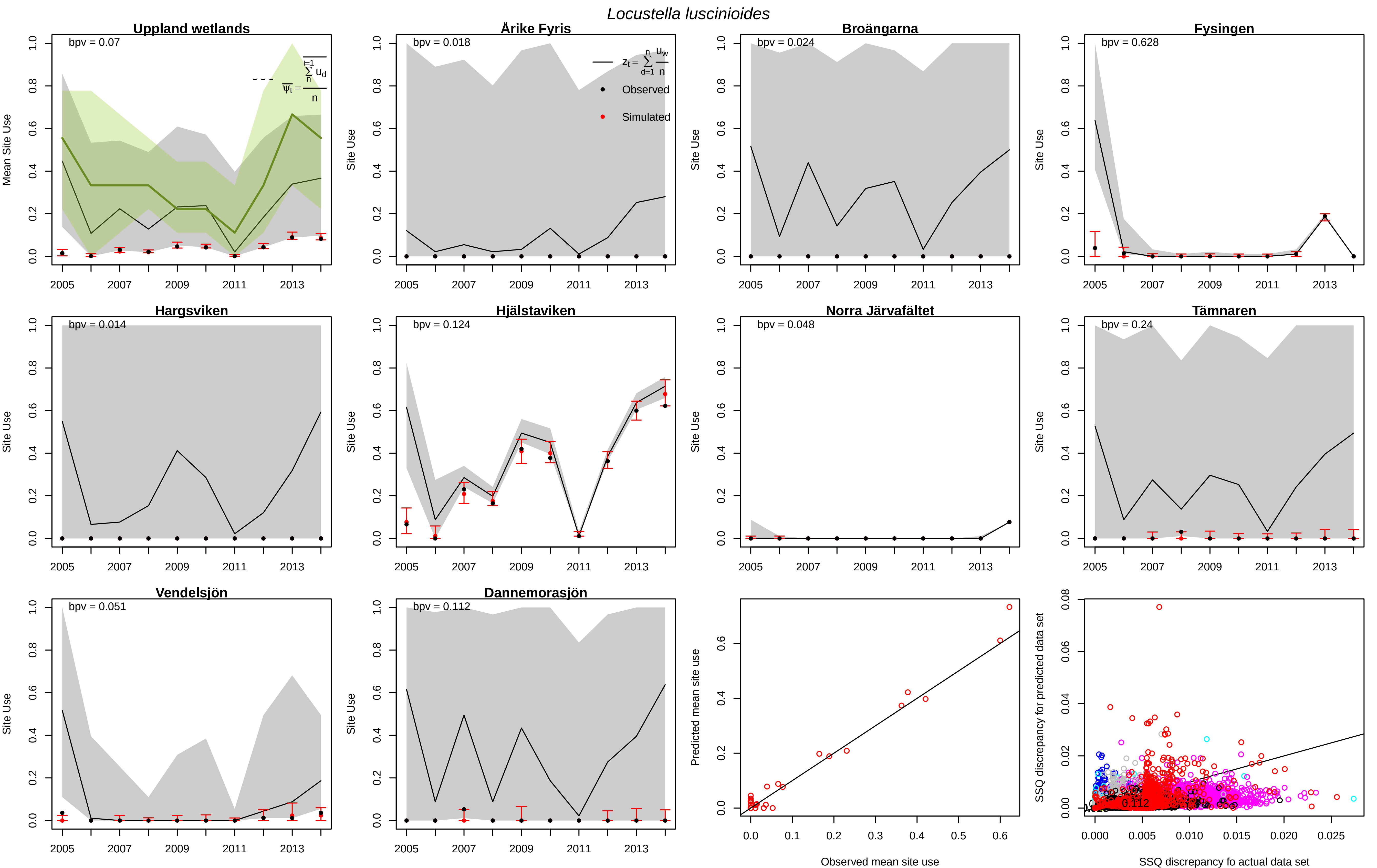

Locustella luscinioides
Uppland wetlands
bpv = 0.07
Mean Site Use
Årike Fyris
bpv = 0.018
Observed
Simulated
Site Use
Broängarna
bpv = 0.024
Fysingen
bpv = 0.628
Hargsviken
bpv = 0.014
Hjälstaviken
bpv = 0.124
Norra Järvafältet
bpv = 0.048
Tämnaren
bpv = 0.24
Vendelsjön
bpv = 0.051
Dannemorasjön
bpv = 0.112
Predicted mean site use
Observed mean site use
SSQ discrepancy for predicted data set
SSQ discrepancy fo actual data set
0.112

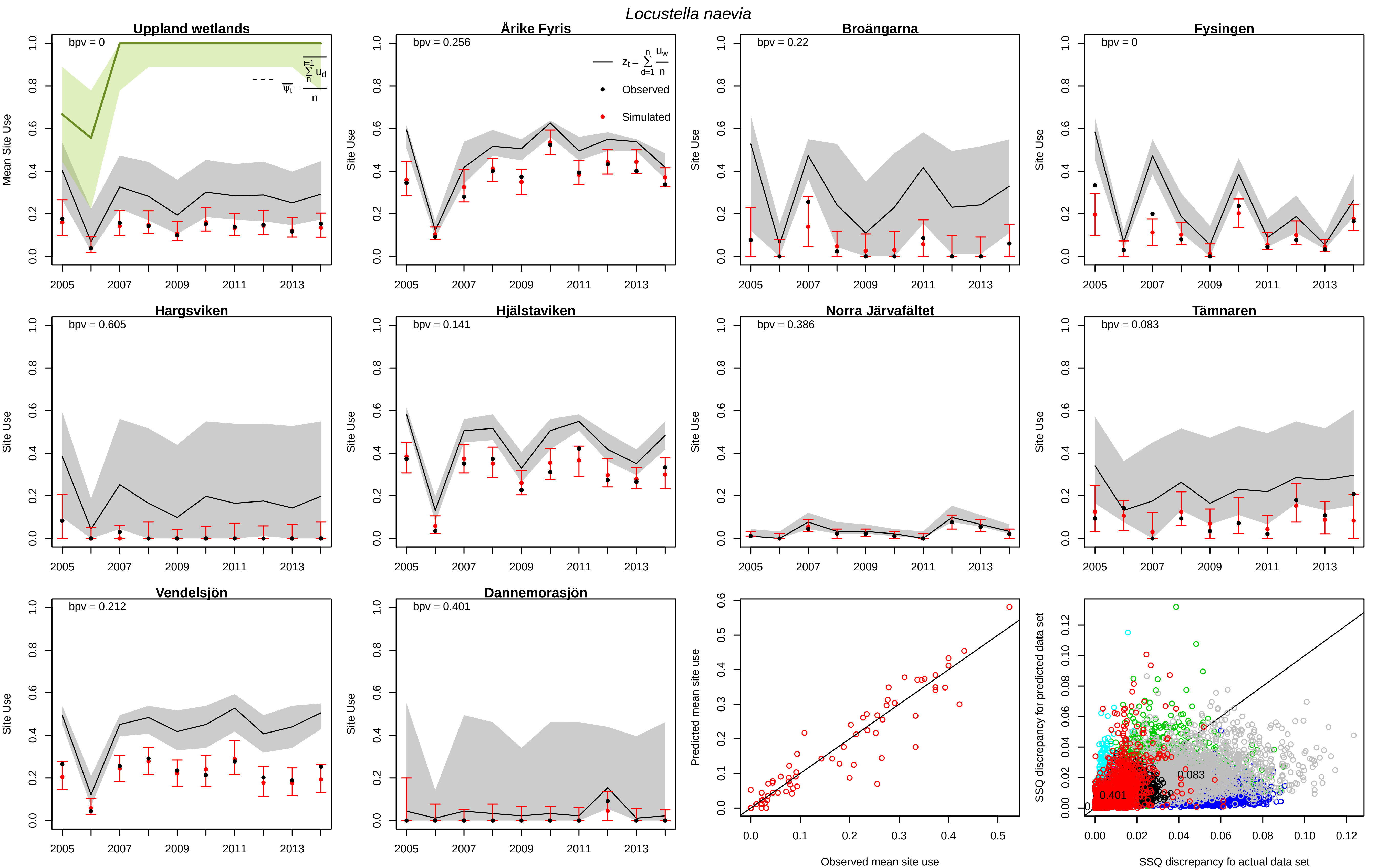

Locustella naevia
Uppland wetlands
bpv = 0
Mean Site Use
Årike Fyris
bpv = 0.256
Observed
Simulated
Broängarna
bpv = 0.22
Fysingen
bpv = 0
Hargsviken
bpv = 0.605
Hjälstaviken
bpv = 0.141
Norra Järvafältet
bpv = 0.386
Tämnaren
bpv = 0.083
Vendelsjön
bpv = 0.212
Dannemorasjön
bpv = 0.401
Site Use
Predicted mean site use
Observed mean site use
SSQ discrepancy for predicted data set
SSQ discrepancy fo actual data set
0.083
0.401

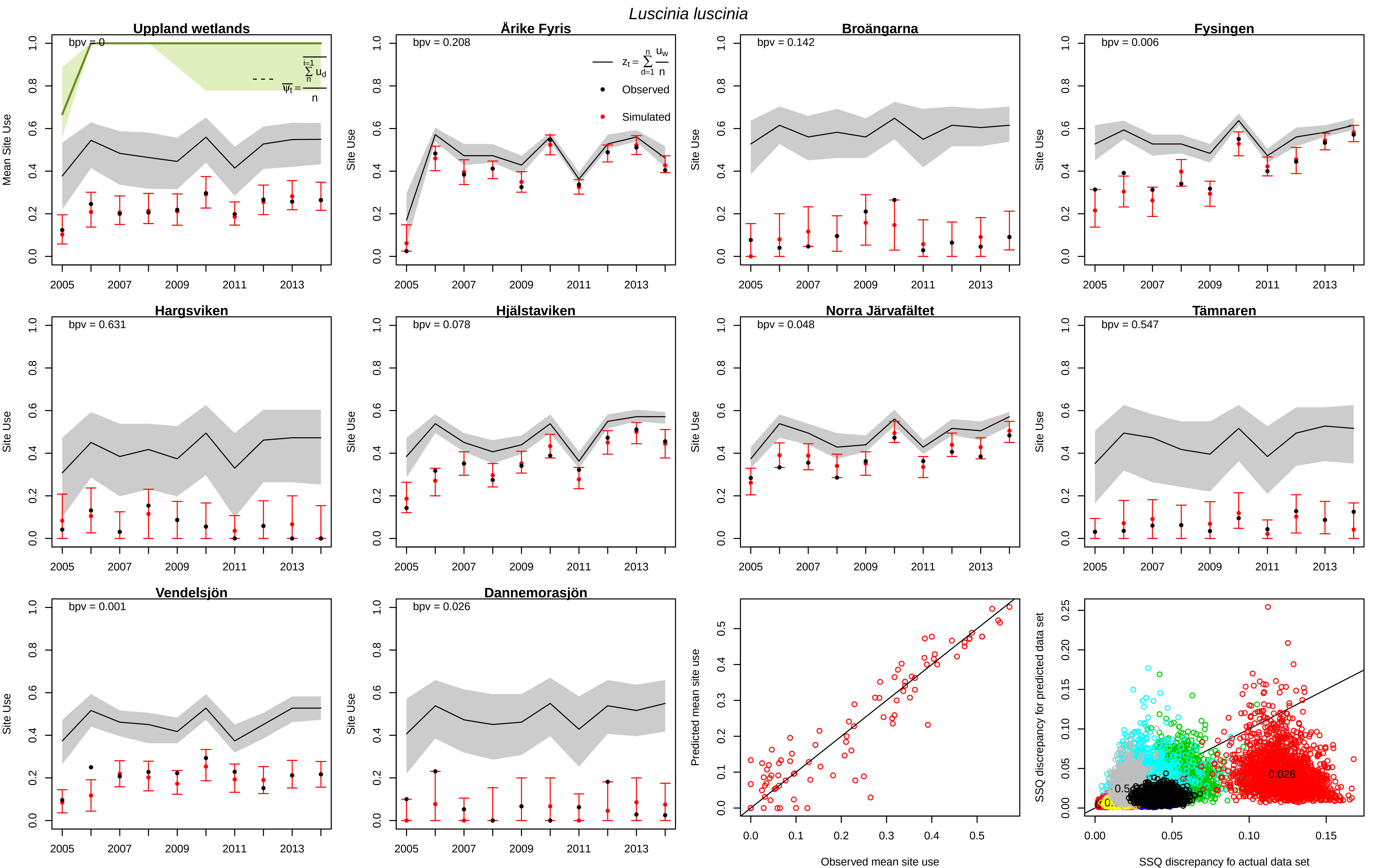

Luscinia luscinia
Uppland wetlands
bpv = 0
Mean Site Use
Årike Fyris
bpv = 0.208
Observed
Simulated
Broängarna
bpv = 0.142
Fysingen
bpv = 0.006
Hargsviken
bpv = 0.631
Hjälstaviken
bpv = 0.078
Norra Järvafältet
bpv = 0.048
Tämnaren
bpv = 0.547
Vendelsjön
bpv = 0.001
Dannemorasjön
bpv = 0.026
Site Use
Predicted mean site use
Observed mean site use
SSQ discrepancy for predicted data set
SSQ discrepancy fo actual data set
0.026

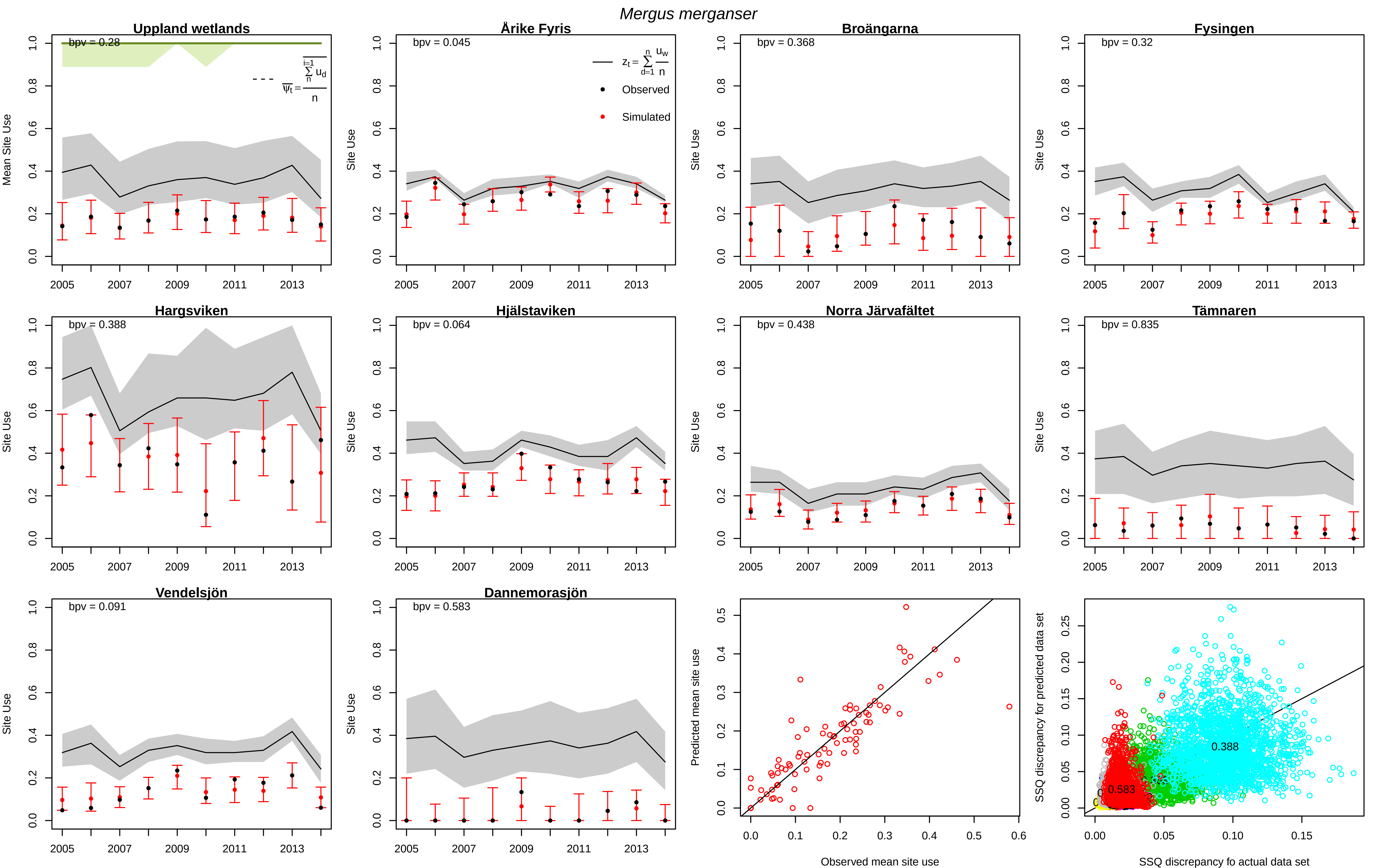
Mergus merganser
Uppland wetlands
bpv = 0.28
Mean Site Use
Årike Fyris
bpv = 0.045
Observed
Simulated
Broängarna
bpv = 0.368
Fysingen
bpv = 0.32
Hargsviken
bpv = 0.388
Hjälstaviken
bpv = 0.064
Norra Järvafältet
bpv = 0.438
Tämnaren
bpv = 0.835
Vendelsjön
bpv = 0.091
Dannemorasjön
bpv = 0.583
Site Use
2005
2007
2009
2011
2013
Predicted mean site use
Observed mean site use
SSQ discrepancy for predicted data set
SSQ discrepancy fo actual data set
0.388
0.583

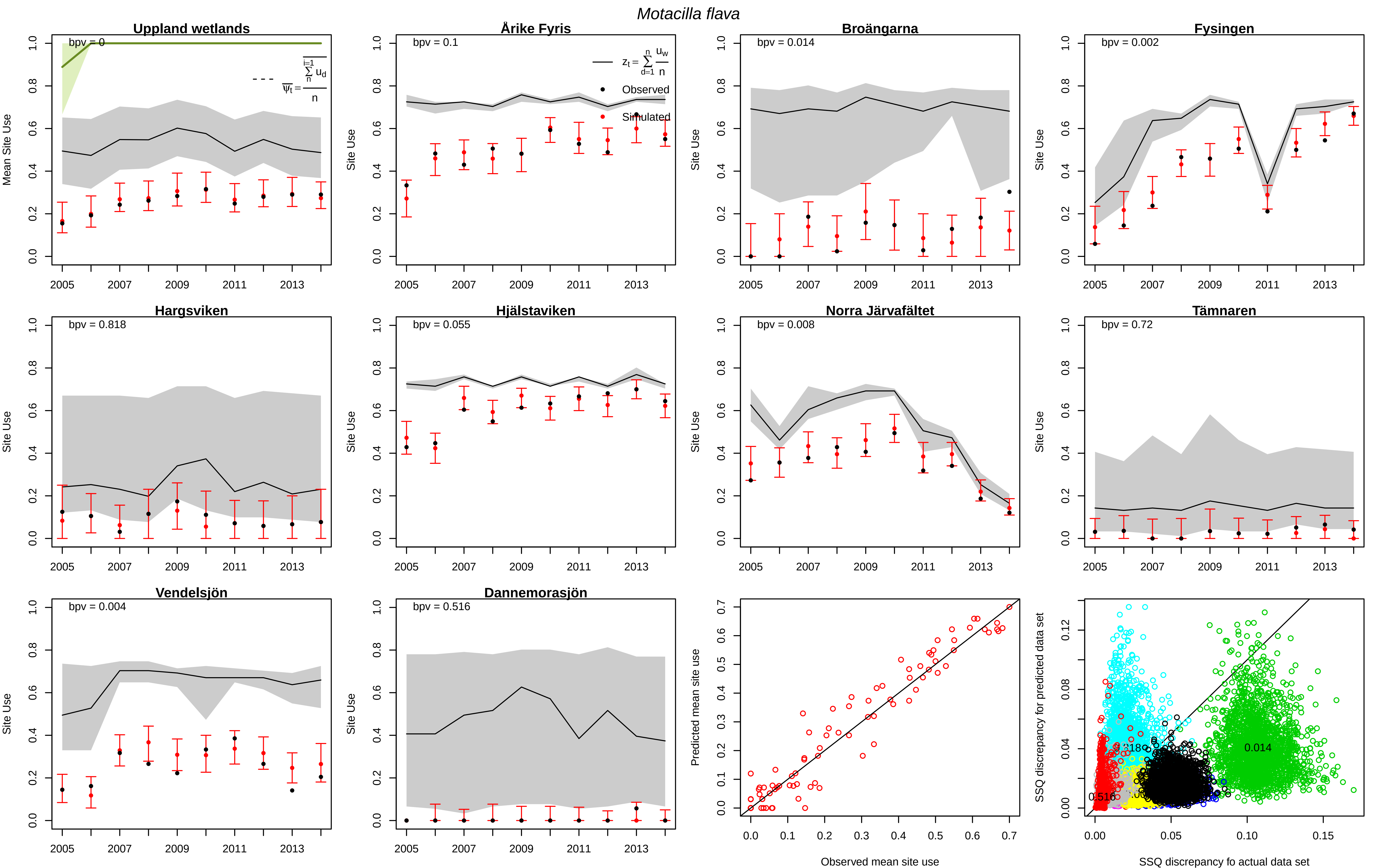

Motacilla flava
Uppland wetlands
bpv = 0
Mean Site Use
Årike Fyris
bpv = 0.1
Observed
Simulated
Broängarna
bpv = 0.014
Fysingen
bpv = 0.002
Hargsviken
bpv = 0.818
Hjälstaviken
bpv = 0.055
Norra Järvafältet
bpv = 0.008
Tämnaren
bpv = 0.72
Vendelsjön
bpv = 0.004
Dannemorasjön
bpv = 0.516
Site Use
Predicted mean site use
Observed mean site use
SSQ discrepancy for predicted data set
SSQ discrepancy fo actual data set
0.014
0.516

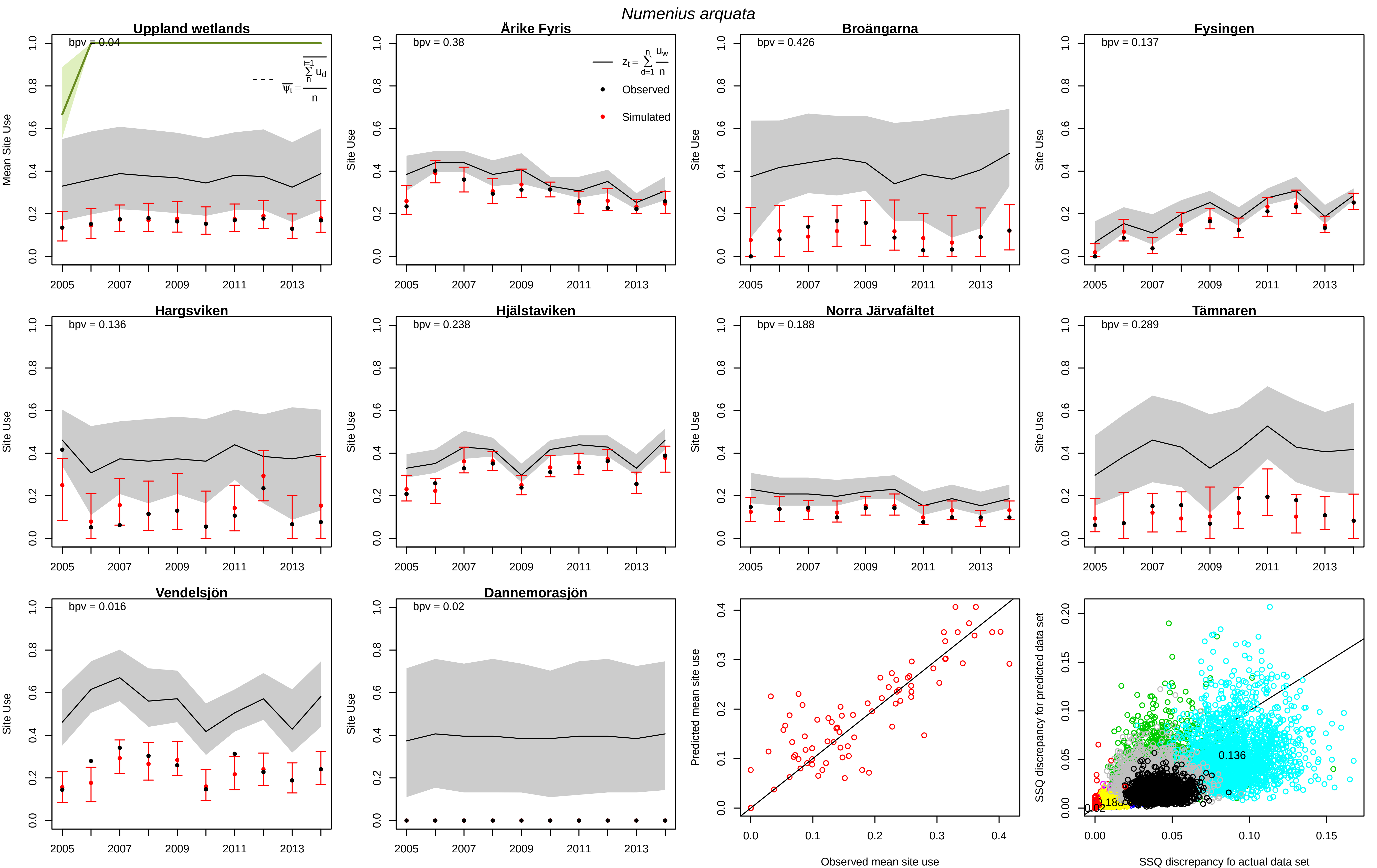

Numenius arquata
Uppland wetlands
bpv = 0.04
Mean Site Use
Årike Fyris
bpv = 0.38
Observed
Simulated
Broängarna
bpv = 0.426
Fysingen
bpv = 0.137
Hargsviken
bpv = 0.136
Hjälstaviken
bpv = 0.238
Norra Järvafältet
bpv = 0.188
Tämnaren
bpv = 0.289
Vendelsjön
bpv = 0.016
Dannemorasjön
bpv = 0.02
Site Use
Predicted mean site use
Observed mean site use
SSQ discrepancy for predicted data set
SSQ discrepancy fo actual data set
0.136

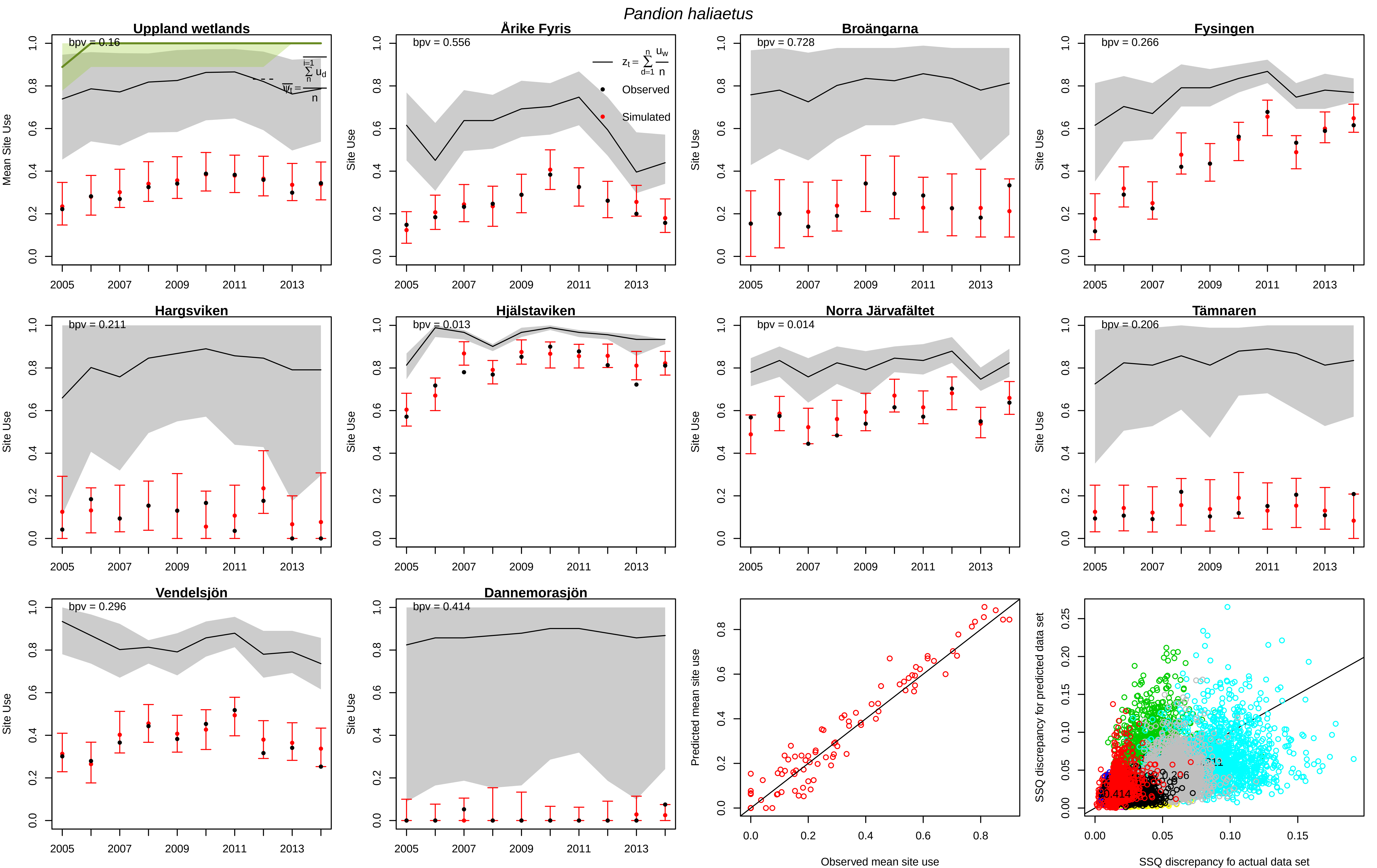
Pandion haliaetus
Uppland wetlands
bpv = 0.16
Mean Site Use
Årike Fyris
bpv = 0.556
Observed
Simulated
Broängarna
bpv = 0.728
Fysingen
bpv = 0.266
Hargsviken
bpv = 0.211
Hjälstaviken
bpv = 0.013
Norra Järvafältet
bpv = 0.014
Tämnaren
bpv = 0.206
Vendelsjön
bpv = 0.296
Dannemorasjön
bpv = 0.414
Site Use
Predicted mean site use
Observed mean site use
SSQ discrepancy for predicted data set
SSQ discrepancy fo actual data set

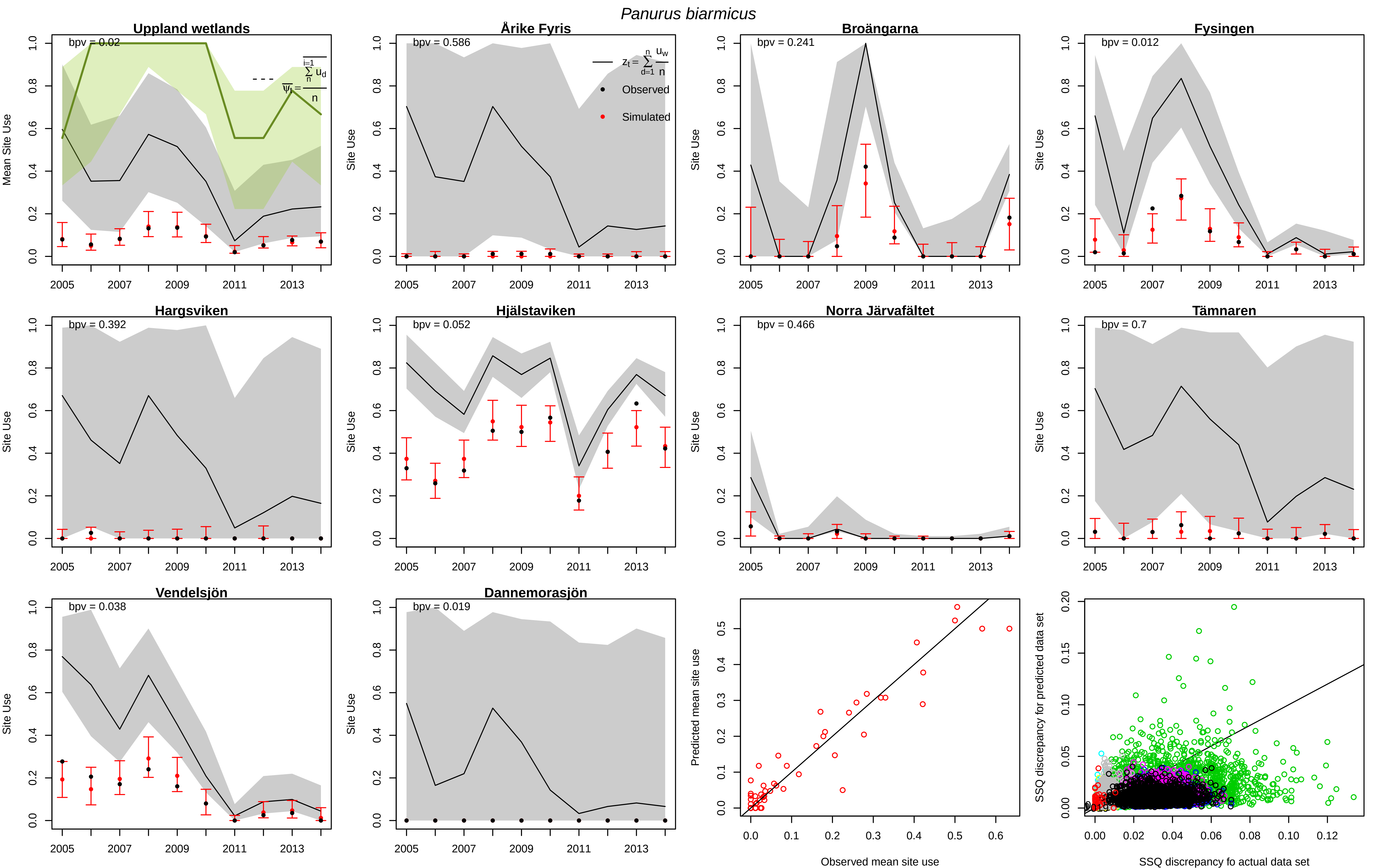

Panurus biarmicus
Uppland wetlands
Årike Fyris
Broängarna
Fysingen
Hargsviken
Hjälstaviken
Norra Järvafältet
Tämnaren
Vendelsjön
Dannemorasjön
bpv = 0.02
bpv = 0.586
bpv = 0.241
bpv = 0.012
bpv = 0.392
bpv = 0.052
bpv = 0.466
bpv = 0.7
bpv = 0.038
bpv = 0.019
Observed
Simulated
Mean Site Use
Site Use
Predicted mean site use
Observed mean site use
SSQ discrepancy for predicted data set
SSQ discrepancy fo actual data set

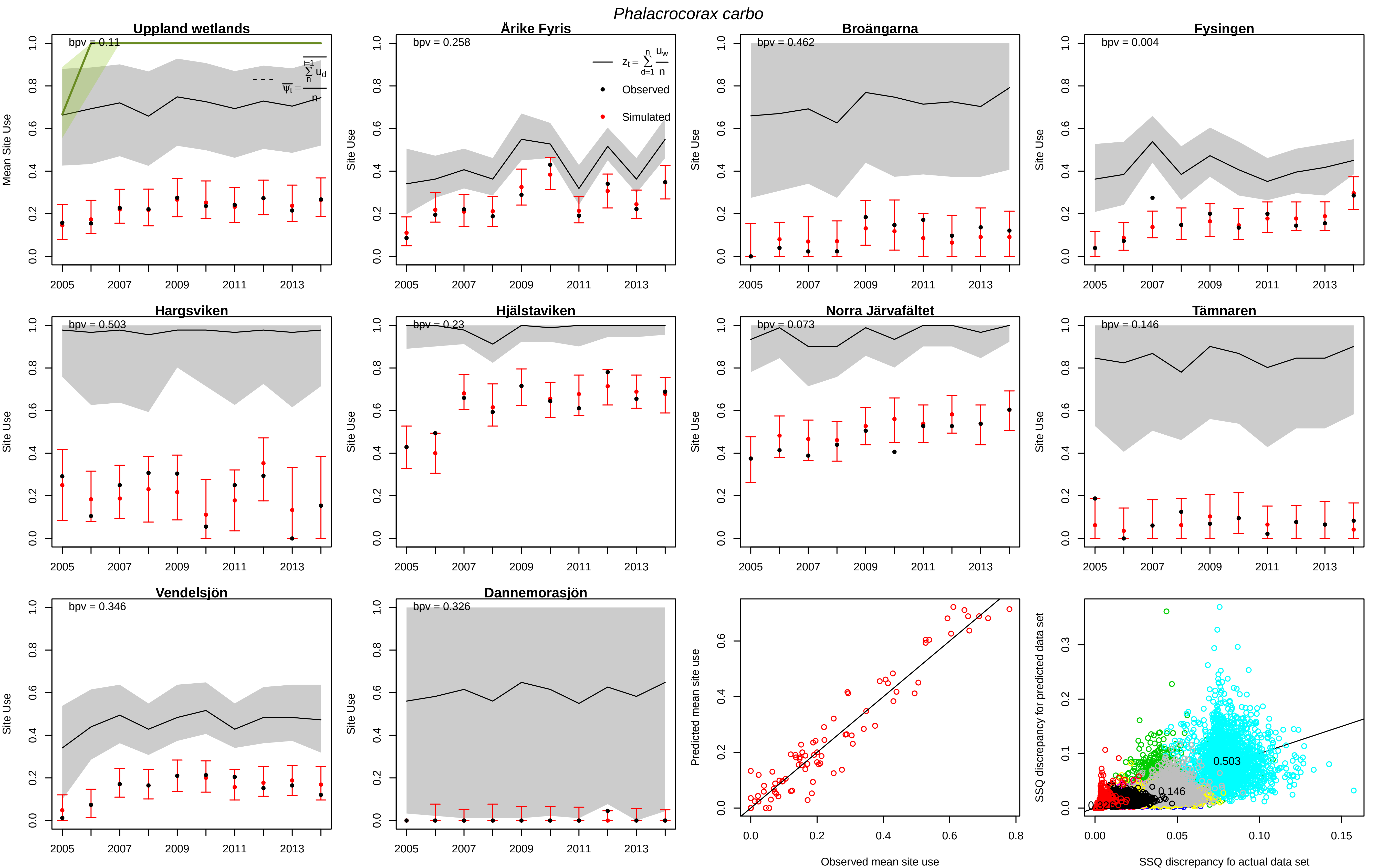

Phalacrocorax carbo
Uppland wetlands
bpv = 0.11
Mean Site Use
Årike Fyris
bpv = 0.258
Observed
Simulated
Broängarna
bpv = 0.462
Fysingen
bpv = 0.004
Hargsviken
bpv = 0.503
Hjälstaviken
bpv = 0.23
Norra Järvafältet
bpv = 0.073
Tämnaren
bpv = 0.146
Vendelsjön
bpv = 0.346
Dannemorasjön
bpv = 0.326
Site Use
Predicted mean site use
Observed mean site use
SSQ discrepancy for predicted data set
SSQ discrepancy fo actual data set

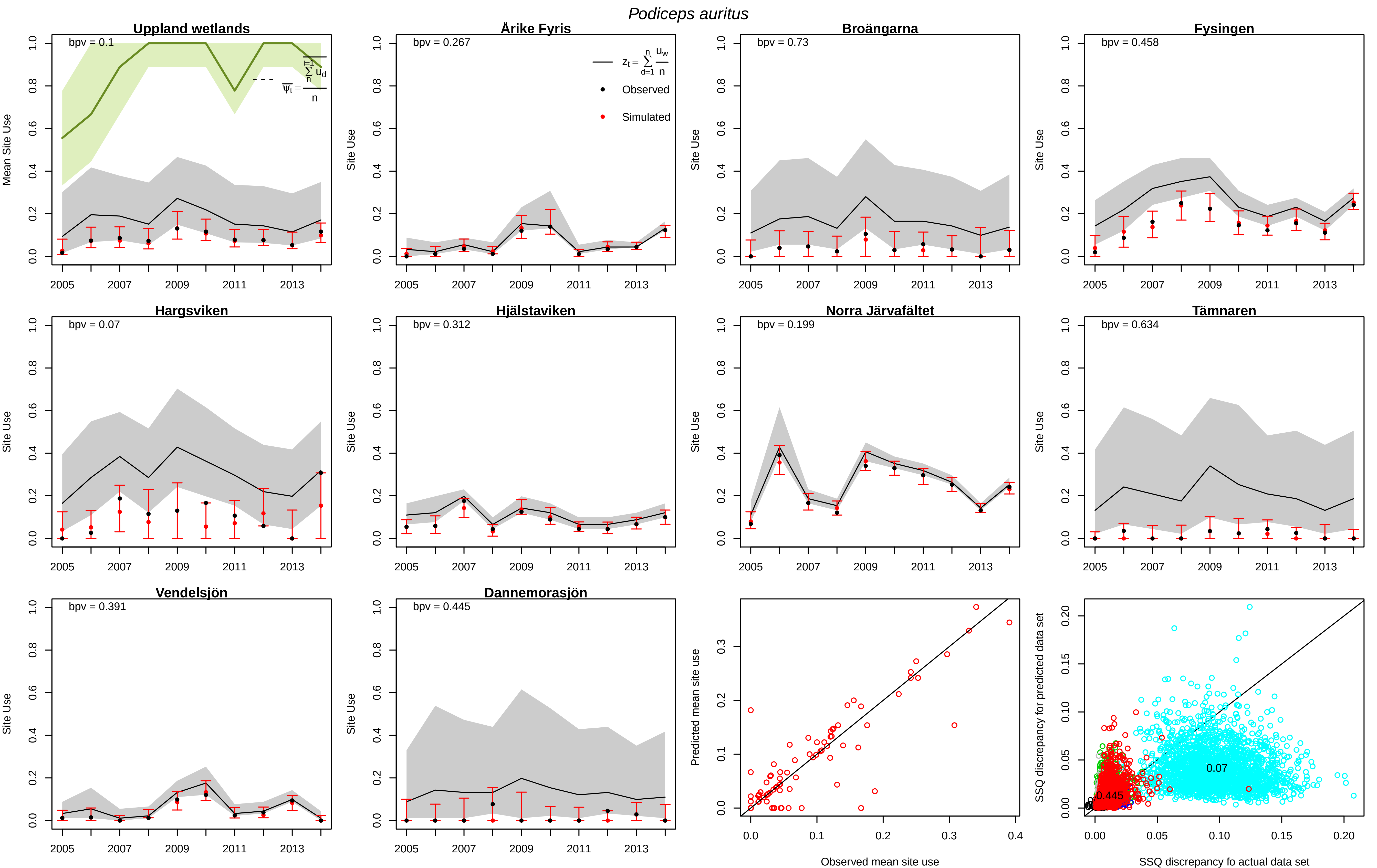
Podiceps auritus
Uppland wetlands
bpv = 0.1
Mean Site Use
Årike Fyris
bpv = 0.267
Observed
Simulated
Site Use
Broängarna
bpv = 0.73
Fysingen
bpv = 0.458
Hargsviken
bpv = 0.07
Hjälstaviken
bpv = 0.312
Norra Järvafältet
bpv = 0.199
Tämnaren
bpv = 0.634
Vendelsjön
bpv = 0.391
Dannemorasjön
bpv = 0.445
2005
2007
2009
2011
2013
Predicted mean site use
Observed mean site use
SSQ discrepancy for predicted data set
SSQ discrepancy fo actual data set
0.07
0.445

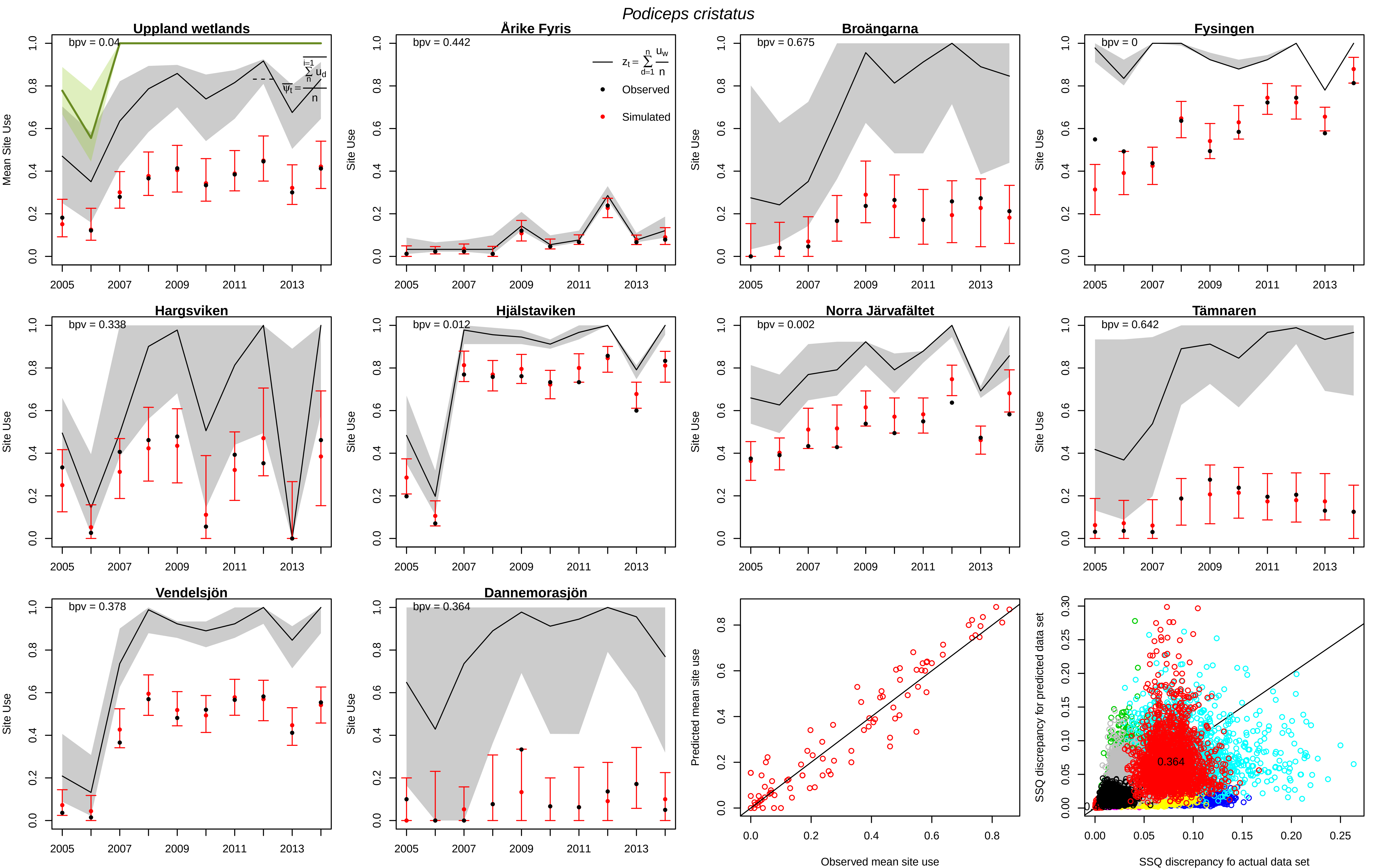

Podiceps cristatus
Uppland wetlands
bpv = 0.04
Mean Site Use
Årike Fyris
bpv = 0.442
Observed
Simulated
Broängarna
bpv = 0.675
Fysingen
bpv = 0
Hargsviken
bpv = 0.338
Hjälstaviken
bpv = 0.012
Norra Järvafältet
bpv = 0.002
Tämnaren
bpv = 0.642
Vendelsjön
bpv = 0.378
Dannemorasjön
bpv = 0.364
Site Use
Predicted mean site use
Observed mean site use
0.364
SSQ discrepancy for predicted data set
SSQ discrepancy fo actual data set

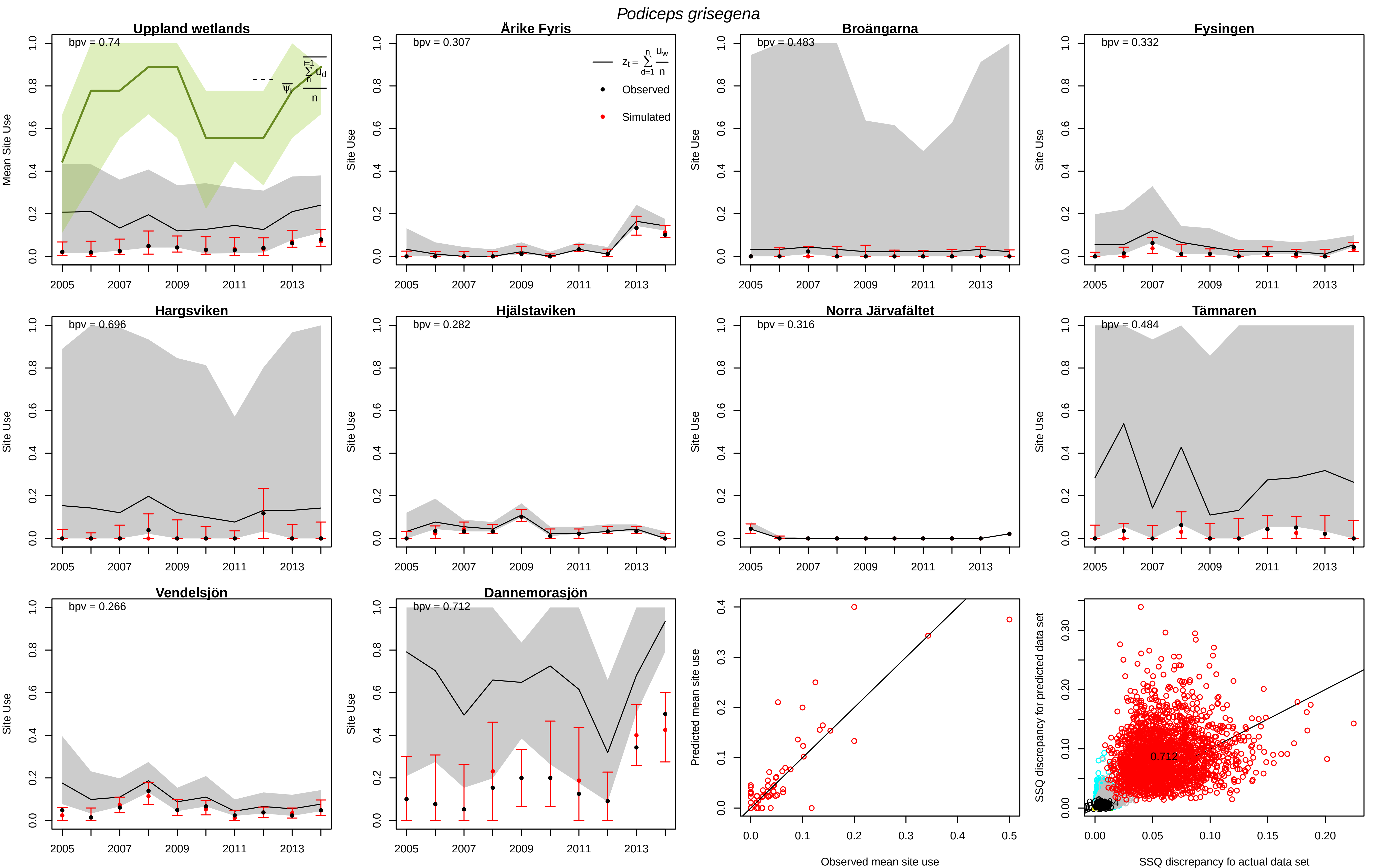
Podiceps grisegena
Uppland wetlands
bpv = 0.74
Mean Site Use
Årike Fyris
bpv = 0.307
Observed
Simulated
Broängarna
bpv = 0.483
Fysingen
bpv = 0.332
Hargsviken
bpv = 0.696
Hjälstaviken
bpv = 0.282
Norra Järvafältet
bpv = 0.316
Tämnaren
bpv = 0.484
Vendelsjön
bpv = 0.266
Dannemorasjön
bpv = 0.712
Site Use
Predicted mean site use
Observed mean site use
0.712
SSQ discrepancy for predicted data set
SSQ discrepancy fo actual data set

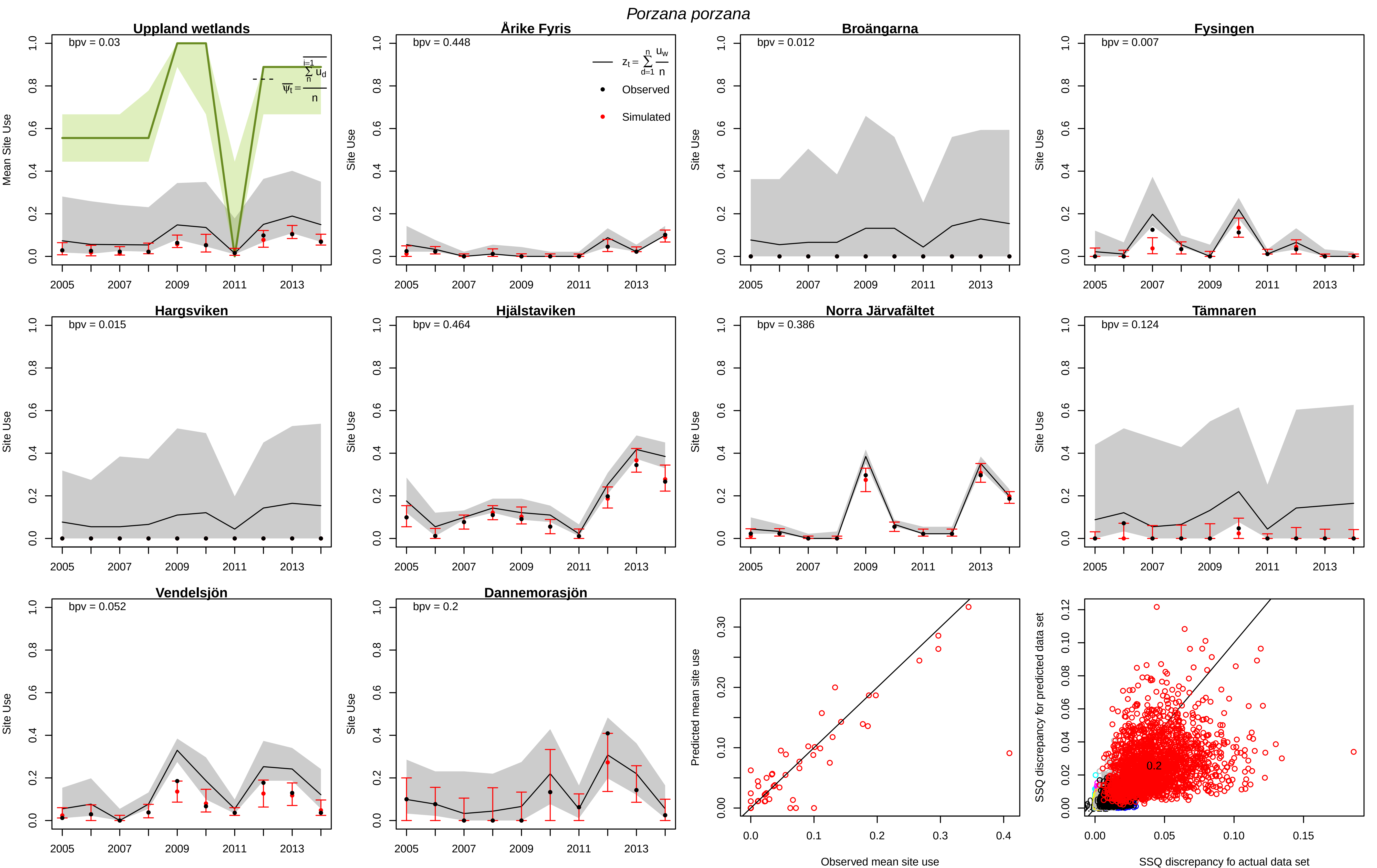

Porzana porzana
Uppland wetlands
bpv = 0.03
Mean Site Use
Årike Fyris
bpv = 0.448
Observed
Simulated
Broängarna
bpv = 0.012
Fysingen
bpv = 0.007
Hargsviken
bpv = 0.015
Hjälstaviken
bpv = 0.464
Norra Järvafältet
bpv = 0.386
Tämnaren
bpv = 0.124
Vendelsjön
bpv = 0.052
Dannemorasjön
bpv = 0.2
Site Use
Predicted mean site use
Observed mean site use
SSQ discrepancy for predicted data set
SSQ discrepancy fo actual data set
0.2

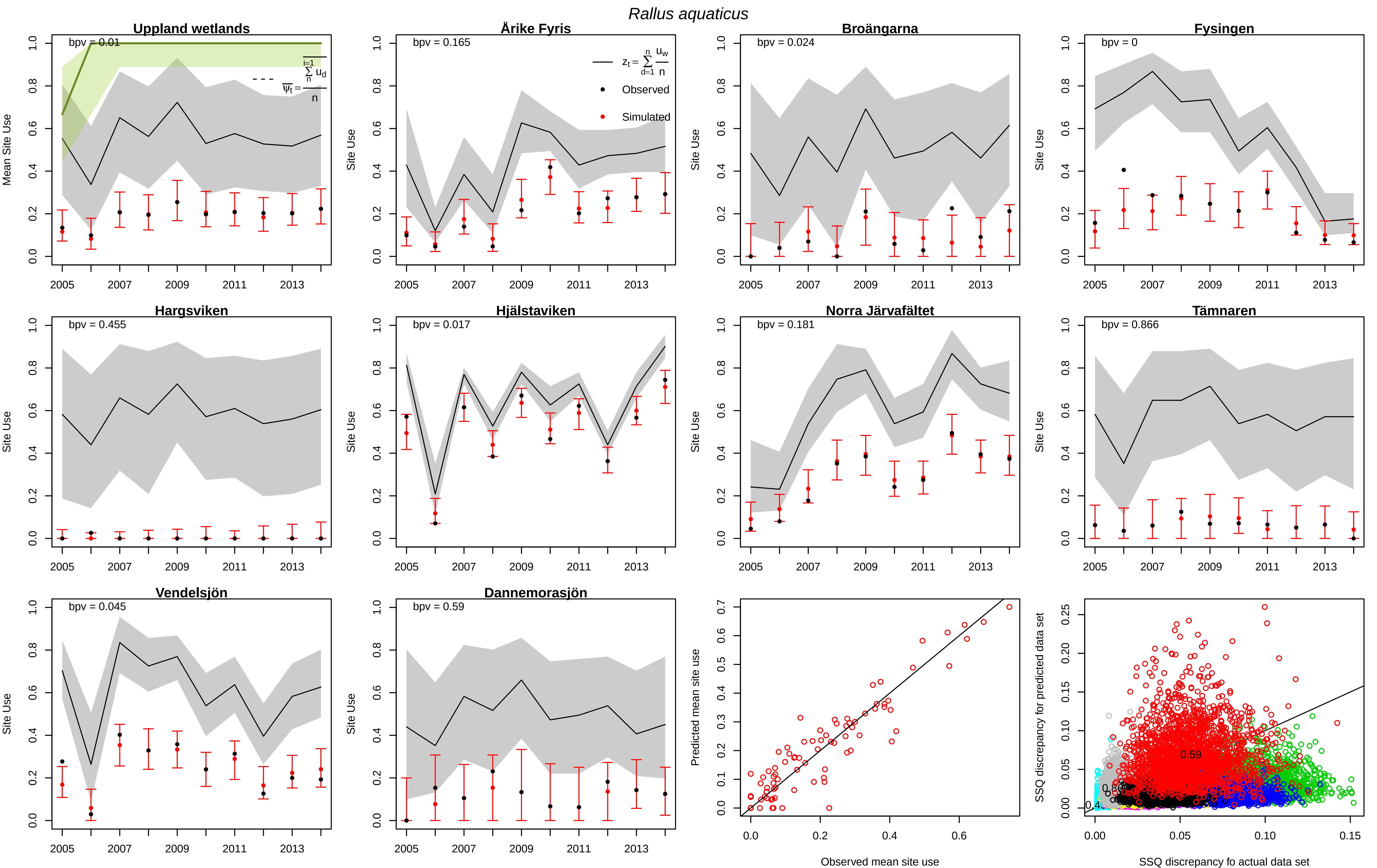

Rallus aquaticus
Uppland wetlands
bpv = 0.01
Mean Site Use
Årike Fyris
bpv = 0.165
Observed
Simulated
Broängarna
bpv = 0.024
Fysingen
bpv = 0
Hargsviken
bpv = 0.455
Hjälstaviken
bpv = 0.017
Norra Järvafältet
bpv = 0.181
Tämnaren
bpv = 0.866
Vendelsjön
bpv = 0.045
Dannemorasjön
bpv = 0.59
Site Use
2005
2007
2009
2011
2013
Predicted mean site use
Observed mean site use
SSQ discrepancy for predicted data set
SSQ discrepancy fo actual data set
0.59

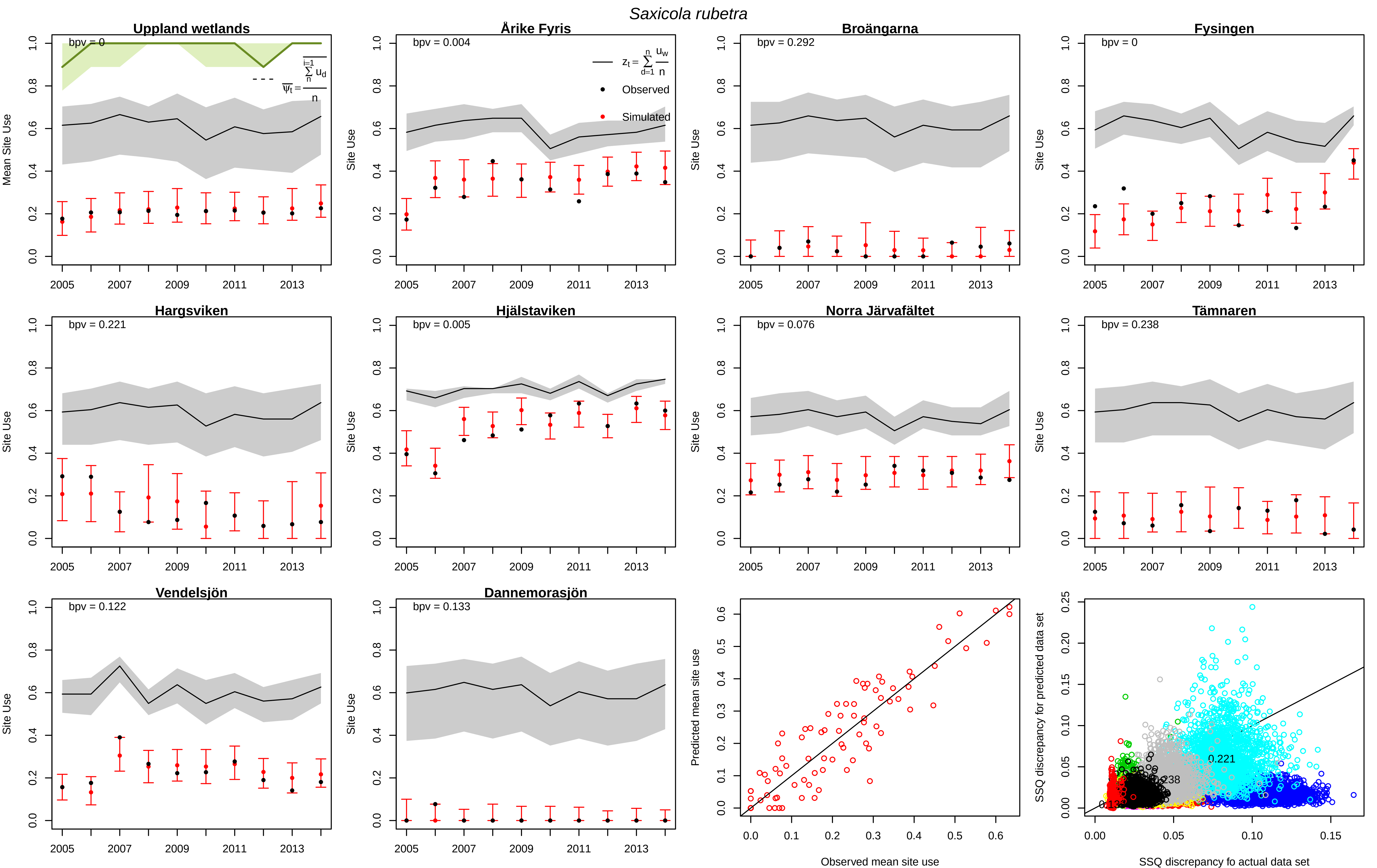
Saxicola rubetra
Uppland wetlands
bpv = 0
Mean Site Use
Årike Fyris
bpv = 0.004
Observed
Simulated
Broängarna
bpv = 0.292
Fysingen
bpv = 0
Hargsviken
bpv = 0.221
Hjälstaviken
bpv = 0.005
Norra Järvafältet
bpv = 0.076
Tämnaren
bpv = 0.238
Vendelsjön
bpv = 0.122
Dannemorasjön
bpv = 0.133
Site Use
2005
2007
2009
2011
2013
Predicted mean site use
Observed mean site use
SSQ discrepancy for predicted data set
SSQ discrepancy fo actual data set
0.221
0.238

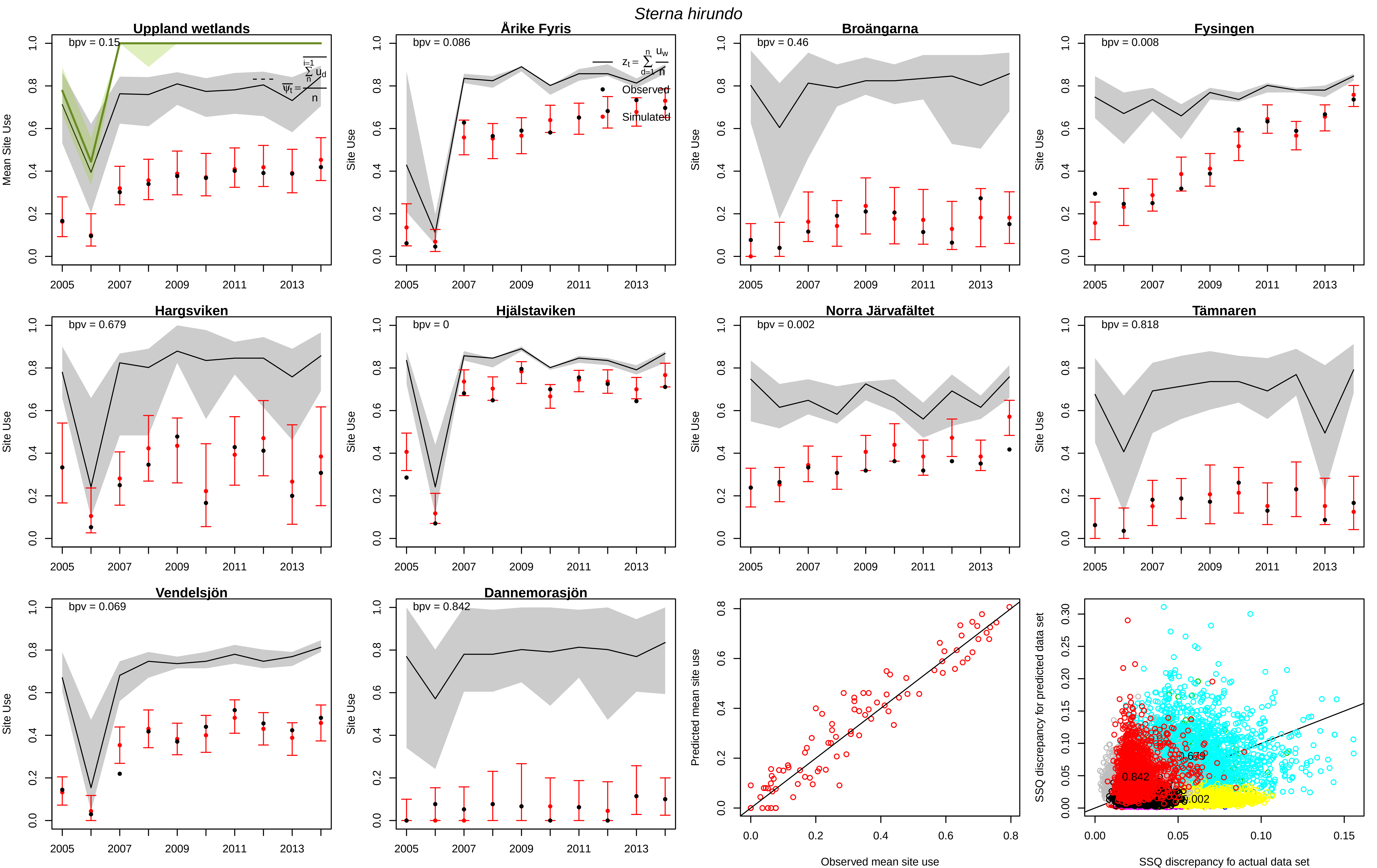
Sterna hirundo
Uppland wetlands
bpv = 0.15
Mean Site Use
Årike Fyris
bpv = 0.086
Observed
Simulated
Broängarna
bpv = 0.46
Fysingen
bpv = 0.008
Hargsviken
bpv = 0.679
Hjälstaviken
bpv = 0
Norra Järvafältet
bpv = 0.002
Tämnaren
bpv = 0.818
Vendelsjön
bpv = 0.069
Dannemorasjön
bpv = 0.842
Site Use
Predicted mean site use
Observed mean site use
SSQ discrepancy for predicted data set
SSQ discrepancy fo actual data set
0.842
0.679
0.002

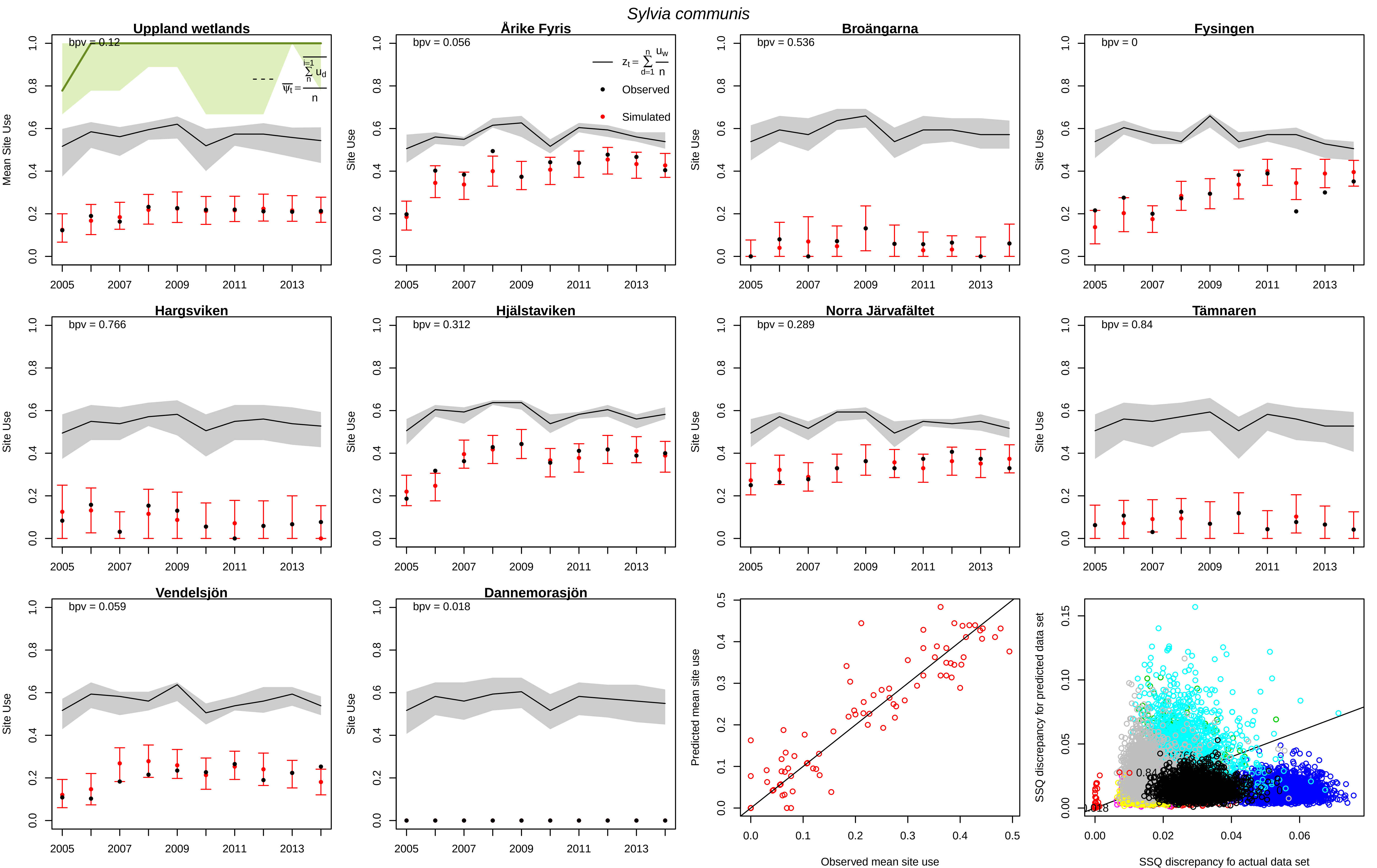

Sylvia communis
Uppland wetlands
bpv = 0.12
Mean Site Use
Årike Fyris
bpv = 0.056
Observed
Simulated
Broängarna
bpv = 0.536
Fysingen
bpv = 0
Hargsviken
bpv = 0.766
Hjälstaviken
bpv = 0.312
Norra Järvafältet
bpv = 0.289
Tämnaren
bpv = 0.84
Vendelsjön
bpv = 0.059
Dannemorasjön
bpv = 0.018
Site Use
Predicted mean site use
Observed mean site use
SSQ discrepancy for predicted data set
SSQ discrepancy fo actual data set

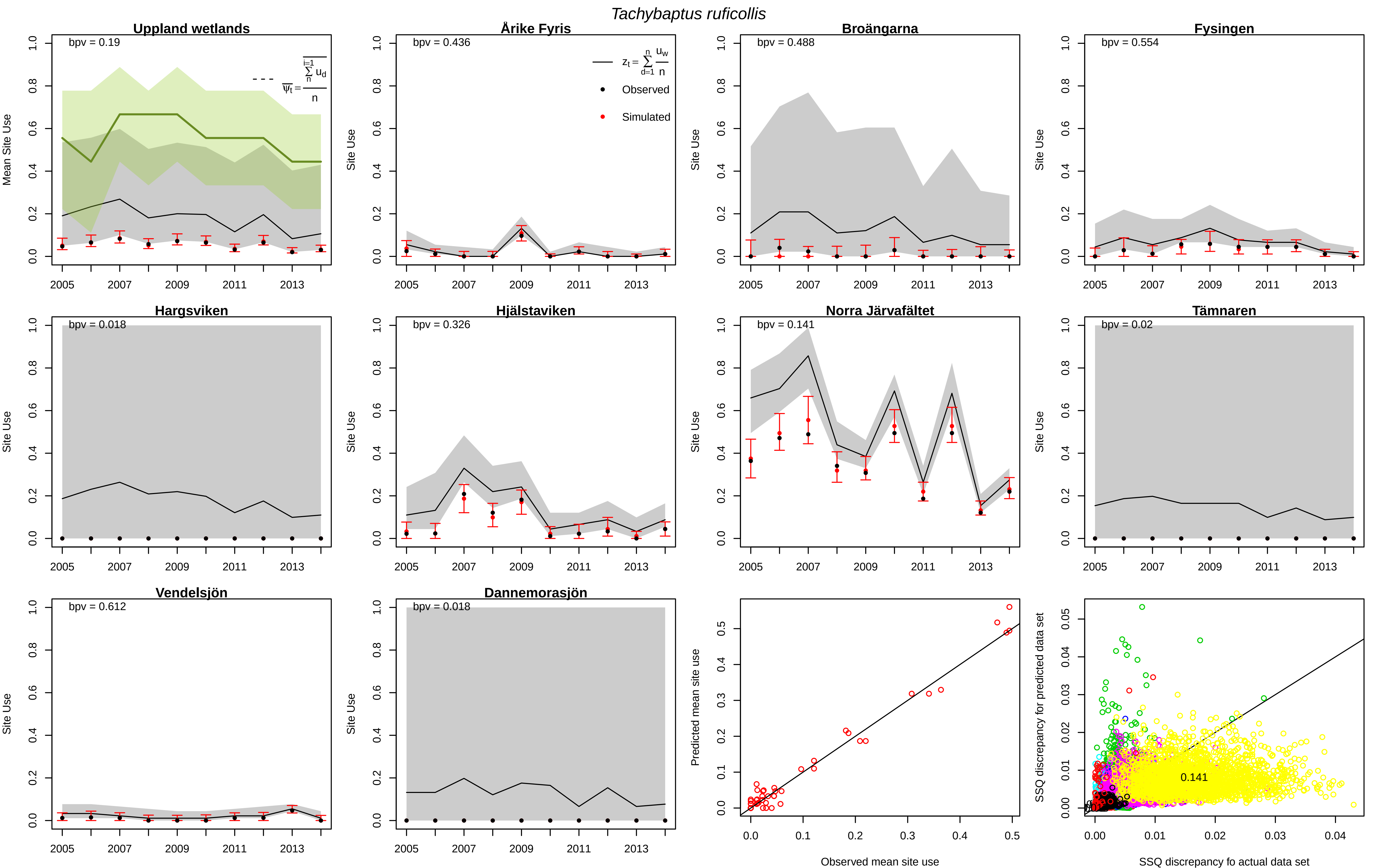

Tachybaptus ruficollis
Uppland wetlands
bpv = 0.19
Mean Site Use
Årike Fyris
bpv = 0.436
Observed
Simulated
Broängarna
bpv = 0.488
Fysingen
bpv = 0.554
Hargsviken
bpv = 0.018
Hjälstaviken
bpv = 0.326
Norra Järvafältet
bpv = 0.141
Tämnaren
bpv = 0.02
Vendelsjön
bpv = 0.612
Dannemorasjön
bpv = 0.018
Site Use
Predicted mean site use
Observed mean site use
SSQ discrepancy for predicted data set
SSQ discrepancy fo actual data set
0.141

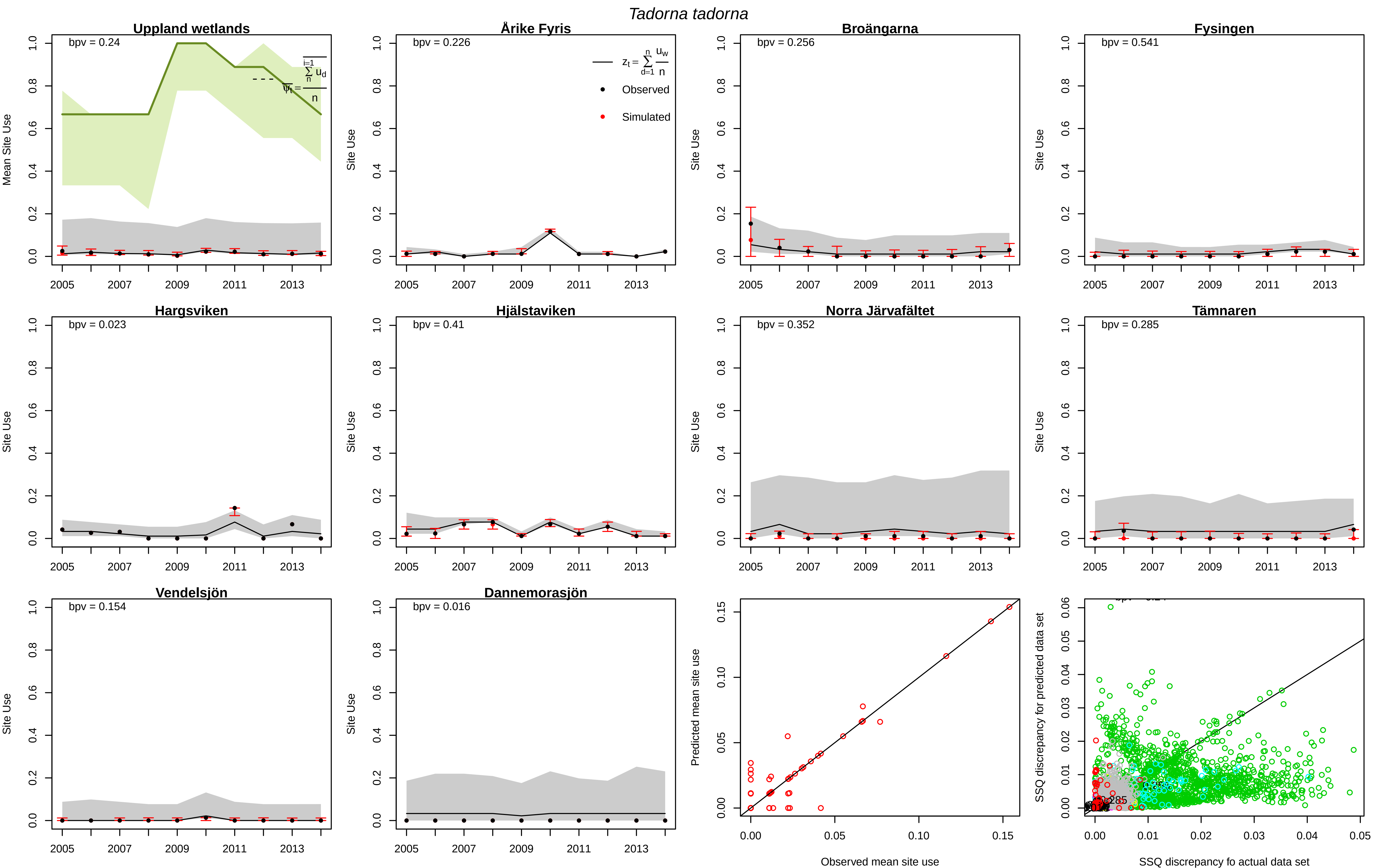

Tadorna tadorna
Uppland wetlands
bpv = 0.24
Mean Site Use
Årike Fyris
bpv = 0.226
Observed
Simulated
Broängarna
bpv = 0.256
Fysingen
bpv = 0.541
Hargsviken
bpv = 0.023
Hjälstaviken
bpv = 0.41
Norra Järvafältet
bpv = 0.352
Tämnaren
bpv = 0.285
Vendelsjön
bpv = 0.154
Dannemorasjön
bpv = 0.016
Site Use
Predicted mean site use
Observed mean site use
SSQ discrepancy for predicted data set
SSQ discrepancy fo actual data set

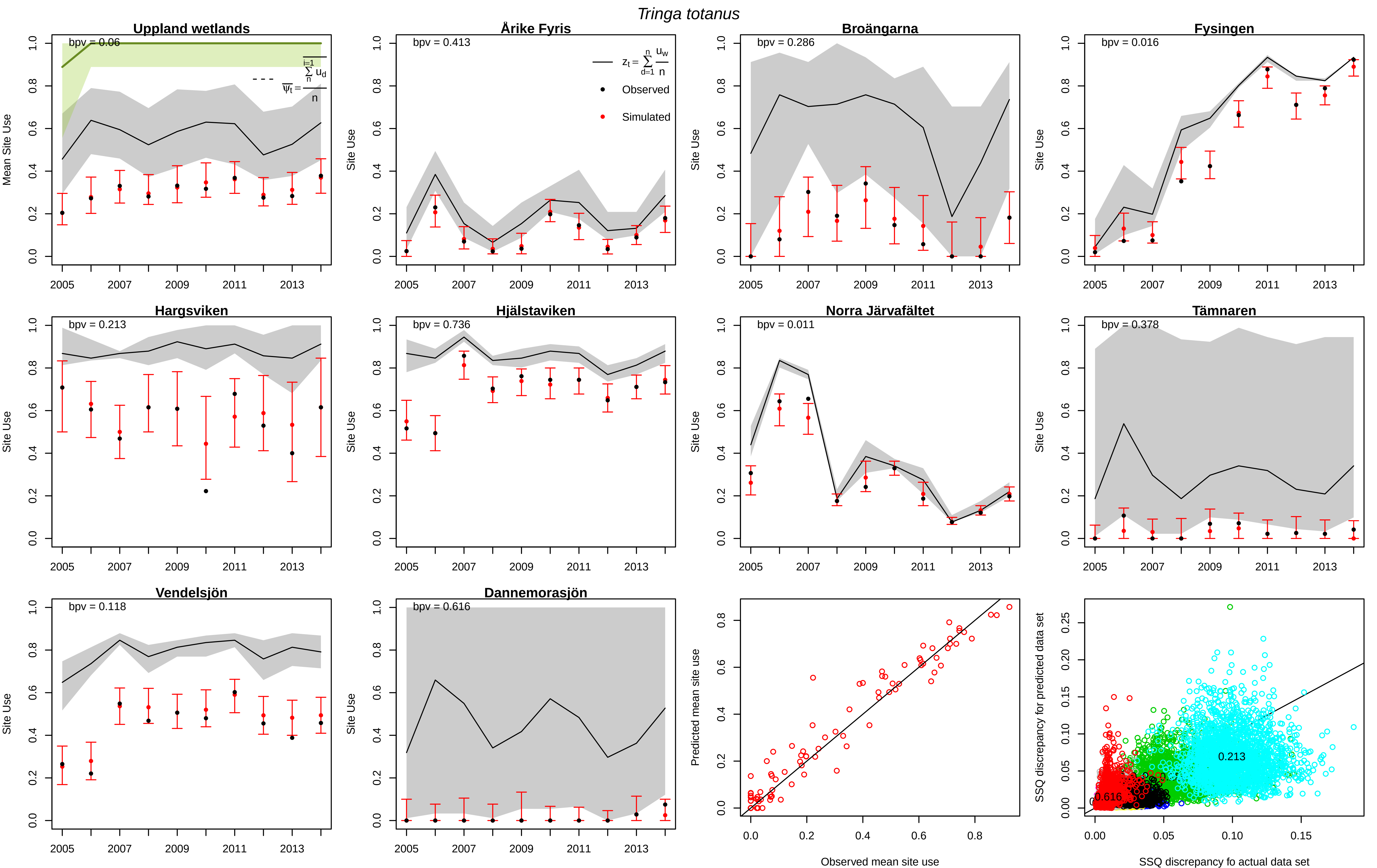

Tringa totanus
Uppland wetlands
bpv = 0.06
Mean Site Use
Årike Fyris
bpv = 0.413
Observed
Simulated
Broängarna
bpv = 0.286
Fysingen
bpv = 0.016
Hargsviken
bpv = 0.213
Hjälstaviken
bpv = 0.736
Norra Järvafältet
bpv = 0.011
Tämnaren
bpv = 0.378
Vendelsjön
bpv = 0.118
Dannemorasjön
bpv = 0.616
Site Use
Predicted mean site use
Observed mean site use
SSQ discrepancy for predicted data set
SSQ discrepancy fo actual data set
0.213
0.616

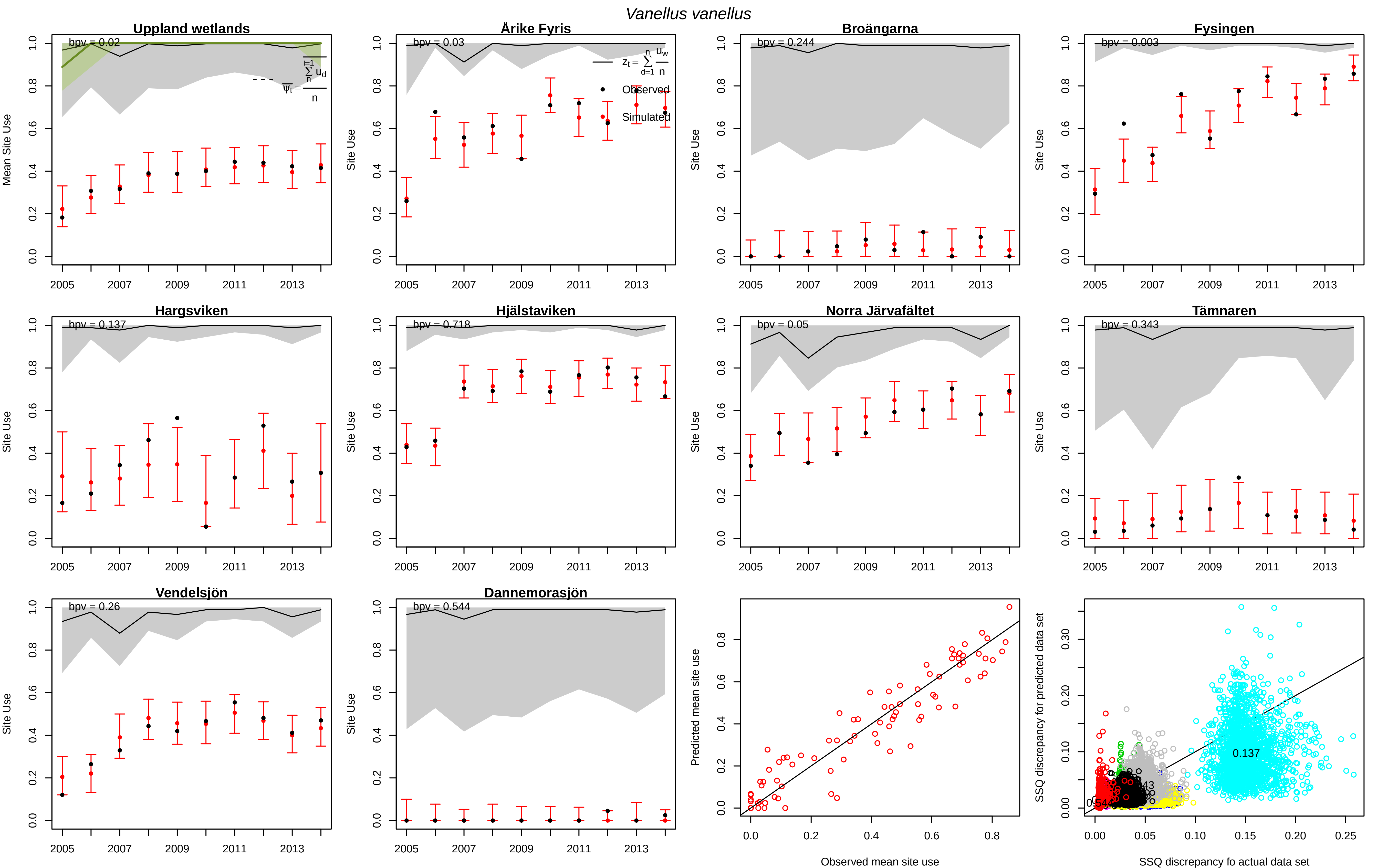

Vanellus vanellus
Uppland wetlands
bpv = 0.02
Mean Site Use
Årike Fyris
bpv = 0.03
Observed
Simulated
Broängarna
bpv = 0.244
Fysingen
bpv = 0.003
Hargsviken
bpv = 0.137
Hjälstaviken
bpv = 0.718
Norra Järvafältet
bpv = 0.05
Tämnaren
bpv = 0.343
Vendelsjön
bpv = 0.26
Dannemorasjön
bpv = 0.544
Site Use
Predicted mean site use
Observed mean site use
SSQ discrepancy for predicted data set
SSQ discrepancy fo actual data set
0.137
